# Developmental gene expression patterns driving species-specific cortical features

**DOI:** 10.1038/s41586-026-10491-x

**Published:** 2026-05-13

**Authors:** Awais Javed, Lucía Gómez, Veronica Pravata, Moein Sarhadi, Quentin Lo Giudice, Timéa Szalai, Léa Aubert, Théo Ribierre, Laurent Nguyen, Silvia Cappello, Denis Jabaudon, Esther Klingler

**Affiliations:** 1 Department of Basic Neurosciences, University of Geneva, Geneva, Switzerland; 2 Division of Physiological Genomics, Biomedical Center (BMC), Faculty of Medicine, Ludwig Maximilian University (LMU), Munich, Germany; 3 NeuroNA Human Cellular Neuroscience Platform, Fondation Campus Biotech Geneva, Geneva, Switzerland; 4 Laboratory of Molecular Regulation of Neurogenesis, GIGA Institute, University of Liège, Liège, Belgium; 5 Walloon Excellence in Life Sciences and Biotechnology (WELBIO) Department, WEL Research Institute, Wavre, Belgium; 6 Max Planck Institute of Psychiatry, Munich, Germany; 7 Munich Cluster for Systems Neurology (SyNergy), Munich, Germany; 8 Department of Clinical Neurosciences, Geneva University Hospital, Geneva, Switzerland; 9 Imagine Institute, Université Paris Cité, Paris, France; 10 VIB Center for Neuroscience Leuven, Leuven, Belgium; 11 KU Leuven Department of Neurosciences, Leuven Brain Institute, Leuven Institute for Single Cell Omics, Leuven, Belgium; 12 Present Address: Institute for Applied Data Science and Finance, Bern University of Applied Sciences, Bern, Switzerland

**Keywords:** Development of the nervous system, Neurogenesis

## Abstract

The cerebral cortex shows species-specific variations in size and organization, which probably account for distinct behavioural abilities^1^. These structural differences may reflect evolutionary changes in the developmental expression of shared genes. Here, to investigate this possibility, we used machine vision to identify and compare cell-type-specific gene expression patterns in the developing mouse and human neocortex, and in human cortical organoids. Using this approach, we identified genes with evolutionarily conserved or divergent transcriptional regulation, revealing species-specific cyto-temporal gene expression patterns. Among such genes, the transcription factor gene *JUNB* showed mutually exclusive expression in human progenitors and mouse neurons. Through cell-type-specific gain- and loss-of-function experiments in mice and human cortical organoids, we show that JUNB bidirectionally controls human cortical features, including progenitor proliferation rates, neuronal production timing and total neuronal output. We identify IRF1 as a human radial glia-specific regulator that, when expressed in mouse radial glia, activates *JUNB* and recruits human-like gene regulatory networks, demonstrating cross-species activation of poised developmental programmes. Together, these findings reveal how cyto-temporal regulation of shared genes drives species-specific cortical features, and provide a molecular framework for understanding and manipulating these evolutionary programmes.

## Abstract (web summary)

Machine learning analysis of cell-type-specific gene expression in mouse and human neocortex and human cortical organoids reveals human-specific cell-type and temporal variations in expression controlled by JUNB.

## Main

The neocortex, a hallmark of mammalian brains, is central for higher-order cognitive functions. It is composed of numerous populations of neurons that can be distinguished by several features, including their molecular identity, morphology and connectivity, which ultimately determine their function^1,2^. During neocortical development, glutamatergic neurons are born from progenitors called radial glia (RG), either directly or through transit-amplifying cells called intermediate progenitors (IPs); deep-layer neurons are born first and preferentially project subcortically, and superficial-layer neurons are born later and mainly project intracortically^3–6^. This process is largely conserved across mammals, but notable species-specific differences exist. For example, humans have a more diverse array of progenitors than mice, including basal and truncated RG, which are thought to contribute to cortical expansion and neuronal diversity^7–9^. In addition, human neurons mature particularly slowly^10^, arguably accounting for extended periods of environment-dependent plasticity. Several primate or human-specific genes influence cortical development: for example, *NOTCH2NLA*, *NOTCH2NLB*, *NOTCH2NLC* and *ARHGAP11B* enhance progenitor expansion, and *SRGAP2* and *LRRC37B* regulate synaptic maturation timing^11–14^. However, such species-specific structural gene differences are relatively rare, suggesting that temporal variations in the expression of shared genes, rather than species-specific genes, are the major source of interspecies diversity. Illustrating this view, the timing of *SATB2* expression controls whether interhemispheric axons traverse through the corpus callosum (in eutherians) or the anterior commissure (in marsupials)^15–17^. Similarly, differences in the timing of expression of similar gene sets drive intracortical projections towards the motor or secondary somatosensory cortex^18^. Such differences may also be relevant in neurodevelopmental disorders, including autism spectrum disorder, by affecting the synchrony of circuit assembly^1,19^. Thus differences in the developmental expression timing of corresponding genes can lead to meaningful differences in brain features^20^.

To systematically identify and compare cell-type-specific developmental gene expression dynamics during corticogenesis, we analysed publicly available single-cell RNA sequencing (scRNA-seq) datasets from mice, humans and human cortical organoids (Org_H_). We generated a common cell-type-specific and temporal (cyto-temporal) transcriptional framework, enabling systematic and unsupervised identification of conserved or divergent gene expression patterns. Our analysis reveals that whereas RG and immature neurons have highly conserved transcriptional programmes, IPs display marked species-specific differences, particularly in genes that control cell cycle properties and neurogenic output. This resource enables systematic identification of genes with divergent expression patterns between species and contexts, providing a foundation for understanding human features of cortical development. Using this dataset, we identify JUNB, a transcription factor with mutually exclusive expression in mouse neurons and human RG. Using gain and loss-of-function experiments in mouse and Org_H_, respectively, we find that JUNB bidirectionally controls the developmental emergence of human features, including progenitor proliferation rates and neuronal production. By mapping upstream regulators of the human *JUNB* locus, we show that IRF1, which is expressed in human but not mouse RG, can activate the mouse *Junb* promoter to induce human RG-like features, linking upstream regulation to the emergence of human cortical features. Together, these results reveal cyto-temporal differences in gene expression across species during cortical development and demonstrate how upstream molecular mechanisms regulate the emergence of species-specific functional cellular features.

**Fig. 1:**
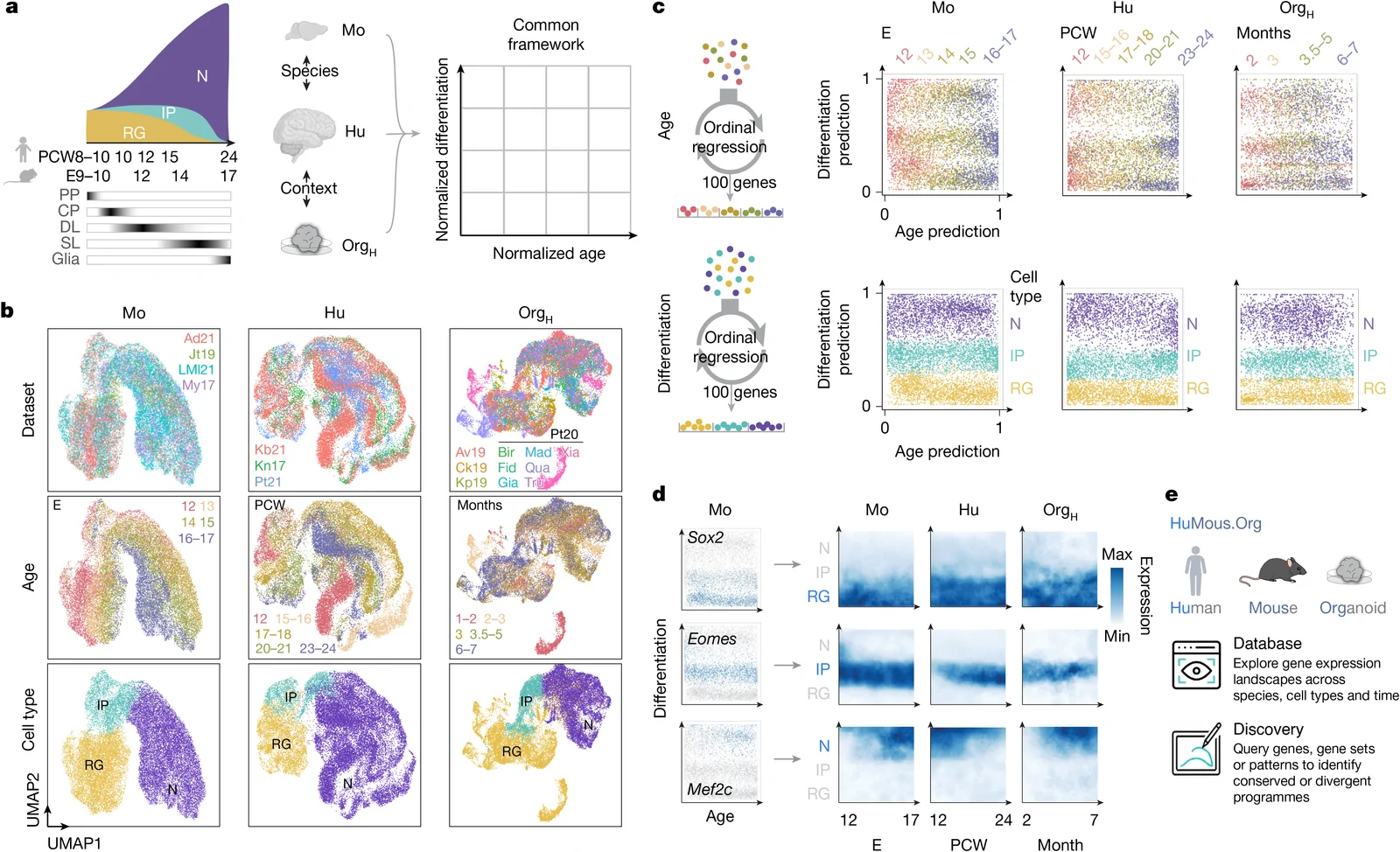
A common framework for comparing cell-type-specific developmental gene expression across species and context. **a**, Left, schematic representation of corticogenesis in mice and humans, with RG, IPs and neurons (N). Right, schematic representation of the two-dimensional cellular manifold used to analyse cyto-temporal gene expression patterns across species (mouse (Mo) versus human (Hu)) and context (human versus Org_H_). Adapted from BioRender; Pravata, M. V. https://BioRender.com/uk9hoa5 (2026). CP, cortical plate; DL, deep layers; PP, preplate; SL, superficial layers. **b**, UMAP representation of the datasets used in this study (top), age distribution of cells (middle) and cell types (bottom). **c**, Left, schematic representation of the ordinal regression model used to identify the top 100 genes driving embryonic age (top) and differentiation (bottom) identity. Right, distribution of all cells from **b** using the framework presented in **a**, right. **d**, Representative gene expression landscapes showing how the manifold enables visualization of cyto-temporal patterns through colour intensity coding of expression levels. Org_H_ time (far right; 2, 7) is presented as months. **e**, Structure and purpose of the Humous.org dataset. The data are searchable for gene discovery and database exploration at https://www.humous.org.

**Extended Data Fig. 1:**
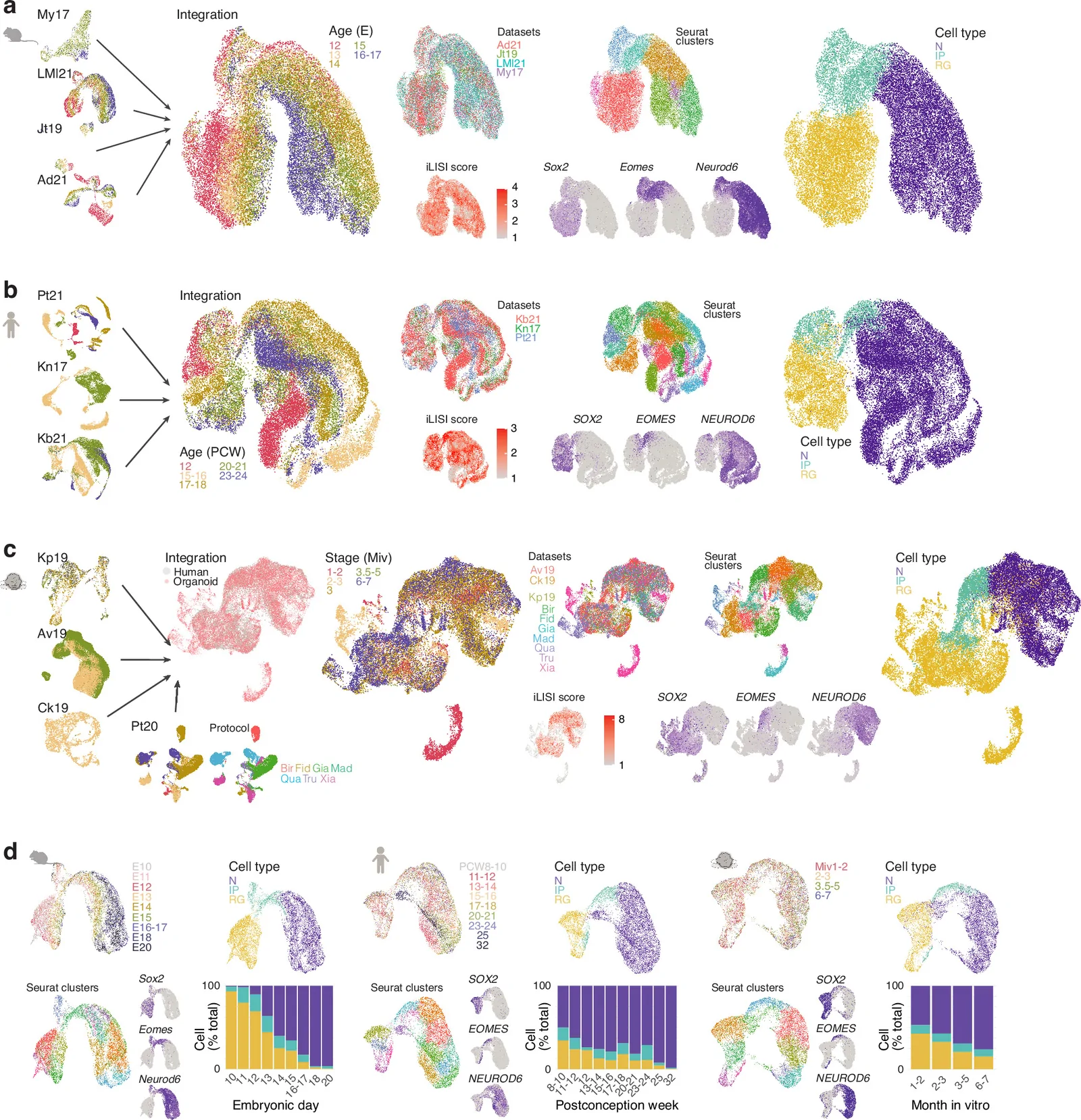
Integration of single-cell RNA sequencing datasets and cell type annotation consensus. **a**–**c**, Integration of single-cell RNA sequencing datasets from mouse (Mo) (**a**), human (Hu) (**b**) developing cortex, and human-derived cortical organoid (Org_H_) (**c**) (see Supplementary Table 1). From left to right: individual dataset UMAPs annotated by age; integrated Hu, Mo, and Org_H_ datasets (see Methods) labeled by age, dataset (top), iLISI integration score (bottom), and Seurat clusters (top right); feature plots showing expression of *SOX2*, *EOMES* and *NEUROD6*; cell type annotation consensus. **d**, Integration of extended datasets^24,38,39^ encompassing E10 to E20 in mouse, and PCW8 to PCW32 in human and comparison of RG, IP and N, defined as above, by age. Abbreviations: E, embryonic day; imN, immature neuron; IP, intermediate progenitor; matN, mature neuron; Miv, month in vitro; N, neuron; PCW, postconception week; RG, radial glia.

**Extended Data Fig. 2:**
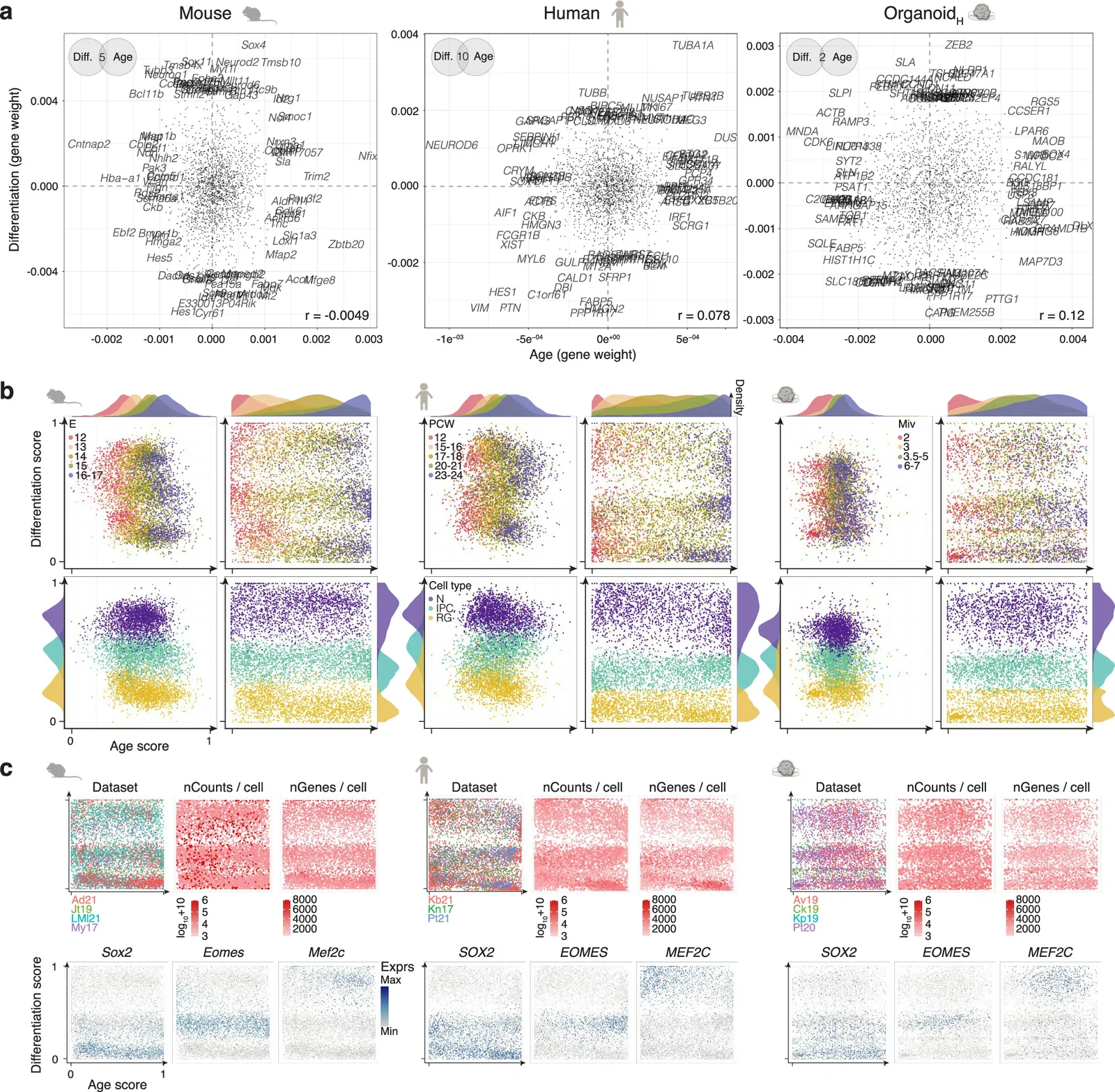
Differentiation and age prediction scores using ordinal regression models. **a**, Top gene weights in the age and differentiation mouse (left), human (middle), and Org_H_ (right) models. Number of common genes in the differentiation and age models are indicated in the Venn diagrams for each condition. Correlation between age and differentiation weights (r) is indicated at the bottom right of each plot. **b**, Single cell prediction scores for age (x axis) and differentiation (y axis) before (left) and after (right) normalization for each condition. Cells are labeled by age (top) and differentiation (bottom). Densities along age and differentiation axes are shown for each condition. **c**, Origin dataset, number of counts and genes per cell (top), and expression of *SOX2*, *EOMES* and *MEF2C* (bottom) in single cells along age and differentiation axes for each condition. Abbreviations: Diff., differentiation; E, embryonic day; Exprs, expression; IP, intermediate progenitor; Miv, month in vitro; N, neuron; PCW, postconception week; RG, radial glia.

**Extended Data Fig. 3:**
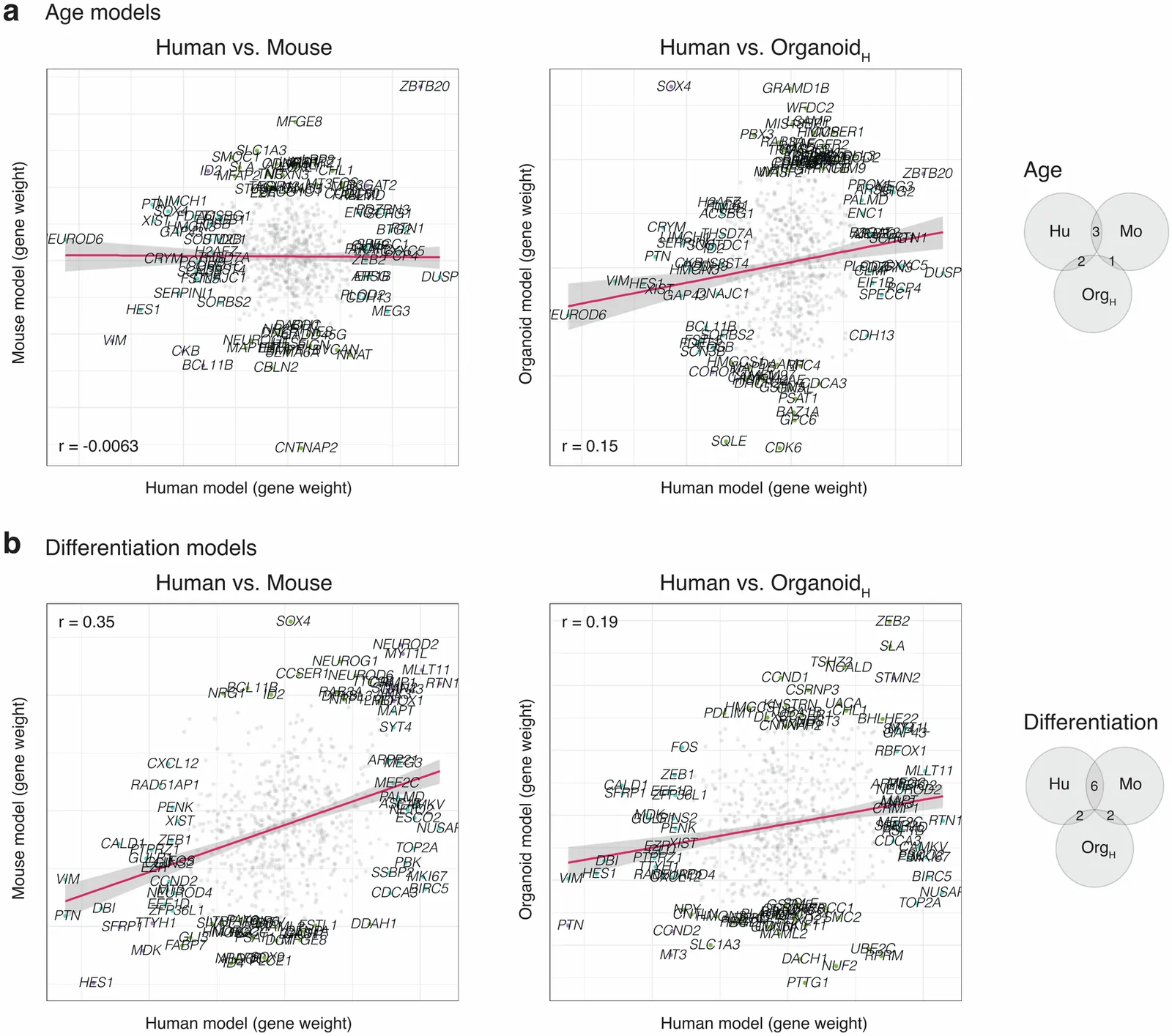
Comparison of gene weights between conditions in the age and differentiation models. **a**, Gene weight comparisons in human *versus* mouse (Mo) (left) and human (Hu) versus human cortical organoid (Org_H_) (right) age models. Correlations between gene weights are indicated (r). Right: Common top genes between conditions. **b**, Gene weight comparisons in Hu *versus* Mo (left) and Hu versus Org_H_ (right) differentiation models. Correlations between gene weights are indicated (r). Right: Common top genes between conditions. Fitted linear regression (magenta) and 95% confidence interval (grey) are shown (**a**,**b**).

**Extended Data Fig. 4:**
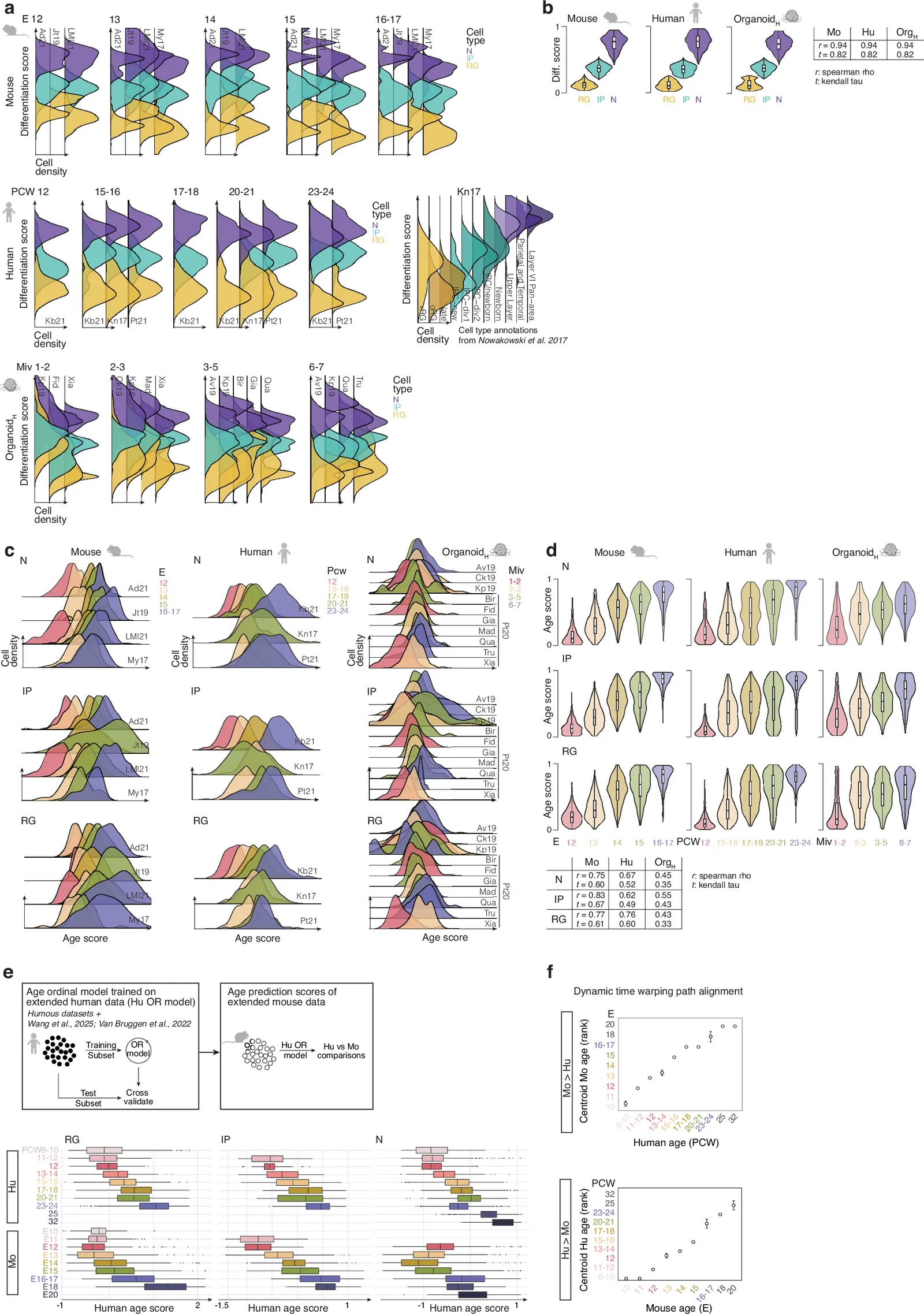
Differentiation predictions by dataset-age and age predictions by dataset-cell type. **a**, Density of cells along the differentiation prediction axis by cell type annotation, for each dataset and age at collection (top, N; middle, IP; bottom, RG). **b**, Spearman rho and kendall tau scores for differentiation scores compared to cell type annotation per condition (Mouse (Mo): *n* = 2425 cells/cell type; Human (Hu): *n* = 2450 RG and N, *n* = 2280 IP; Human cortical organoid (Org_H_): *n* = 1800 cells/cell type). Data are presented as median ± interquartile range (IQR, boxes) and 1.5x IQR (whiskers). **c**, Density of cells along the age prediction axis by their age at collection, for each dataset (see Supplementary Table 1) and cell type (top, N; middle, IP; bottom, RG). **d**, Comparison of age scores and actual ages at collection and spearman rho and kendall tau correlation values per condition and cell type (Mouse (Mo): *n* = 485 cells/age/cell type; Human (Hu): *n* = 490 cells/age/cell type, except for PCW12 IP: *n* = 320 cells; Human cortical organoid (Org_H_): *n* = 450 cells/ age/cell type). **e**, Comparisons of human age prediction scores (*i.e*., calculated from Hu ordinal regression models built by cell type, using extended datasets^26–28,38,39^, RG = 630 cells/age, IP = 275 cells/age, N = 1047 cells/age) and actual ages at collection in Hu and Mo (see Methods). Data are presented as median ± IQR (boxes) and 1.5x IQR (whiskers) (**b**,**c**). **f**, Dynamic time warping alignment using Mo and Hu datasets^26–28,38,39^. Data are presented as centroid ± IQR. Abbreviations: PCW, postconception week; E, embryonic day; Miv, month in vitro; N, neuron; IP, intermediate progenitor; RG, radial glia; OR, ordinal regression.

**Extended Data Fig. 5:**
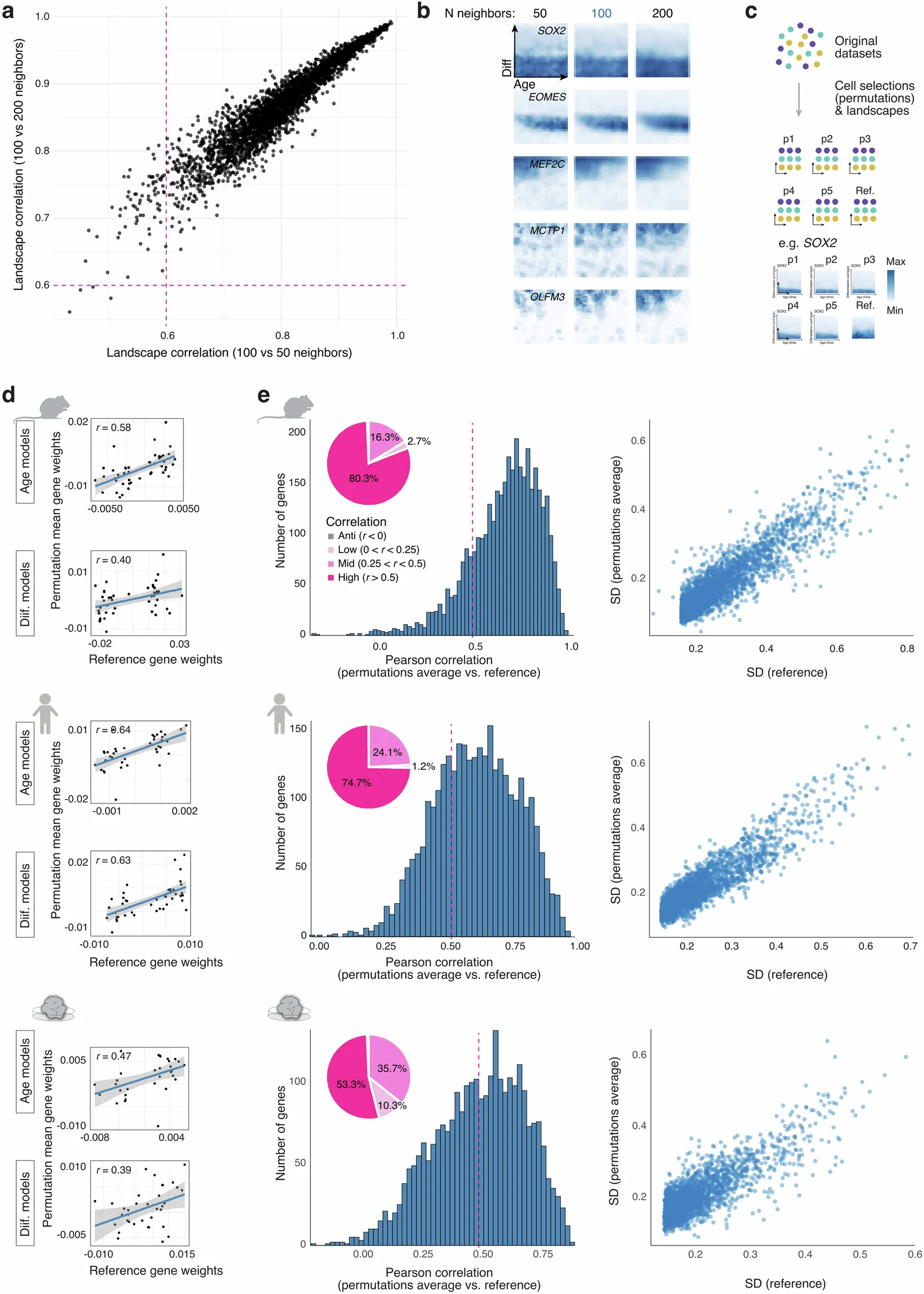
Gene expression landscape robustness. **a**, Correlation values between patterned landscapes generated upon expression normalization using 100 neighbors (original landscapes) and 200 or 50 neighbors. **b**, Examples of landscapes generated upon expression normalization using 50, 100 or 200 neighbors. **c**, Schematic of the approach. Different cell selections were made using permutations, ordinal model age and differentiation scores were calculated for each permutation and resulting gene expression landscapes were compared to the reference (ref) landscapes used in www.humous.org. Bottom, example of *Sox2* expression in Mo with the different permutations (p). **d**, Correlations between top gene weights from reference and permutation average weights for age and differentiation models in each condition. Fitted linear regression (blue) and 95% confidence interval (grey) are shown. **e**, Left: Correlation between permutations’ average and reference gene expression landscapes. Pie charts represent the proportion of anti-, lowly-, mid-, and highly correlated landscapes in each condition. Right: Comparison of landscape standard deviation (s.d.) between permutations’ average and reference gene expression landscapes in each condition.

**Extended Data Fig. 6:**
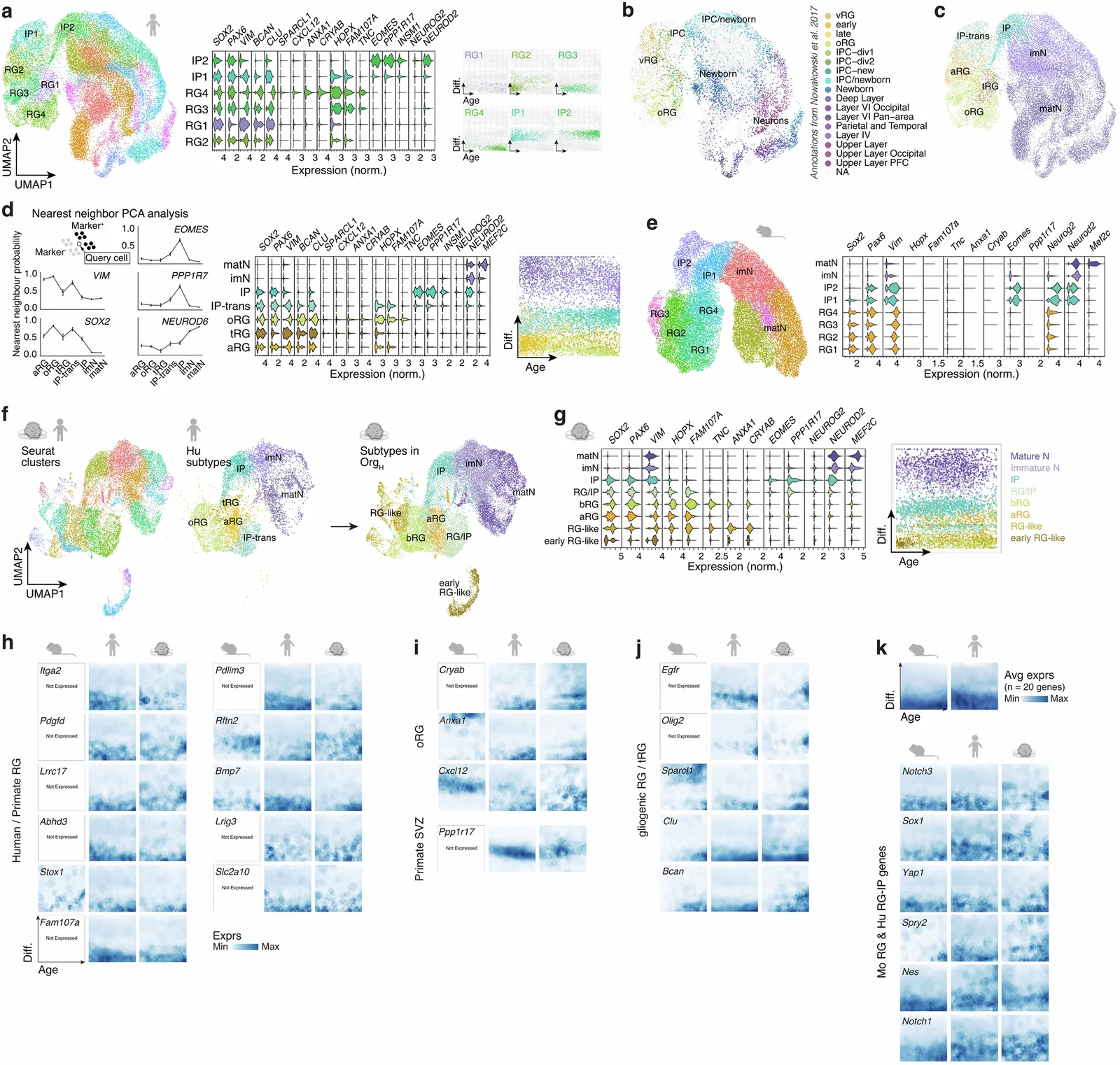
Human progenitor heterogeneity in the humous.org framework. **a**, Hu clusters of RG and IP in the integrated UMAP (left) with corresponding marker expression (middle) and distribution of progenitor clusters along age and differentiation axes (right). **b**, Original annotations from ref. ^26^. **c**, Cell type curated annotations in the integrated UMAP. **d**, Schematic of the nearest-neighbor (NN) probability analysis. Cells (aRG = 1501 cells, oRG = 2748 cells, tRG = 644 cells, IP-trans = 1183 cells, IP = 1372 cells, imN = 8616 cells, mN = 12803 cells) were embedded in integrated principal component (PC) space and assigned to their closest transcriptional neighbor. NN probability profiles for *VIM*, *SOX2*, *EOMES*, *PPP1R17*, and *NEUROD6* are shown across the RG–IP–neuronal continuum (aRG to matN). Points indicate mean probabilities. Error bars denote 95% confidence intervals. **e**, Mo clusters of RG and IP in the integrated UMAP (left) with corresponding marker expression (right). **f**, Left: unsupervised Seurat clusters of Org_H_ and Hu integrated datasets. Middle: Hu annotations. In grey are Org_H_ cells. Right: Subtypes annotations in Org_H_ datasets. **g**, Marker expression in Org_H_ cell subtypes (left) and distribution along age and differentiation axes (right). **h**–**j**, Expression landscapes of genes specific to human/primate RG (**h**), oRG (**i**, top) and primate SVZ (**i**, bottom), truncated and gliogenic RG (**j**). **k**, Expression landscapes of genes expressed in Mo RG and Hu RG-IP. Top: Averaged expression (Avg exprs) of 20 random genes. Bottom: Examples of single gene landscapes. Abbreviations: imN, immature neuron; IP, intermediate progenitor; IPC, IP cell; matN, mature neuron; PFC prefrontal cortex; RG, radial glia; aRG, apical RG; bRG, basal RG; oRG, outer subventricular zone RG; SVZ, subventricular zone; tRG, truncated RG; vRG, ventricular zone RG.

To normalize and compare developmental orthologue gene expression dynamics between species (*Mus musculus* versus* Homo sapiens*) and context (in vivo versus in vitro), we created a two-dimensional cellular manifold. Cortical cells were positioned on the basis of their gene expression along two orthogonal axes: normalized age (*x* axis, from early to late corticogenesis) and differentiation state (*y* axis, from RG to IP and glutamatergic neurons)^1,21^ (Fig. 1a). Whereas cross-species comparison of major differentiation cell types (RG, IPs and neurons) is facilitated by conserved markers, aligning developmental timing across species is challenging, partly because of mosaic evolution, whereby distinct cell types may mature at different rates^22^. We therefore aligned datasets taking conserved corticogenic milestones as reference (Methods). For each condition, we integrated several scRNA-seq datasets from developmental stages covering layer 6 (L6) to L2/3 neurogenesis, when RG, IPs and neurons were present in substantial numbers: embryonic day 12 (E12) to E17 in mouse (four datasets, 30,000 cells)^21,23–25^; post-conception week 12 (PCW12) to PCW24 in human (three datasets, 28,867 cells)^26–28^; and 2–7 months in vitro for Org_H_ (four datasets, in which RG:IP:neuron proportions were consistent with the Human Neural Organoid Cell Atlas^29^, 28,045 cells)^30–33^ (Fig. 1b, Extended Data Fig. 1 and Supplementary Table 1; see Methods on the temporal alignment strategy). Original cell-type annotations were curated into RG, IP and neuron classes after integration of the datasets on the basis of unsupervised clustering and expression of *SOX2*, *EOMES* and *NEUROD6* (Extended Data Figs. 1 and 6). In human, unsupervised clustering of progenitors combined with marker expression analysis and original dataset annotations^26,34^ revealed RG clusters that were enriched in ventricular zone (VZ) RG, outer subventricular zone (OSVZ) RG and truncated RG, with the latter two emerging during mid-corticogenesis and increasing with age (Extended Data Fig. 6a–d). For cross-condition comparative analyses, these human RG subtypes were grouped and treated collectively as RG. In all conditions at late time points, RG started expressing gliogenic markers (for example, *CLU*, *SPARCL1* and *BCAN*^35,36^) consistent with known timing of gliogenesis^24,37^ (Extended Data Fig. 6j). Balanced numbers of cells were randomly sampled per cell type and age (*n* = 7,275 total in mouse, 7,180 in human and 5,400 in Org_H_), and cell position within the manifold was determined using ordinal regression models that predict age (*x* axis) and differentiation state (*y* axis) by condition (Fig. 1c, Extended Data Figs. 2–4, Supplementary Table 2 and Methods). Age matches between species were further supported by ordinal models and dynamic time-warping path alignment using datasets at additional ages (E10–E11 and E18–E20 for mouse^24^, PCW8–PCW11 and PCW25–PCW32 for human^38,39^) (Extended Data Fig. 4e,f). This layout enabled visualization of cyto-temporal gene expression as distinct genetic ‘landscapes’ by colour coding cells on the basis of normalized gene expression levels (Fig. 1d and Methods). These landscapes were stable after repeated random cell samplings and when external validation datasets were used, confirming robustness (Extended Data Fig. 5 and Methods). All data are available in an interactive format at https://www.humous.org (Fig. 1e and Extended Data Fig. 6h–k).

**Fig. 2:**
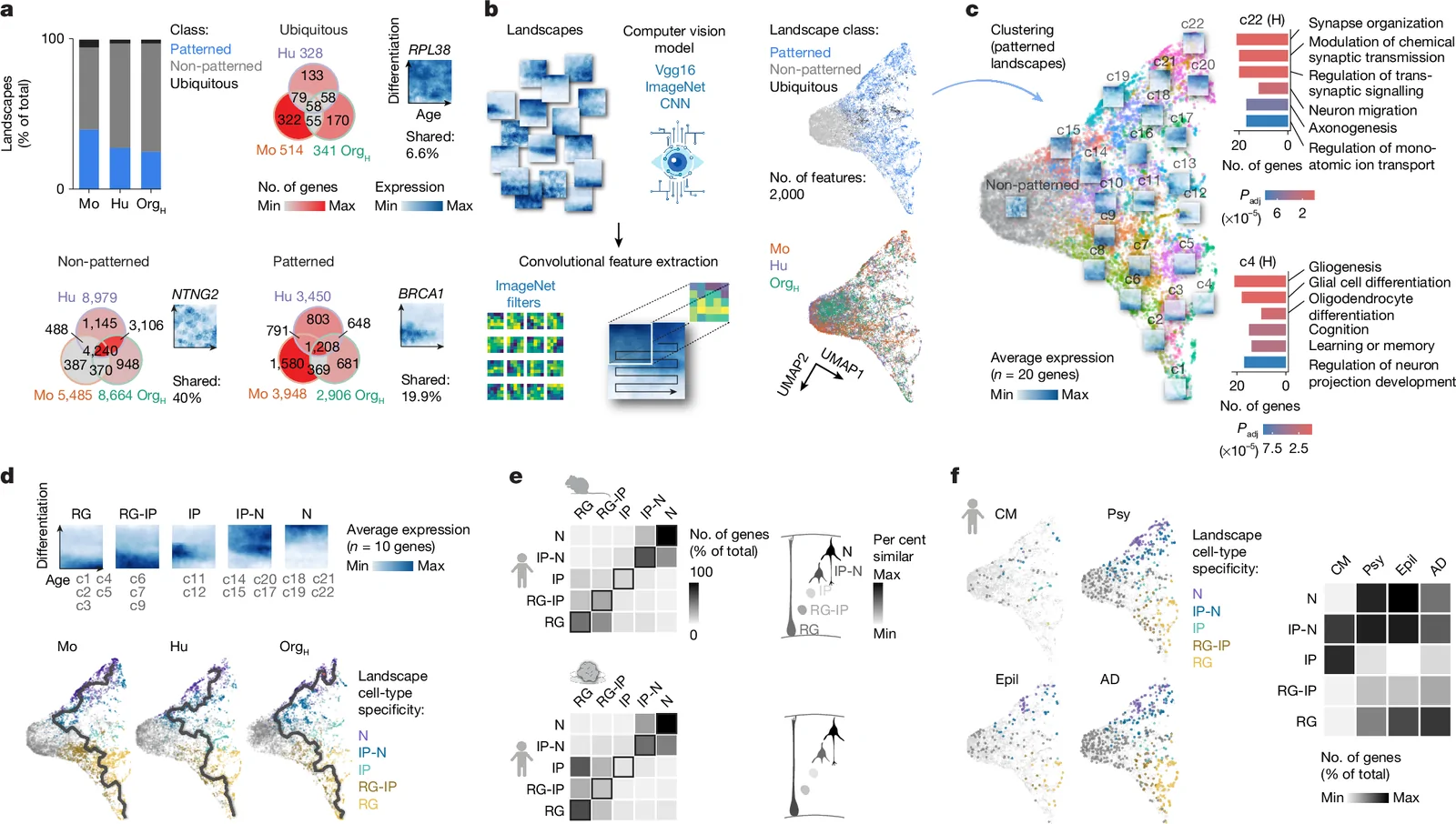
Systematic analysis of developmental gene expression patterns reveals cell-type-specific conservation. **a**, Classification of gene expression landscapes on the basis of s.d. and entropy metrics, revealing three main profiles: non-patterned (67.4%), ubiquitous (3.1%) and patterned (29.5%). Max, maximum; min, minimum. **b**, Left, schematic representation of the landscape analytic pipeline. Landscapes were clustered on the basis of 2,000 computer vision-extracted features. Right, UMAP visualization of landscape distributions. Each dot represents a single landscape. CNN, convolutional neural network. **c**, Left, organization of the 22 identified landscape clusters. Right, example landscapes from key clusters showing associated biological processes (human data). Enrichment was tested using a one-sided hypergeometric test and *P* values were adjusted for multiple comparisons using the Benjamini–Hochberg false discovery rate method. Terms with adjusted *P* < 0.05 were considered significant. *P*_adj_, adjusted *P* value. **d**, Top, landscape clusters categorized on the basis of cell-type-specific expression patterns. Bottom, pseudotime alignment using Monocle showing the differentiation trajectory in the landscape UMAP. **e**, Left, cross comparison of cell-type-specific expression patterns across species (mouse versus human; top) and context (human versus Org_H_; bottom). Right, schematic representation of the data shown in the cross correlations. **f**, Left, landscape UMAPs highlighting disease-associated genes (human data). AD, Alzheimer’s disease; CM, cortical malformation (microcephaly, megalencephaly, lissencephaly, polymicrogyria and heterotopia); Epil, epilepsy; Psy, neuropsychiatric diseases(autism spectrum disorder and schizophrenia). Right: cell-type-specific expression of disease-associated genes. Schematics and illustrations adapted from BioRender; Pravata, M. V. https://BioRender.com/spvnnlp (2026).

**Extended Data Fig. 7:**
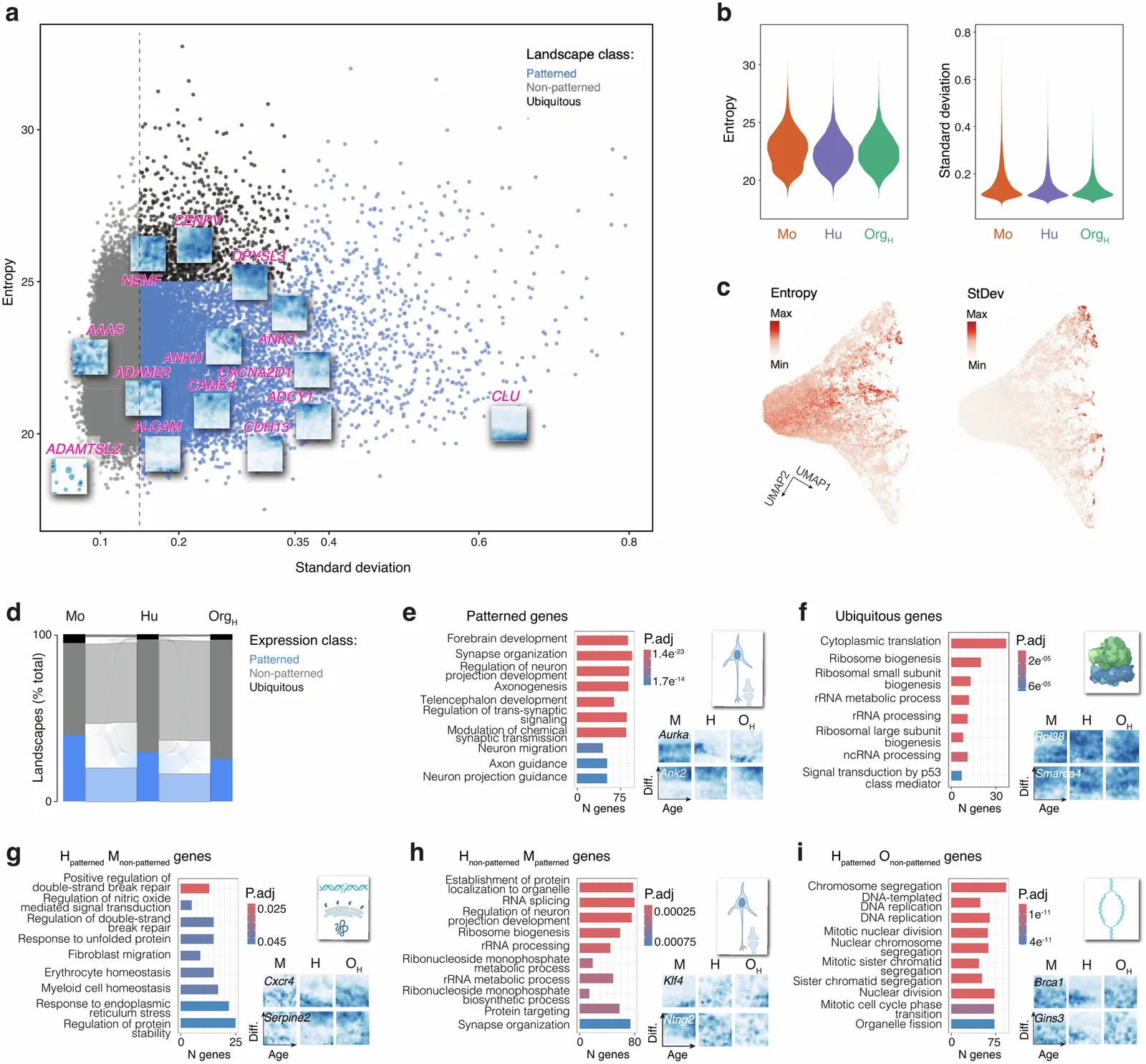
Entropy and standard deviation define classes of gene expression landscapes and ontologies. **a**, Entropy and standard deviation of all landscapes and definition of landscape classes. Representative examples of human gene expression landscapes are shown. **b**, Entropy (left) and standard deviation (right) distributions of all landscapes in Mo, Hu, and Org_H_. **c**, Entropy (left) and standard deviation (StDev, right) of all landscapes in the UMAP space. **d**, Comparison of landscape classes between conditions for corresponding genes. **e**,**f**, Top 10 ontologies of genes with patterned (**e**) and ubiquitous (**f**) expression landscapes in all conditions. **g**, Top 10 ontologies of genes with patterned expression landscapes in Hu (H_patterned_) which are non-patterned in Mo (M_non-patterned_). **h**, Top 10 ontologies of genes with non-patterned expression landscapes in Hu (H_non-patterned_) which are patterned in Mo (M_patterned_). **i**, Top 10 ontologies of genes with patterned expression landscapes in Hu (H_patterned_) which are non-patterned in Org_H_ (O_non-patterned_). In **e**–**i**, Insets summarize the main gene ontologies (top right) and examples of gene expression landscapes are shown for each condition (bottom right). Gene Ontology over-representation analysis for biological processes was performed using the enrichGO function from the clusterProfiler package. Enrichment was tested using a one-sided hypergeometric test, and *P* values were adjusted for multiple comparisons using the Benjamini–Hochberg FDR method. Terms with adjusted *P* < 0.05 were considered significant. Only significant gene ontologies are shown. Representative schemes (**e**–**i**) were adapted from BioRender; Pravata, M. V. https://BioRender.com/ocbqjw9 (2026). Abbreviations: p.adj, adjusted *P *value; Diff., differentiation.

As a first step in comparing landscapes, we determined their cyto-temporal organization using the s.d. and entropy of expression as parameters. This distinguished three profiles: non-patterned, ubiquitous and patterned (Extended Data Fig. 7 and Methods). Most landscapes (about 67.4%) did not display cyto-temporal patterning—that is, gene expression was diffuse and patchy (low s.d., low entropy) (Supplementary Table 3). About 3% of landscapes had a ubiquitous profile—they showed diffuse, homogenous expression (low s.d., high entropy). Finally, patterned landscapes (29.5% of all landscapes) showed expression with the most obvious biological relevance, as gene expression was contrasted across cells and concentrated at specific times and/or differentiation states (high s.d., low entropy) (Fig. 2a and Extended Data Fig. 7). Genes with ubiquitous landscapes were involved in generic cellular processes such as RNA processing and translation. By contrast, genes with patterned landscapes were enriched in ontologies related to more-specialized cellular functions, such as neuron migration and synapse organization (Extended Data Fig. 7e,f). The fraction of patterned landscapes was significantly lower in human than in mouse (*P* = 1.29 × 10^−126^; Fig. 2a and Extended Data Fig. 7), potentially reflecting human factors: greater pleiotropy (that is, genes functioning across more cell types and time points), additional isoforms unresolved by current sequencing, increased genetic diversity and/or more heterogeneous sample collection conditions. This differential patterning showed functional relevance: human-specific patterned genes were enriched for DNA repair and protein stability, supporting the extended need for maintenance of genome integrity during slower differentiation^40,41^, whereas mouse-specific patterned genes related to ribosome biogenesis and synapse organization, potentially enabling rapid protein synthesis and neuronal maturation during a comparatively compressed development (Extended Data Fig. 7g,h). Genes patterned in human but not in Org_H_ were enriched for chromosome segregation, suggesting in vitro environmental influences on cell division^42^ (Extended Data Fig. 7i).

**Extended Data Fig. 8:**
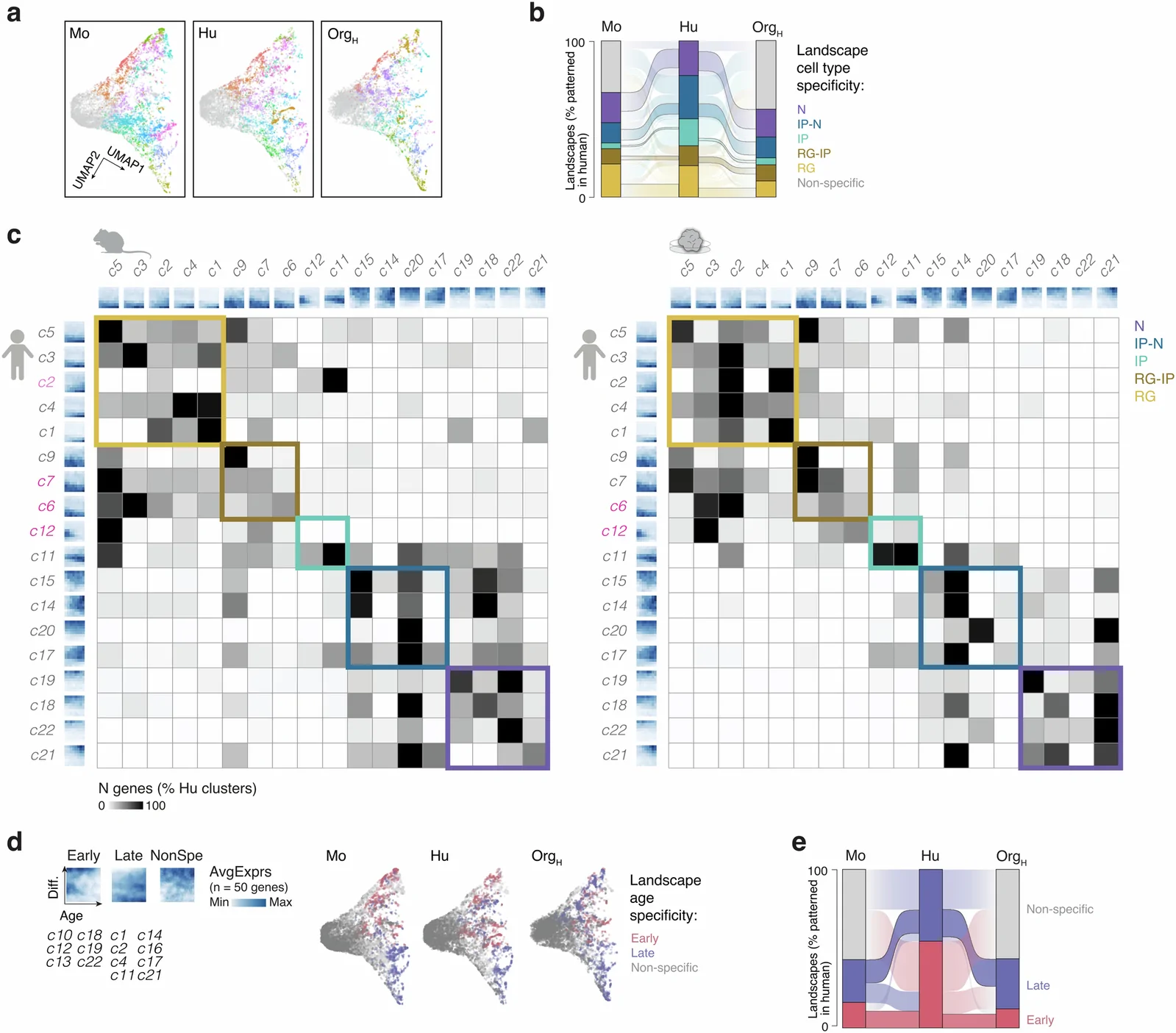
Cell type- and age-specific landscapes across conditions. **a**, Landscapes clusters in Mo, Hu, and Org_H_ labeled in the UMAP space. **b**, Corresponding landscape cell type specificity across conditions. **c**, Cross comparison of cell-type-specific expression patterns by landscape clusters across species (left) and contexts (right). Clusters with high divergence between conditions are highlighted in magenta. **d**, Early and late specific landscape clusters (top) and their distribution in the UMAP space (bottom). **e**, Corresponding landscape age specificity across conditions. Abbreviations: N, neuron; IP, intermediate progenitor; RG, radial glia; NonSpe, non-specific; AvgExprs, average expression.

**Extended Data Fig. 9:**
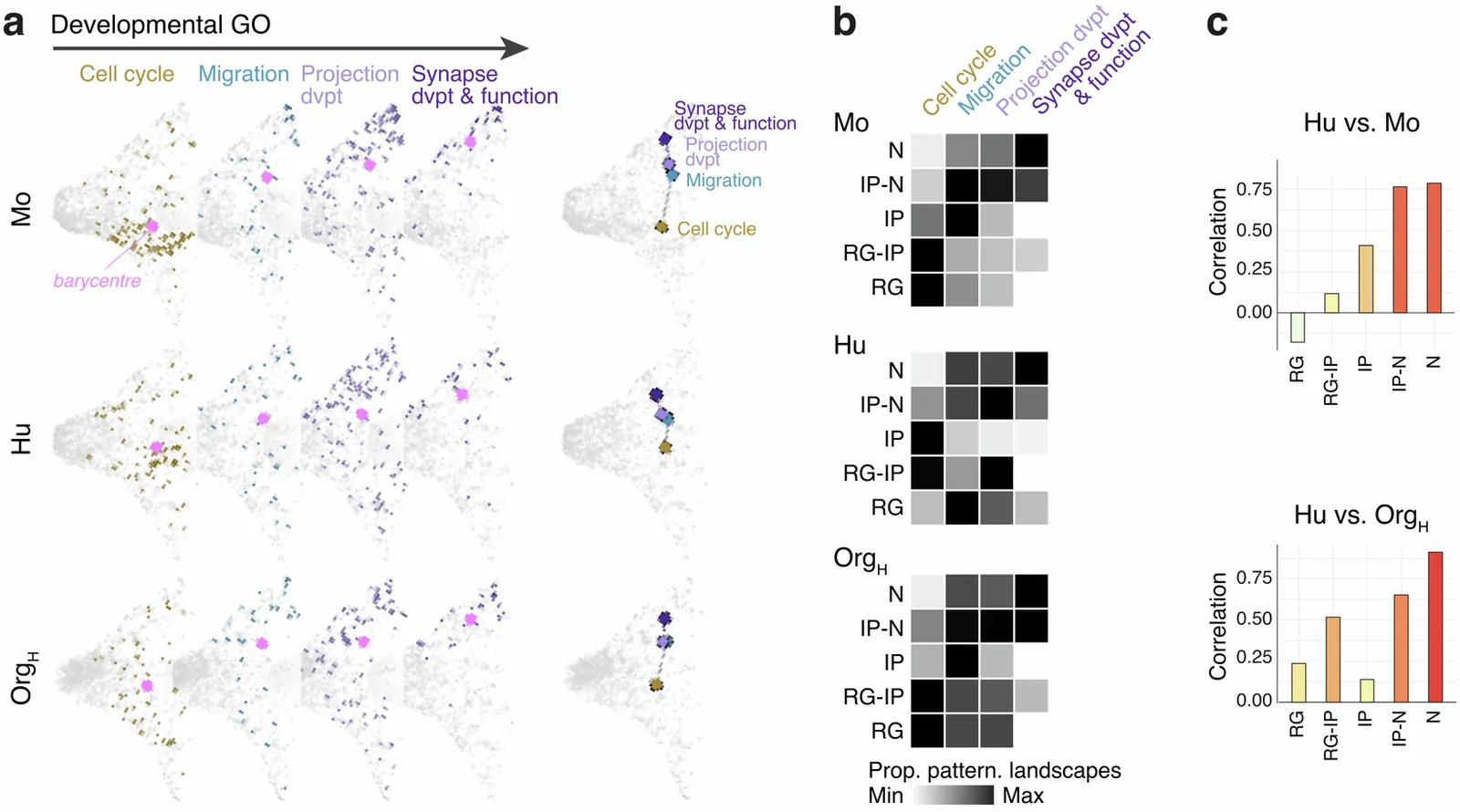
Cell-type-specific landscapes of genes associated with developmental ontologies. **a**, Landscapes of genes associated with cell cycle, migration, projection development and synapse development and function ontologies in Mo, Hu, and Org_H_ (left), and their barycenter for each ontology (right). **b**, Landscapes cell type specificity across developmental gene ontologies in Mo, Hu, and Org_H_. **c**, Correlations of gene ontologies for landscapes with corresponding cell type specificity between species (top) or contexts (bottom). Abbreviations: N, neuron; IP, intermediate progenitor; RG, radial glia; GO, gene ontology; dvpt, development.

Given their potential for biological interpretation, we focused our attention on genes with patterned landscapes. As a first approach to analyse their diversity, we applied a computer vision approach to extract 2,000 variable features of pixel distribution per landscape (see Methods). These features were used as parameters to display landscapes in a uniform manifold approximation and projection (UMAP) space (Fig. 2b, Extended Data Figs. 7c and 8 and Methods). The resulting visualization revealed 22 clusters of patterned landscapes, each representing distinct cyto-temporal patterns of gene expression (Fig. 2c). These clusters were organized along a differentiation axis, starting from landscapes with RG-specific expression (clusters 1 to 7) and ending with neuron-specific landscapes (clusters 18 to 22) in all three conditions (Fig. 2c, left, and Supplementary Table 3). Of note, very few genes showed cell-type-independent, age-specific expression (human: *n* = 135 genes with early cell-type non-specific expression and *n = *18 genes with late cell-type non-specific expression; mouse: *n* = 54 and 14 genes, respectively; Org_H_: *n* = 107 and 48 genes, respectively), suggesting a predominance of cell-type-specific, rather than strictly age-driven regulatory controls. Genes involved in sequential cellular processes during corticogenesis, such as genes with ontologies related to cell cycle, neuron migration, projection development and synapse development and function, were sequentially organized along this axis (Extended Data Fig. 9a and Methods). For example, in human, cluster 4 was enriched in genes involved in gliogenesis (for example, *BCAN* and *GFAP*), cluster 18 contained genes involved in dendrite morphogenesis and axonogenesis (for example, *CDK5* and *SEMA3A*) and cluster 22 contained genes involved in synapse organization (for example, *CAMK2B* and *FLRT2*) (Fig. 2c, right and Supplementary Table 3).

To understand how transcriptional programmes evolve during cortical development, we analysed gene expression patterns across the neurogenic lineage—from RG, via IPs, to neurons—and tracked their developmental dynamics. We defined five landscape categories on the basis of cellular expression: RG-only, RG and IP (RG-IP), IP-only, IP and N (IP-N), and N-only (Fig. 2d, top). The early progenitor (RG-only) and late neuronal (N-only) programmes were relatively conserved across species, suggesting fundamental constraints on stem cell maintenance and terminal differentiation. By contrast, the IP compartment showed clear evolutionary divergence. IP-only landscapes were substantially more frequent in human than in mouse, probably reflecting the expansion of the subventricular zone in primate cortical development (Fig. 2d, bottom, and Extended Data Fig. 8b). This expansion was accompanied by greater temporal diversity, including early IPs (human cluster 12; Fig. 2e and Extended Data Fig. 8c), which lacked a corresponding cluster in mouse, and may represent highly proliferative precursors driving emergence of the OSVZ^7^. Gene expression transitions also differed between mouse and human: approximately half of mouse RG-only genes shifted to RG-IP or IP-only in human, suggesting that genes originally restricted to RG have been co-opted into human IPs, potentially endowing them with enhanced self-renewal capacity (Extended Data Fig. 6k and Supplementary Table 3). OSVZ RG,  which are delaminated progenitors marked by *HOPX*, *FAM107A* and *TNC* expression^34,36^, progressively increased during human corticogenesis, paralleling OSVZ expansion^8^ (Extended Data Fig. 6). Accordingly, these RG markers belonged to cluster 4 in human, corresponding to late RG (Fig. 2c and Supplementary Table 3). Together, these data highlight evolutionarily distinct gene–cell relationships that may have contributed to cortical expansion through enhanced progenitor diversity and self-renewal in the subventricular compartment.

Many neurodevelopmental disorders affect the neocortex, yet how potentially involved genes relate to disease processes remains poorly understood. Using our dataset, we analysed 922 disease-associated genes expressed across 9 disorders, including cortical malformations (microcephaly, megalencephaly, lissencephaly, polymicrogyria and heterotopia), neuropsychiatric disorders (autism spectrum disorder and schizophrenia), epilepsy and Alzheimer’s disease. In humans, genes linked to cortical malformations were expressed predominantly in IPs, whereas those associated with neuropsychiatric disorders and epilepsy were enriched in neurons (Fig. 2f). This suggests distinct mechanisms at play: structural defects in cortical malformations arise from disruptions in neurogenesis or migration, whereas psychiatric disorders may involve pathways that affect later-stage circuit refinement. Notably, some Alzheimer’s disease-related genes (such as *APOE* and *LMNA*) were expressed in progenitors, suggesting possible developmental roots for this late-onset disorder^43^ and/or pleiotropic roles in both neurodevelopment and neurodegeneration. The resource (https://www.humous.org) can thus serve to stratify patients not only on affected genes but on the—arguably functionally relevant—cyto-temporal expression patterns of these genes.

Expression comparison of genes defined by their developmental ontologies revealed distinct organizational principles across species. In mouse, developmental genes follow a strict sequential expression pattern: cell cycle genes are expressed in RG-IP, followed by migration genes in IP-N, and finally projection or synapse genes in neurons. By contrast, in human there are more overlapping patterns, with IPs maintaining expression of cell cycle genes while RG simultaneously express migration and projection-related genes (Extended Data Fig. 9a,b). Additionally, correlation analysis revealed that the degree of ontology conservation between species varies by cell type: ontologies of genes expressed in progenitors (RG and early IPs) showed low correlation, whereas ontologies of genes expressed in neurons showed higher correlation (Extended Data Fig. 9c). Thus human cortical cells display overlapping rather than strictly sequential developmental programmes compared with mouse, with divergence mainly in progenitors and higher conservation in neurons.

**Extended Data Fig. 10:**
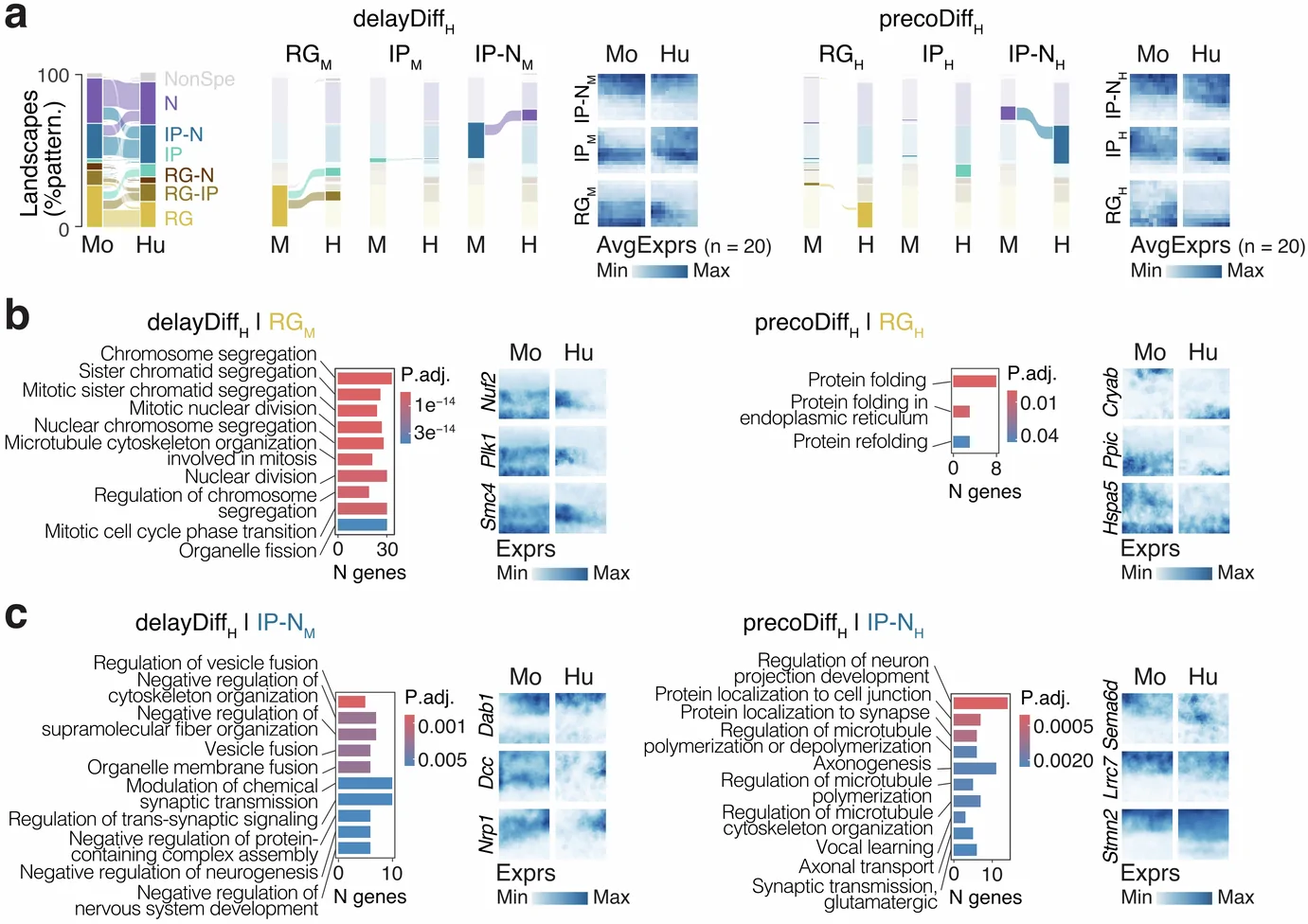
Delayed and precocious human gene expression profiles along differentiation axis. **a**, Left: Corresponding landscape cell type specificity between species. Middle: Definition of landscapes with delayed differentiation specificity in Hu (delayDiff_H_) and their average expression (AvgExprs) in Mo and Hu. Right: Definition of landscapes with precocious differentiation specificity in Hu (precoDiff_H_) and their average expression in Mo and Hu. **b**, Left: Top 10 ontologies of delayDiff_H_ genes expressed in Mo RG. Right: Top 10 ontologies of precoDiff_H_ genes expressed in Hu RG. **c**, Left: Top 10 ontologies of delayDiff_H_ genes expressed in Mo IP-N. Right: Top 10 ontologies of precoDiff_H_ genes expressed in Hu IP-N. In b-c, Examples of gene expression landscapes related to the ontologies are shown in Mo and Hu. Gene Ontology over-representation analysis for biological processes was performed using the enrichGO function from the clusterProfiler package. Enrichment was tested using a one-sided hypergeometric test, and *P* values were adjusted for multiple comparisons using the Benjamini–Hochberg FDR method. Terms with adjusted *P* < 0.05 were considered significant. Only significant gene ontologies are shown. Abbreviations: N, neuron; IP, intermediate progenitor; RG, radial glia.

We next compared gene expression dynamics along the differentiation axis between species by classifying landscapes on the basis of relative cyto-temporal expression. This revealed sets of genes with either delayed (*n* = 288) or precocious (*n* = 176) differentiation stage expression in human versus mouse (Extended Data Fig. 10a). In RG, delayed human genes were enriched for cell division ontologies (for example, *NUF2* and *PLK1*), whereas precocious ones were related to protein folding (for example, *CRYAB* and *PPIC*). In IP-N, genes that were delayed in human were linked to synapse function (for example, *NRP1*), while precocious ones related to axon development and cytoskeleton organization (for example, *SEMA6D* and *STMN2*) (Extended Data Fig. 10b,c and Supplementary Table 4). Earlier axon development together with delayed synaptic maturation in humans may reflect that basic wiring is established early, but is later refined by environmental signals over a prolonged developmental period, consistent with previous interspecies comparisons^14,26,33,44^.

**Fig. 3:**
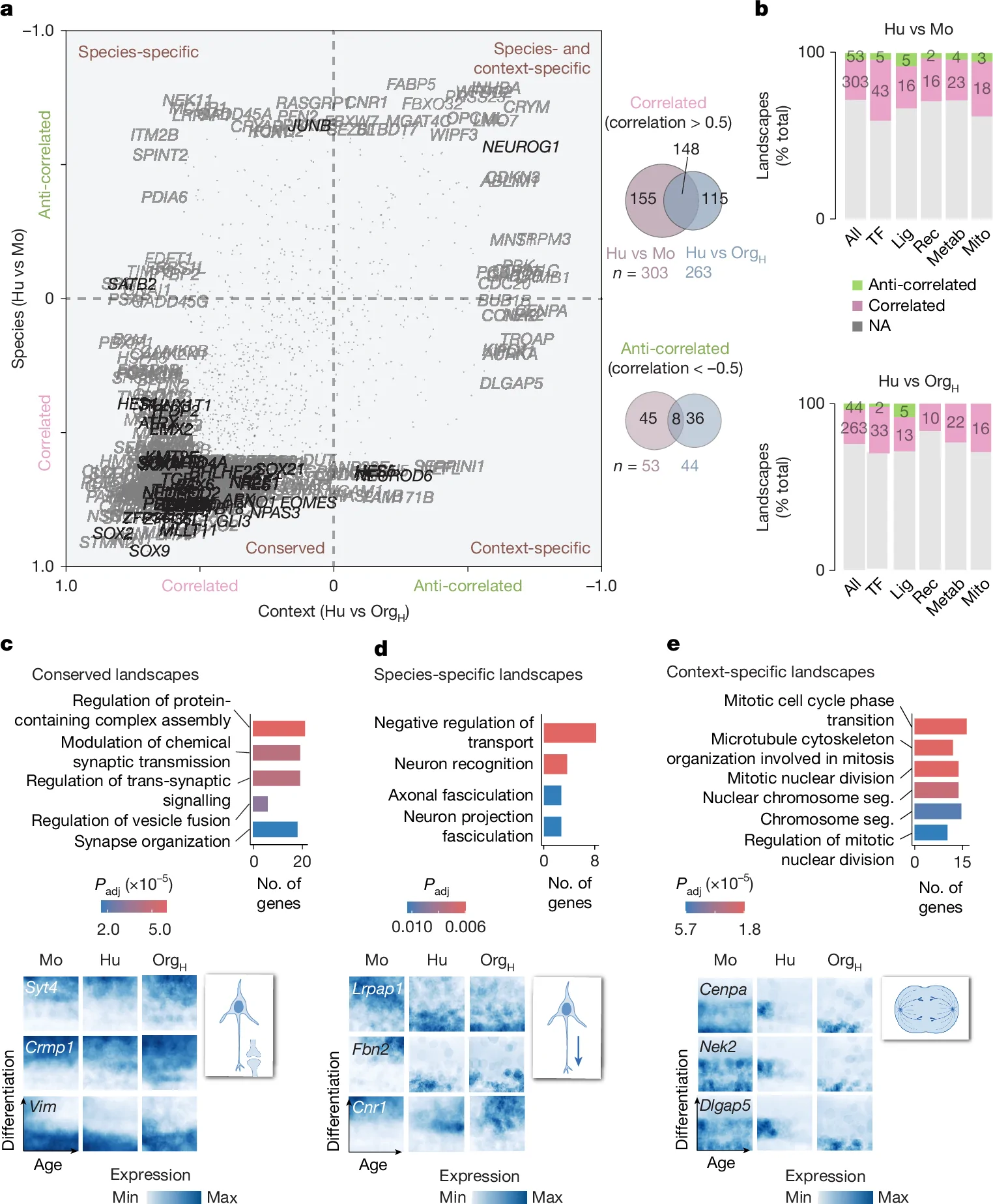
Identification of conserved and divergent gene expression programmes across species and context. **a**, Left, pairwise correlation analysis of gene expression landscapes across species (human versus mouse; *y* axis) and context (human versus Org_H_; *x* axis). Transcription factors labels are in black font. Right, overlap in correlated (top) and anti-correlated (bottom) landscapes in species and context comparisons. **b**, Distribution of correlated and anti-correlated landscapes between species (top) and context (bottom) for select gene classes. Lig, ligands; Metab, metabolic; Mito, mitochondrial; NA, not assigned; Rec, receptors; TF, transcription factors. **c**–**e**, Representative landscapes and enriched biological processes for conserved (**c**; for example, *SYT4*), species-specific (**d**; for example,* CNR1*) and context-specific (**e**; for example,* CENPA*) genes. Enrichment was tested using a one-sided hypergeometric test, and *P* values were adjusted for multiple comparisons using the Benjamini–Hochberg false discovery rate method. Terms with adjusted *P* < 0.05 were considered significant. Seg., segregation. Adapted from BioRender; Pravata, M. V. https://BioRender.com/d8nicg0 (2026).

**Extended Data Fig. 11:**
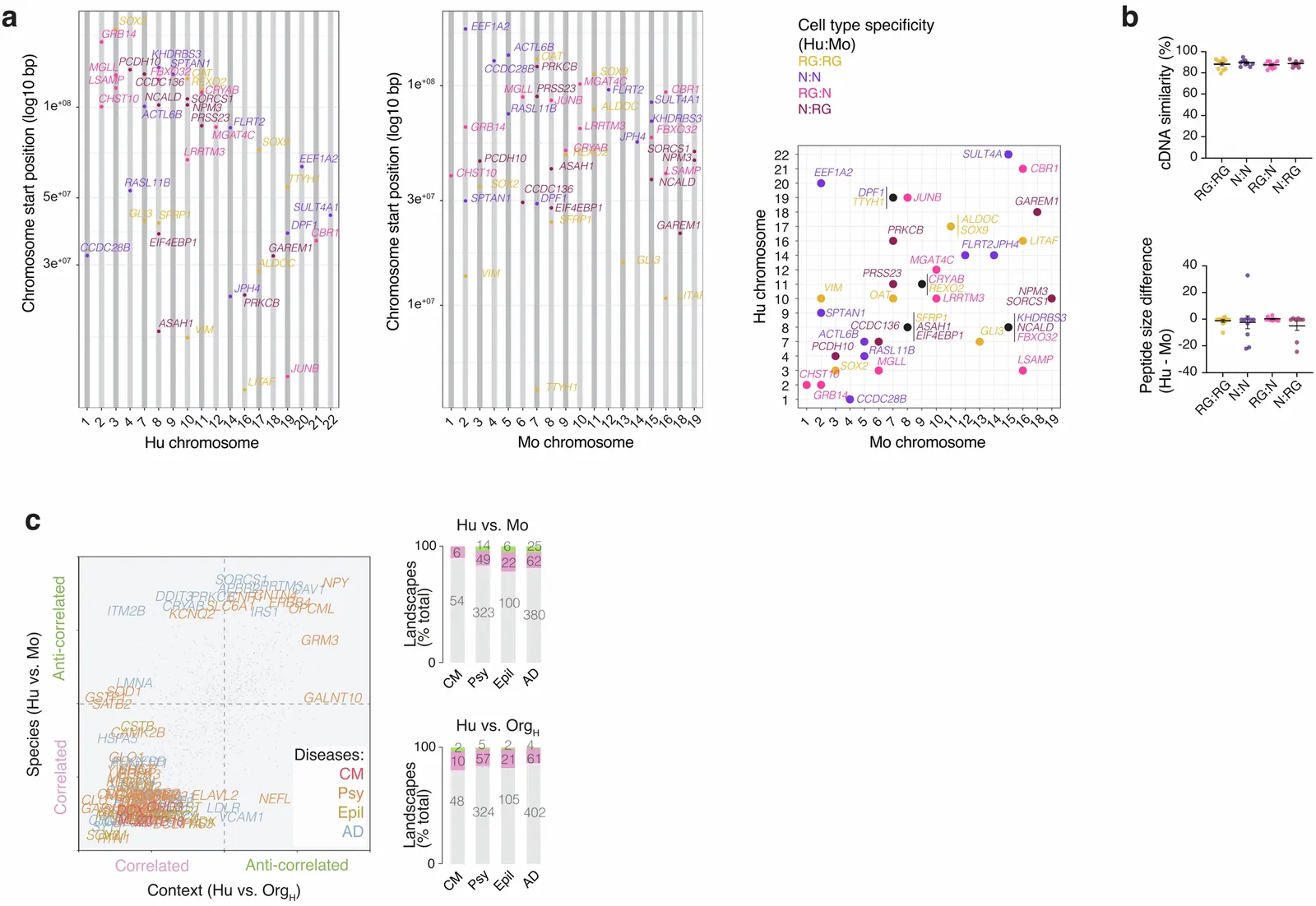
Genes with convergent or divergent expression landscapes between species and contexts. **a**, Hu (left) and Mo (middle) chromosome position of genes expressed in RG (yellow) or N (purple) in both Mo and Hu (convergent expression), genes expressed in Mo RG and Hu N (brown), and genes expressed in Mo N and Hu RG (magenta) (divergent expression). Right: Comparison of chromosome positions in Hu and Mo. **b**, Hu and Mo cDNA similarity and encoded peptide size difference of genes with convergent and divergent expression. Each dot represents a gene shown in **a**. Data are presented as mean values +/− SEM. **c**, Left: Pairwise correlations of disease-associated gene expression landscapes across species and contexts. CM includes microcephaly, megalencephaly, lissencephaly, polymicrogyria and heterotopia; Psy includes autism spectrum disorder and schizophrenia. Right: Distribution of correlated and anti-correlated landscapes between species (top) and contexts (bottom) for disease-associated genes. Abbreviations: Mo, mouse; Hu, human; Org_H_, human-derived cortical organoids; P.adj, adjusted *P *value; Diff, differentiation; Exprs, normalized expression; CM, cortical malformation; Psy, neuropsychiatric diseases; Epil, epilepsy; AD, Alzheimer’s disease.

**Extended Data Fig. 12:**
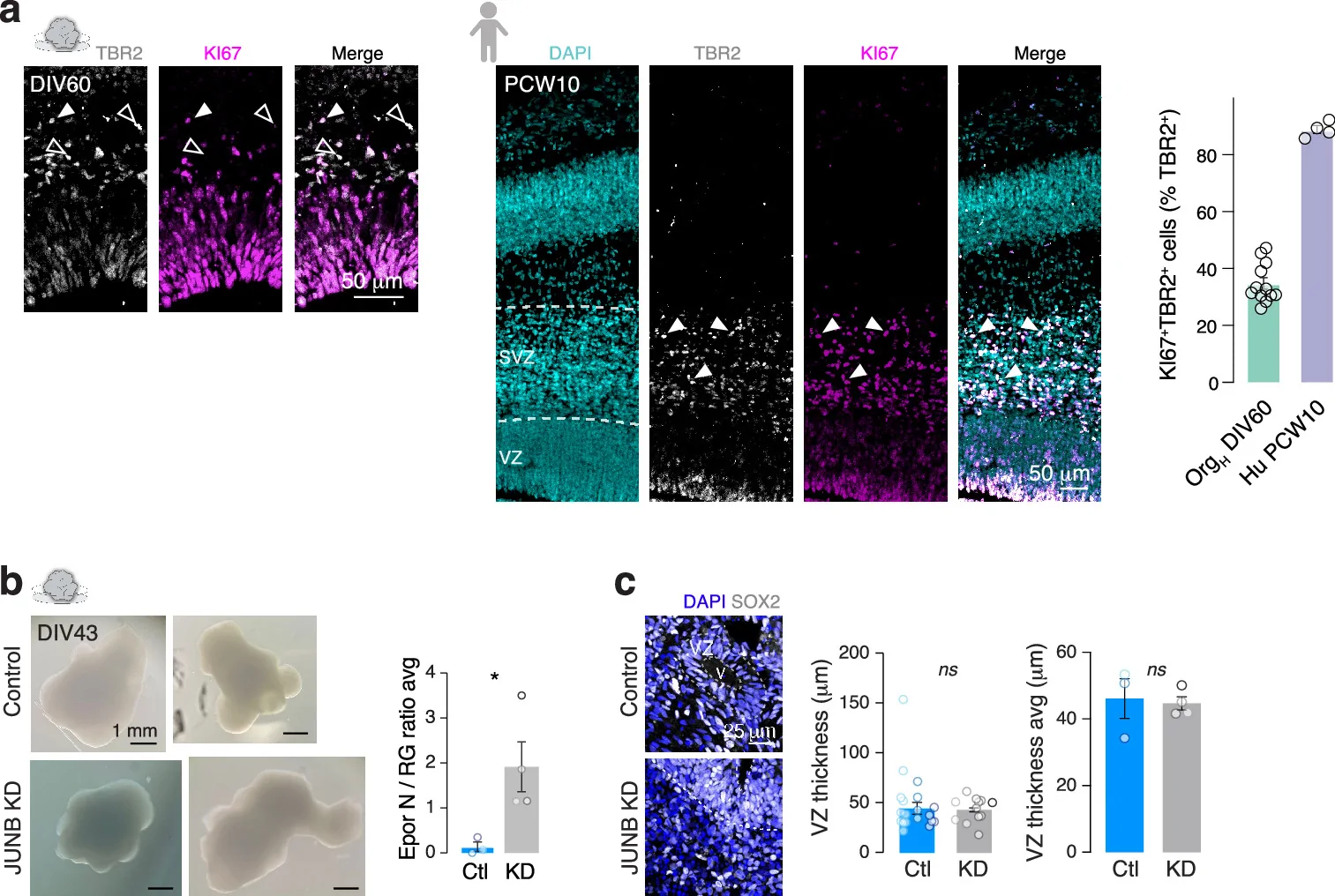
Comparison of early IP proliferative capacity between contexts and absence of morphological changes in Org_H_ upon electroporation. **a**, Photomicrographs representative of DIV60 Org_H_ (left) and PCW10 Hu fetal cortex (middle) stained for IP and proliferative markers, TBR2 and KI67 respectively. Empty arrowheads: TBR2^+^/KI67^−^ cells; full arrowheads: TBR2^+^/KI67^+^ cells Right: quantifications of proliferative IP in the 3 conditions. *n* = 12 ventricles analyzed in *n* = 3 Org_H_; *n* = 4 Hu sections from one individual. Dots on the graph represent Org_H_ ventricles and Hu sections. **b**, Photomicrographs representative of the human-derived electroporated Org_H_ (left) and Epor N / RG ratio average per organoid. Each dot represents an independent organoid (*n* = 3 Ctl; *n* = 4 knockdown (KD)). Mean N / RG ratio per organoid was compared between groups using a two-tailed unpaired t-test with Welch’s correction (*P* = 0.0466). Shades of colors are the same as in panel **c** and Fig. 4c. **c**, Photomicrographs illustrating how ventricles and VZ are defined in Org_H_ based on SOX2 and DAPI stainings (left) and measure of VZ thickness in Ctl and JUNB KD conditions per ventricle (middle) and per organoid (right). Middle: Each dot represents a ventricle (*n* = 19 Ctl; *n* = 12 KD). Average VZ thickness was analyzed using two-way ANOVA (α = 0.05); no significant effects were detected (row = organoid: *P* = 0.7045; column = conditions: *P* = 0.9327). Right: Each dot represents an independent organoid (*n* = 3 Ctl; *n* = 4 KD). Mean VZ thickness per organoid was compared between groups using a two-tailed unpaired t-test with Welch’s correction; *P* = 0.8343). Shades of colors representing each organoid are the same as in panel **b** and Fig. 4c. Data are presented as mean values ± s.e.m (standard error mean) (**a**–**c**); *P < 0.05. Abbreviations: DIV, days in vitro; PCW, postconception week; Ctl, control; VZ, ventricular zone; SVZ, subventricular zone; ns, not significant.

To identify genes whose expression diverges most across species and context, we next measured pairwise correlation values between landscapes patterned in all conditions (that is, mouse, human and Org_H_; Fig. 3a and Methods). This analysis identified four classes of landscapes: (1) conserved—that is, showing high correlation for all comparisons; (2) species-specific—that is, differing between mouse and human but conserved between human and Org_H_; (3) context-specific—that is, differing between human and Org_H_ but conserved across species, potentially highlighting environment-sensitive genes; and (4) divergent—that is, showing low correlation for all comparisons. Genes with conserved expression patterns formed the largest category (26%), while only 8% were conext-specific (Fig. 3a), indicating broad conservation of gene regulatory programmes. Divergent expression patterns were not explained by basic genomic or protein features, including chromosomal location, coding sequence conservation, peptide length and functional class (Fig. 3b and Extended Data Fig. 11a,b). Instead, these patterns reflected specific biological functions; for example, conserved landscapes were enriched for genes involved in fundamental neuronal processes, such as synaptic function (for example, *SYT4*, a key regulator of neurotransmitter release). Species-specific landscapes were enriched for genes that control more specialized features, such as axon fasciculation (for example, *CNR1*, which guides axon organization), whereas context-specific landscapes showed enrichment for cell division genes (for example, *CENPA*, essential for chromosome segregation) (Fig. 3c–e and Supplementary Table 5). At early stages of corticogenesis, cell division genes were expressed specifically in human IPs; these cells formed a specific cluster (trans-IP) with transitional identity between RG and IP, consistent with previous findings^45^, and which we identified as the most divergent from their mouse or Org_H_ counterparts (Fig. 2 and Extended Data Fig. 6a–d, and cluster 12 in Extended Data Fig. 8c). These human early IP-specific programmes could contribute to their higher proliferation rate, which has been reported in human but not in mouse^7^. The proliferative property of human early IPs was not reproduced in vitro in 2-month Org_H_, in which less than 40% of IPs expressed the proliferation marker Ki-67, compared with 80% in PCW10 human fetal cortex, suggesting that microenvironmental signals may be important in setting IP proliferative potential (Extended Data Fig. 12a).

Finally, cross-condition correlations revealed that disease-associated genes show conservation patterns resembling those of other genes (Extended Data Fig. 11c), suggesting that pathogenicity is not primarily linked to evolutionary divergence. Together, these findings demonstrate that most gene expression programmes are preserved across species and context, with some being specific to either species or environmental conditions.

**Fig. 4:**
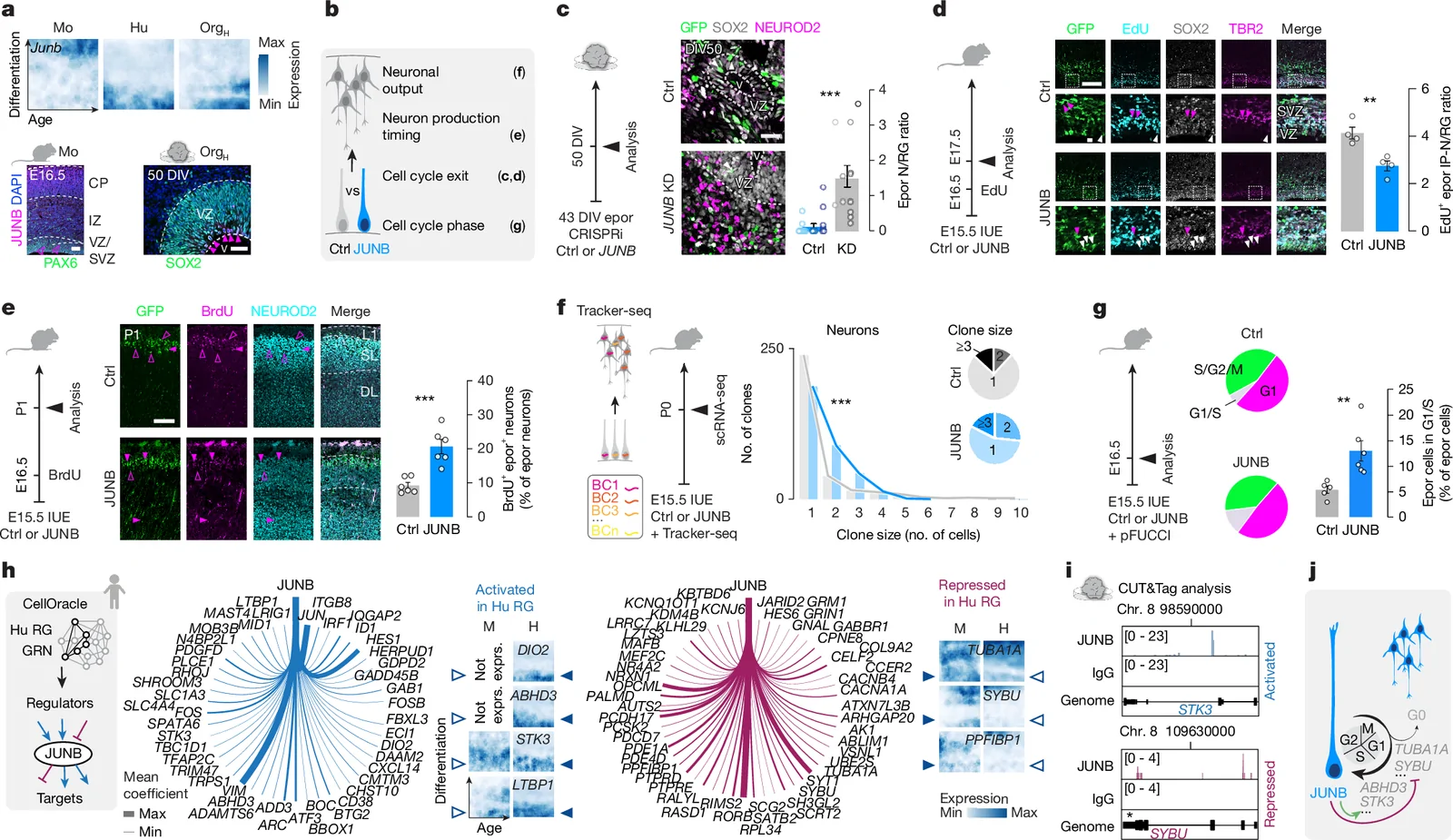
JUNB bidirectionally controls species-specific cortical progenitor properties. **a**, Top, JUNB expression landscapes. Bottom, JUNB immunostaining in Org_H_ and mouse VZ (repeated in two independent experiments). Filled and empty arrowheads indicate JUNB^+^ and JUNB^−^ progenitors, respectively. Scale bars: 50 μm (left), 25 μm (right). DIV, days in vitro; IZ, intermediate zone. **b**, Schematic overview of analyses. Ctrl, control. **c**, *JUNB* knockdown (KD) in Org_H_ and neuron-to-progenitor ratio. White arrowheads, electroporated progenitors (SOX2^+^); magenta arrowheads, neurons (NEUROD2^+^; empty arrowheads non-electroporated; filled arrowheads, electroporated). Each dot represents one ventricle; position and colour shade indicate organoid of origin (*n* = 3 control and 4 *JUNB*-knockdown organoids; see Extended Data Fig. 12b). Two-way ANOVA (*α* = 0.05). Epor, electroporation. **d**, Left, experimental design. Middle, representative photomicrographs. Right, quantification of E16-born electroporated IP-N/RG ratio. Arrowheads, RG (EdU^+^SOX2^+^TBR2^−^, white) and IPs (EdU^+^ SOX2^−^TBR2^+^, magenta). IUE, in utero electroporation. Ctrl: *n* = 4, JUNB: *n* = 4. Two-tailed Student’s *t*-test (*P* = 0.0055). Scale bars: 100 μm (main images), 50 μm (expanded regions). **e**, Left, experimental design. Middle, representative photomicrographs. Right, quantification of E16-born electroporated neurons. Ctrl: *n* = 6, JUNB: *n* = 6. Two-tailed Student’s *t*-test (*P* = 0.0006). P1, postnatal day 1. DL, deep layers; SL, superficial layers. Scale bar, 100 μm. **f**, Left, Tracker-seq principle and experimental design. Right, analysis of neuron number per clone from control or JUNB-expressing RG. Pie charts show the clone size distribution. BC, barcode. *χ*^2^ test (*χ*^2^ = 51.432, *P* = 5.788 × 10^−8^). **g**, Left, experimental design. Middle, pie charts of electroporated RG cell cycle phases. Right, quantification of G1/S phase proportions. Ctrl: *n* = 5, JUNB: *n* = 6. Two-tailed Student’s *t*-test (*P* = 0.0093). **h**, CellOracle analysis workflow identifying targets of JUNB and regulators (Fig. 5). Chord diagrams show predicted activated (left) and repressed (right) targets that are expressed in RG versus RG-IP and IP versus IP-N. Line width represents mean coefficient score. Insets, representative gene expression landscapes (filled arrowheads, expression; empty arrowheads, no expression). **i**, JUNB and IgG CUT&Tag at representative loci. Asterisk indicates promoter. **j**, Model of JUNB regulation of cell cycle progression. Data are mean ± s.e.m. In **c**–**e**,**g**, dots represent individual biological replicates. Data in **c** were generated from multiple organoid batches, whereas data in **i** were generated from only one batch of organoids. Data in **d** were generated from one mouse litter, whereas data in **e**–**g** were generated from multiple litters. Schematics and illustrations adapted from BioRender; Pravata, M. V. https://BioRender.com/spvnnlp (2026). Source data

**Extended Data Fig. 13:**
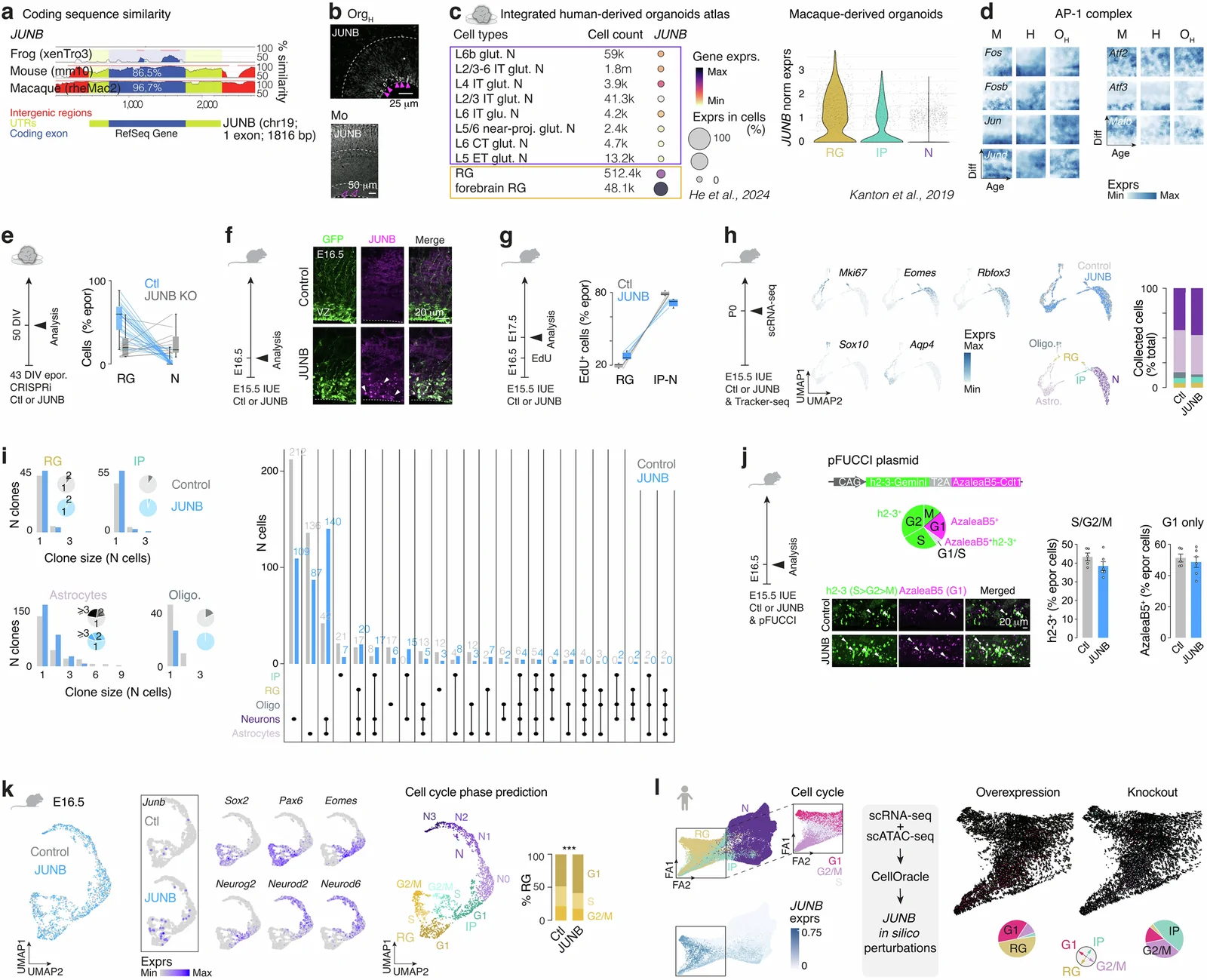
JUNB expression changes mouse radial glia properties and neuronal output. **a**, Similarity in *JUNB* coding exon, UTRs and intergenic regions in Frog, Mouse, and Macaque compared to Human. Data are from the rVista2.098. **b**, Photomicrographs showing JUNB immunostaining (from Fig. 4a) in day 50 Org_H_ (top) and E16 Mo cortical samples (bottom), repeated in two independent experiments. **c**, Expression of *JUNB* in human-derived (from ref. ^29^; left) and macaque-derived (from ref. ^33^; right) organoids scRNA-seq data. Cell type annotations are from the original publications. **d**, Expression landscapes of genes coding for other AP-1 complex members in Mo, Hu, and Org_H_. **e**, Schematic representing the design of CRISPRi-mediated JUNB knockdown in Org_H_ and raw neuron-to-progenitor ratio of data presented in Fig. 4c. **f**, Schematic showing the experimental design for JUNB overexpression in Mo cortex and photomicrographs demonstrating JUNB protein in Mo VZ electroporated cells 24 h after *in utero* electroporation with pCS2::JUNB plasmid (right). White arrows indicate GFP^+^JUNB^+^ cells. **g**, Schematic illustrating the experimental design to assess extension of neurogenic window in Mo and raw proportions of electroporated cells labelled by EdU between Ctl and JUNB from data presented in Fig. 4d. **h**, Tracker-seq experiment. Left: Feature plots with marker genes and cell type definition in the UMAP space. Right: Proportion of cells in control and JUNB conditions. **i**, Left: Clone size by cell types. Right: Number of cells per clone composition found in the Tracker-seq data. **j**, Left: Experimental design showing pFUCCI analysis. Center: pFUCCI plasmid schematic along with a pie chart showing the distribution of flourophores in specific cell cycle phases (top) and images demonstrating labelled cells by pFUCCI (bottom). White arrows show h2-3^+^AzaleaB5^+^ cells. Right: Quantifications of electroporated RG in S/G2/M or G1-only phases using pFUCCI. Each dot represents biological replicates for Ctl (*n* = 5) and JUNB (*n* = 6). Data are presented as mean values +/− SEM. **k**, Single-nucleus RNA-seq data of Ctl and JUNB *in utero* electroporated cells at E15.5, collected at E16.5. Left: Control and JUNB cells in the UMAP space. Center left: Junb expression in control and JUNB conditions. Center right: marker genes used for cell type annotations. Right: Cell cycle phase prediction by cell type and proportions of RG in G1, S and G2/M phase in Ctl and JUNB conditions. Differences in the distribution of cells across cell cycle phases between conditions were assessed using a two-sided Fisher’s exact test implemented in R (***P < 0.001). **l**, Left: RG cell cycle phase annotation (top) and expression of JUNB (bottom) in Hu single-cell multiome data. Right: Prediction of Hu single cell trajectories upon JUNB overexpression or knockout using CellOracle in silico analysis. Pie charts representing the proportions of trajectories towards each cardinal direction (*i.e*., G1, G2M, RG, IP). Abbreviations: Ctl, control; N, neuron; IP, intermediate progenitor; RG, radial glia. Exprs, expression; oe, overexpression; ac, acetylation.

Genes that have anti-correlated expression patterns are particularly interesting because they offer an opportunity to study how evolutionary changes in gene regulation can drive species-specific features. Among anti-correlated genes in vivo, *JUNB*, a component of the AP-1 transcription factor complex that regulates cellular responses to growth and stress signals^46^, was the transcription factor gene with the most anti-correlated expression pattern (correlation = −0.64; Fig. 3a). *JUNB* is composed of one exon with more than 85% similarity between human and mouse (and more than  95% similarity between human and macaque; Extended Data Fig. 13a). In mouse, *Junb* is expressed in neurons, whereas in human and Org_H_, it is mostly confined to RG (Fig. 4a). Immunostaining confirmed this cell-type specificity, with protein expression restricted to juxtaventricular progenitors in Org_H_ VZ and no expression in mouse VZ (Fig. 4a and Extended Data Fig. 13b). In macaque-derived cortical organoids, *JUNB* is also enriched in RG and absent in neurons^29,33^ (Extended Data Fig. 13c). This species-specific pattern is in contrast to genes encoding other members of the AP-1 complex, whose cyto-temporal expression is mostly conserved (Extended Data Fig. 13d). Given this largely mutually exclusive pattern of expression between mouse and human, we investigated a potential species-specific function of JUNB during corticogenesis (Fig. 4b).

## Function and targets of JUNB in RG

To study the functional properties that JUNB imparts to human RG, we performed gain- and loss-of-function experiments. *JUNB* knockdown using CRISPR interference (CRISPRi) in Org_H_ led to an increase in neuron-to-progenitor ratio, consistent with accelerated neurogenesis (Fig. 4c and Extended Data Figs. 12b,c and 13e). Accordingly, JUNB overexpression in mouse RG using in utero electroporation at E15.5 increased RG numbers at the expense of IP and N numbers (that is, decreased IP-N/RG ratios; Fig. 4d and Extended Data Fig. 13f,g). Similarly, neuronal generation was extended, as demonstrated by a higher fraction of neurons born after the first 24 h after electroporation (Fig. 4e). JUNB-induced extended RG self-replication ultimately led to an increase in neuronal production, as shown by clonal analysis using Tracker-seq (Fig. 4f, Extended Data Fig. 13h,i and Methods). Thus JUNB is necessary and sufficient to promote the extended proliferative capacity characteristic of human RG. Consistent with a decrease in G0 differentiative fate choice, JUNB increased the fraction of RG in G1/S phase, as shown using the cell cycle reporter pFUCCI in mouse (Fig. 4g and Methods), scRNA-seq 24 h after in utero electroporation, and in silico perturbations of *JUNB* in human RG using CellOracle (Extended Data Fig. 13j–l and Methods).

**Extended Data Fig. 14:**
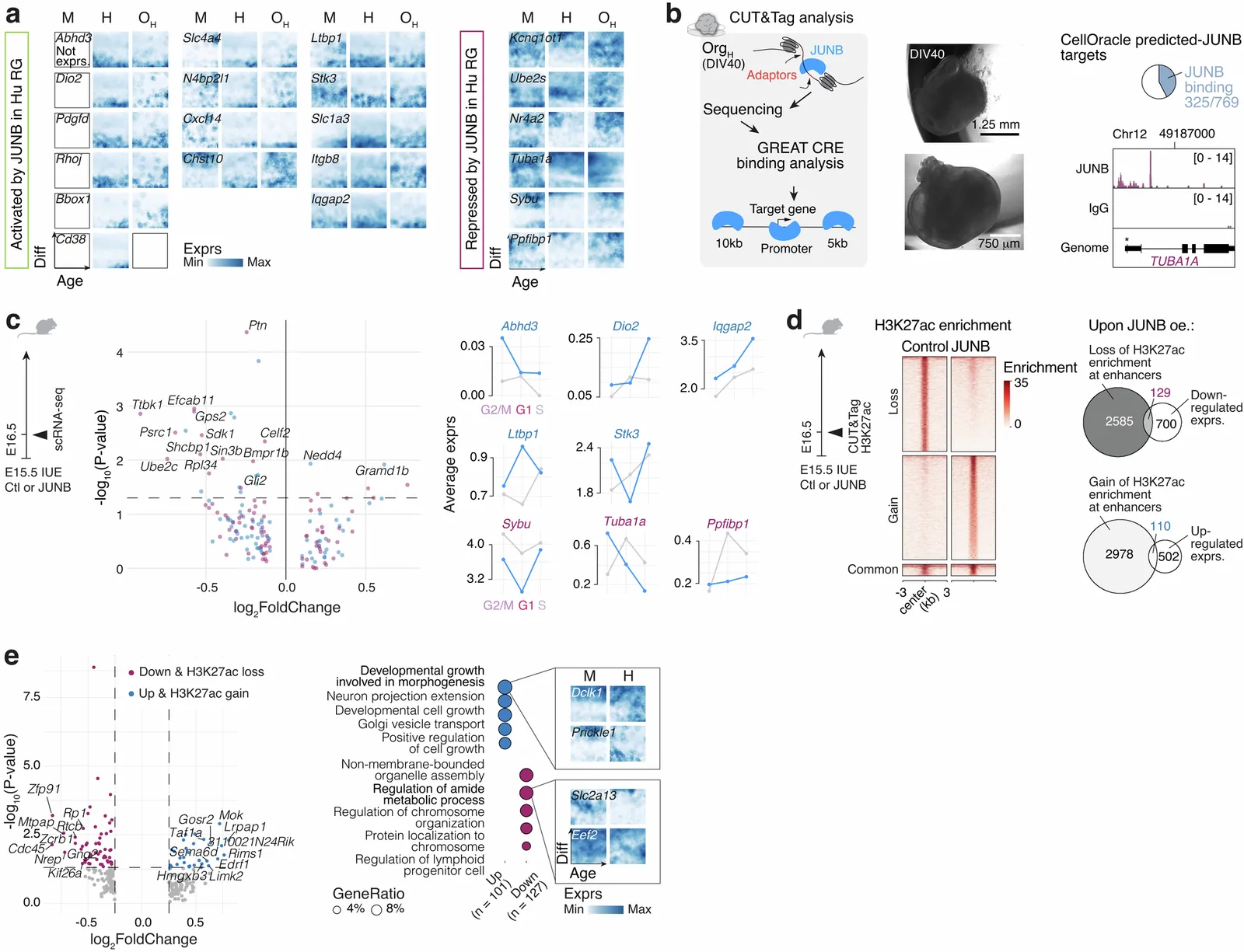
JUNB-induced gene expression changes in Hu and Mo. **a**, Expression landscapes of genes activated (left) or repressed (right) by JUNB in Hu RG. **b**, Left: CUT&Tag analysis principle. Center: Images of the DIV40 Org_H_ used for CUT&RUN or CUT&Tag analyses. Right: CellOracle predicted JUNB targets showing direct binding with JUNB (top), and example of binding for a repressed target (bottom). Asterisk shows the transcription start site. **c**, CellOracle predicted Hu targets differentially expressed in Mo RG upon JUNB overexpression using snRNA-seq. Left: volcano plot (dashed line shows *P* = 0.05). Right: expression of activated and repressed Hu JUNB targets (normally not expressed or more expressed in Mo RG, respectively) in JUNB overexpressing Mo RG. **d**, Left: H3K27 acetylation enrichment analysis using CUT&Tag in Control or JUNB overexpressed Mo cortical samples. Right: Comparison of loss/gain of H3K27ac and expression changes in RG from snRNA-seq data. **e**, Left: Genes down- or up-regulated with H3K27ac loss or gain, respectively, upon JUNB overexpression in Mo. Right: Corresponding gene ontologies with examples of expression landscapes for select genes. Differential gene expression between conditions was performed using the FindMarkers function in Seurat (min.pct = 0.2). Statistical significance for Gene Ontology (GO) enrichment was assessed using a hypergeometric test, and *P* values were adjusted for multiple comparisons using the Benjamini–Hochberg FDR method (**c**,**e**). Abbreviations: Ctl, control; N, neuron; IP, intermediate progenitor; RG, radial glia. Exprs, expression; oe, overexpression; ac, acetylation.

This in silico analysis identified 769 putative JUNB target genes in human RG, with roughly equal proportions of activated and inhibited genes (Supplementary Table 6). Among predicted activated targets, approximately 10% (32 genes) showed a JUNB-like expression pattern (RG-enriched) (Fig. 4h, Extended Data Fig. 14a and Supplementary Table 6). CUT&Tag profiling of JUNB-binding sites on DNA revealed that about half of these genes (17 out of 32) were direct targets for JUNB, with some (7 out of 32) showing consistent acetylation enrichment and gene expression in mouse after JUNB overexpression (Fig. 4i, Extended Data Fig. 14b–e and Supplementary Table 6). Thus, JUNB not only binds to DNA but is able to remodel the chromatin landscape through histone acetylation in mouse RG. Of note, about 80% (27 out of 32) of these genes have known functions in cell cycle control (Supplementary Table 6), providing a mechanism through which JUNB can regulate human-typical progenitor proliferation and extend neurogenic output (Fig. 4j).

## Upstream regulation of JUNB expression

**Fig. 5:**
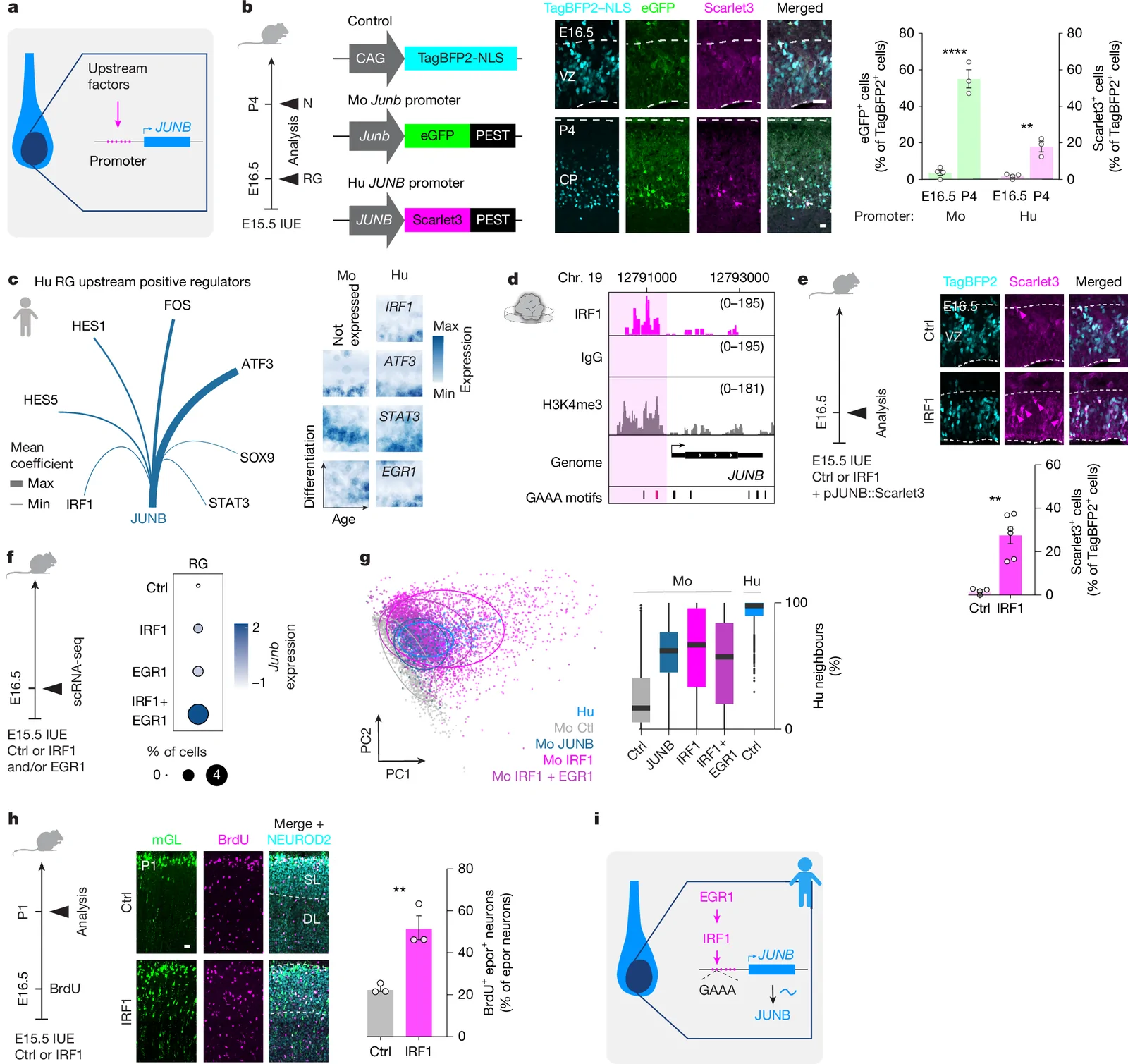
Availability of the upstream factor IRF1 regulates *JUNB* expression in human RG. **a**, Schematic overview of upstream factor and promoter analyses (see also Extended Data Fig. 15). **b**, Left, mouse and human *JUNB* promoter constructs driving destabilized eGFP (mouse) or Scarlet3 (human) expression. CAG-TagBFP2-NLS served as electroporation control. Middle, representative photomicrographs after electroporation in mouse cortex (E16.5 or P4). Bottom, proportions of TagBFP2–NLS^+^ cells expressing reporters. Each dot represents one biological replicate (mouse: *n* = 4; human: *n* = 3). Unpaired two-tailed Student’s *t*-test (mouse: *P* = 0.00008; human: *P* = 0.0014). Scale bars, 10 μm. **c**, Left, chord diagram of predicted upstream regulators of JUNB expressed in RG versus RG-IP. Line width represents mean coefficient score. Right, representative landscapes in mouse and human. **d**, CUT&RUN profiles for IRF1, H3K4me3 and IgG at the *JUNB* locus. IRF1 binding motifs (GAAA) are indicated, with the mutated site highlighted in magenta. Data were generated from two pooled organoids of the same batch. **e**, Left, promoter trap design with human IRF1. Middle, representative photomicrographs (control or IRF1 overexpression). Arrowheads indicate reporter activation. Right, quantification of TagBFP2–NLS^+^Scarlet3^+^ cells. Each dot represents one biological replicate (control: *n* = 4; IRF1: *n* = 6). One-way ANOVA with Tukey correction (*P* = 0.0027). Scale bar, 10 μm. **f**, Left, design of scRNA-seq experiment following overexpression of IRF1 and/or EGR1 in mouse. Right, proportions of *Junb*-expressing cells across conditions. **g**, Left, principal component analysis of integrated RG from human control (PCW15–24), mouse control and mouse overexpression conditions (JUNB, IRF1, or IRF1 plus EGR1). Right, neighbour analysis. Mouse control: *n* = 1,485; JUNB: *n* = 328; IRF1: *n* = 2,214; IRF1 plus EGR1: *n* = 1,780; human control: *n* = 1,960 cells. Boxes show median distribution and error bars represent cell spread. **h**, Left, design of birthdating experiment (control and IRF1). Middle, representative photomicrographs (E16.5 BrdU pulse). Right, proportions of GFP^+^BrdU^+^NEUROD2^+^ cells. Each dot represents one biological replicate (*n* = 3 per condition). Unpaired two-tailed Student’s *t*-test (*P* = 0.0077). Scale bar, 10 μm. **i**, Model of *JUNB* regulation by EGR1 and IRF1 in human RG. In **b**,**e**,**h**, data are mean ± s.e.m. Data in **b**,**e**,**f**,**g** were generated from multiple mouse litter, whereas data in **h** were generated from one litter. Schematics and illustrations adapted from BioRender; Pravata, M. V. https://BioRender.com/spvnnlp (2026). Source data

**Extended Data Fig. 15:**
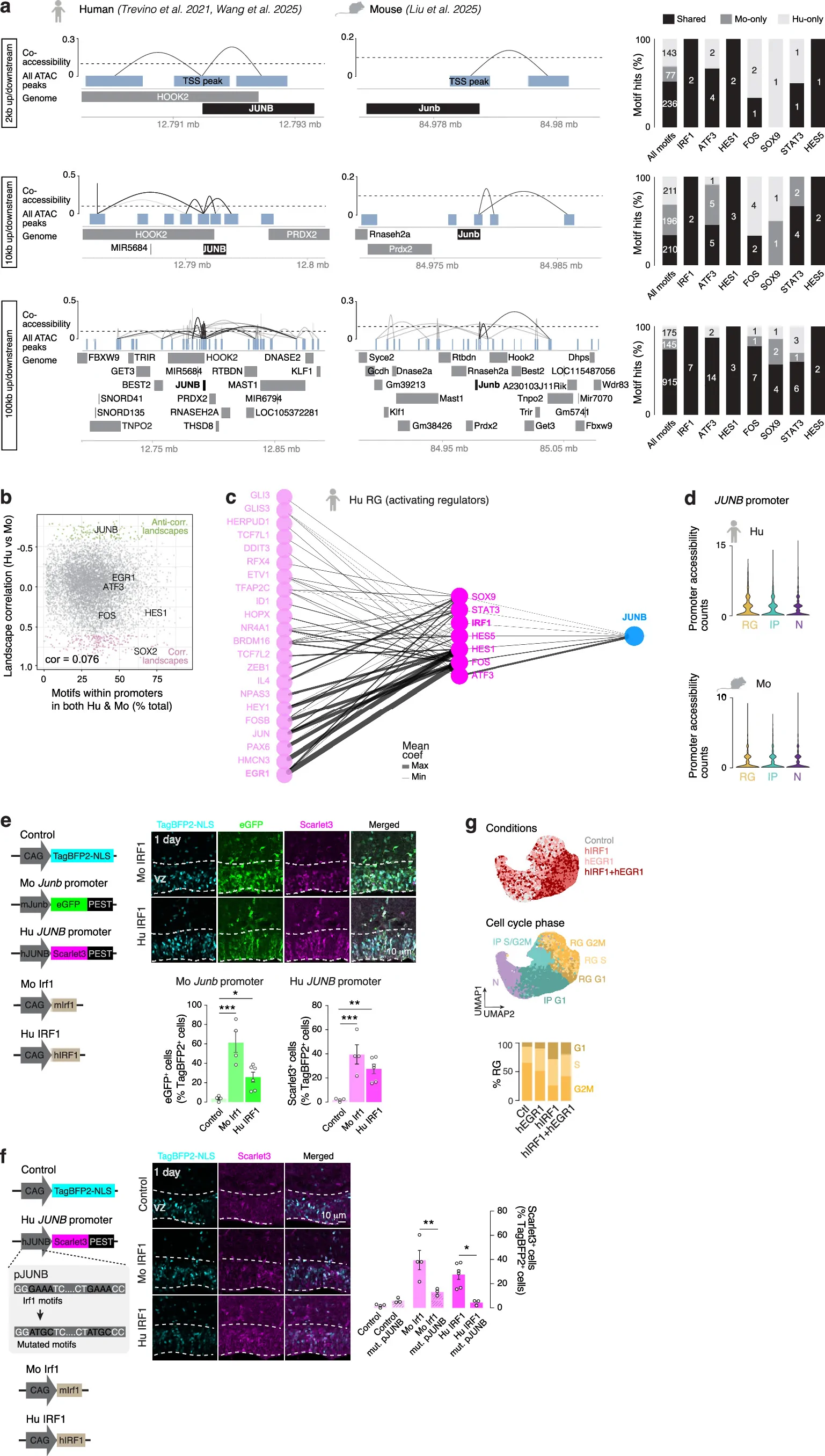
JUNB regulatory sequences comparison across species. **a**, Cicero co-accessibility analysis of ATAC peaks datasets (from refs. ^28,38,39^) at the Hu and Mo *JUNB* promoter (top), 10 kb (middle) or 100 kb (bottom) from transcriptional start site (TSS). Darker links indicate connection to the *JUNB* promoter. *JUNB* gene body is labelled in black. All ATAC peaks scored by the original studies are shown in blue. Stacked bar plots (right) show proportions of enriched motifs (all) and motifs of upstream regulators scored by the CellOracle GRN analysis (presented in **c**). Black, unique motifs present in both Mo and Hu (shared); grey, Mo only unique motifs; light grey, Hu only unique motifs. **b**, Correlation between landscape similarity across species and proportion of motifs within promoters present in both species. **c**, Hu RG *JUNB* indirect (light pink) and direct (magenta) upstream positive regulators predicted by CellOracle analysis. **d**, Hu and Mo *JUNB* promoter accessibility counts per cell type. **e**, Left: Illustration of the *Junb*/*JUNB* promoter and *Irf1*/*IRF1* overexpression constructs. Top right: Representative images of electroporations of either Mo *Irf1* or Hu *IRF1* along with promoter reporter vectors. Bottom right: Histograms showing proportions of TagBFP2^+^ cells also expressing eGFP or Scarlet3. Each dot represents a biological replicate for Ctl (*n* = 4), Mo *Irf1* (*n* = 4), Hu *IRF1* (*n* = 6). Hu *JUNB* promoter Ctl and Hu IRF1 conditions are presented in the Fig. 5 and shown here for clarity. **f**, Left: Experimental design of promoter bashing experiment coupled with *Irf1*/*IRF1* overexpression. Middle: Images representing the electroporations of the mutated JUNB promoter vectors with or without *Irf1*/*IRF1* overexpression vectors. Right: Histograms displaying TagBFP2^+^ cells also expressing Scarlet3. Each dot represents a biological replicate for Ctl (*n* = 4, same data as **e**), Control with mutated pJUNB (*n* = 3), Mo *Irf1* (*n* = 4, same data as in panel **e**), Mo *Irf1* with mutated pJUNB (*n* = 3), Hu *IRF1* (*n* = 6, same data as in panel **e**), Hu *IRF1* with mutated pJUNB (*n* = 3). **g**, Left: Conditions and cell cycle phase prediction of scRNA-seq E16 datasets after *in utero* electroporation of IRF1, EGR1 and IRF1 + EGR1 constructs. Right: Proportions of RG in G1, S and G2/M phase in all conditions. Statistics: *P < 0.05, **P < 0.01, ***P < 0.001 using one-way ANOVA with Tukey correction. Abbreviations: Ctl, control; CP, cortical plate; VZ, ventricular zone; Mo, mouse; Hu, human; d, day.

We next investigated what drives JUNB expression in human but not mouse RG. The *JUNB* promoter is highly conserved between species (92.5%; Methods), but small sequence differences in key regulatory regions could alter transcription factor binding affinities^47^ (Fig. 5a). Consistent with this proposition, transcription factor motif analysis identified species-specific motifs within and near the promoter (up to 10 kb from the transcription start site (TSS)), whereas more distal regions (more than 100 kb from the start site) were more conserved, suggesting a proximal bias in species-specific regulation (Extended Data Fig. 15a).

To test whether these promoter sequence differences are sufficient to explain expression divergence, we co-electroporated E15.5 mouse cortex with two constructs: with the mouse *Junb* promoter driving eGFP expression and the human *JUNB* promoter driving Scarlet3 expression (Fig. 5b). In mouse cells, the human promoter recapitulated the mouse pattern—inactive in RG and active in neurons—indicating that promoter sequence alone does not confer human-specific *JUNB* activation in RG. Consistent with this finding, genome-wide analysis showed no correlation between promoter motif conservation and cross-species gene expression divergence (Extended Data Fig. 15b and Supplementary Table 7).

We therefore examined whether differences in upstream regulators account for human-specific JUNB expression. In silico gene regulatory network (GRN) analysis identified 40 transcription factors, including IRF1, ATF3, STAT3 and EGR1, as potential upstream activators of JUNB in human RG (Fig. 5c, Extended Data Fig. 15c and Supplementary Table 8). Focusing on IRF1, which is exclusively expressed in human RG (Fig. 5c), we confirmed its direct binding to the *JUNB* promoter using CUT&RUN in Org_H_ (Fig. 5d).

IRF1 binding motifs are present and accessible in both mouse and human RG promoters, suggesting that in mouse, *Junb* is primed but inactive, and potentially activatable (Extended Data Fig. 15a,d and Supplementary Table 8). To test this, we co-electroporated the human *JUNB* promoter-Scarlet3 reporter with an IRF1-expressing construct in mouse RG. Although the human *JUNB* promoter was inactive when transfected without IRF1 (Fig. 5b), it became active in the presence of IRF1 (Fig. 5e and Extended Data Fig. 15e). Mutation of IRF1 binding motifs significantly decreased this activation, confirming direct regulatory interaction (Extended Data Fig. 15f). scRNA-seq following IRF1 overexpression showed increased *Junb* expression, demonstrating that IRF1 can activate *Junb* in the mouse RG chromatin context (Fig. 5f). Co-electroporation of EGR1 with IRF1 produced cumulative effects, supporting synergistic regulation (Fig. 5f and Extended Data Fig. 15g). Finally, IRF1 overexpression shifted the mouse RG transcriptional landscape towards that of human RG (Fig. 5g) and produced JUNB-like functional effects: extended neuronal generation and increased G1/S phase RG (Fig. 5h and Extended Data Fig. 15g). Together, these findings demonstrate that human-like developmental programmes can be orchestrated in mouse through experimental manipulation of normally human-restricted upstream regulatory factors (Fig. 5i).

## Discussion

The results presented here provide a comprehensive insight into developmental stage-specific gene expression programmes within the cortical excitatory lineage across species (mouse and human) and context (human in vivo and in vitro). Through systematic analysis of developmental transcriptional landscapes, we reveal that evolutionary conservation varies across cell types: whereas RG and neurons show relatively conserved expression patterns, IPs exhibit clear divergence in gene expression. This finding is consistent with a model of mosaic brain evolution^22^, whereby distinct cell populations evolve at different rates. This may explain why absolute embryonic age is not a principal driver of landscape clustering (that is, why genes with distinct age specificity across cell types are rare). Finally, the high divergence in IPs suggests they may be key drivers of interspecies differences in cortical architecture through species-specific controls of neuronal output.

Our functional analysis of *JUNB* illustrates how temporal regulation of shared genes can orchestrate species-specific features. The ability of JUNB to induce human-like progenitor properties when expressed in mouse tissue suggests the presence of latent transcriptional programmes that can be activated in the right cyto-temporal context. In support of this, the human upstream *JUNB* regulator IRF1, which is absent in mouse, can activate mouse *Junb* promoter when artificially expressed and induce human-like RG properties. Thus, subtle changes in the cyto-temporal expression of conserved genes can produce functionally relevant phenotypic differences. The shift in neuron production timing in JUNB-expressing RG may cumulatively increase the production of late-born neurons, thereby contributing to the evolutionary expansion of superficial cortical layers. Since these layers primarily contain neurons that connect different cortical areas, this seemingly subtle timing change can profoundly impact cognitive capabilities by altering the balance between intracortical—that is, associative—and subcortical communication.

Although we focused on excitatory neurons, similar principles are likely to extend to inhibitory interneurons, whose prolonged development and plasticity make them sensitive to even small interspecies shifts in gene expression timing. This may inform uniquely human features of cortical inhibition and its disorders. The resource at https://www.humous.org can thus complement standard bioinformatic tools (for example, OMIM) by providing cyto-temporal expression profiles that are useful for evaluating variants of uncertain significance.

The comparison between human and Org_H_ data revealed both strengths and limitations of in vitro models. Whereas many developmental programmes were well preserved, we observed differences in cell cycle-related genes in early IPs, suggesting that the high proliferative rate of early IPs in humans is not reproduced in vitro, an aspect that may require environmental signals that are present in vivo, such as factors in cerebrospinal fluid^48^. It will be important to understand the upstream mechanisms controlling these temporal expression differences, including epigenetic regulation, metabolic state and post-transcriptional control^49–51^. Building on the framework established here, such investigations will further inform how genetic programmes, temporal regulation and environmental factors interact to generate species-specific brain features during development and evolution.

## Methods

### Data source and selection

All single-cell transcriptomics data used at https://www.humous.org are from open public datasets from mouse^21,23–25^ and human^26–28^ embryonic cortices, and human-derived cortical organoids^30–33^ (Supplementary Table 1). Gene expression datasets were filtered for cortical excitatory lineage cells across the corticogenesis period (PCW12–PCW24 for human, E12–E17 for mouse, and 1–7 months for Org_H_). We used authors’ original cell annotations for filtering cell types corresponding to RG and IPs, as well as immature and mature neurons, and only selected ages at which RG, IPs and neurons were all detected (all other cells were not included in the analyses).

Whereas comparing cell types (RG, IPs and neurons) across species is relatively straightforward because of typically unambiguous markers, comparing ages of brain development across species is challenging, including because of mosaic evolution, whereby different cell types evolve and mature at different rates^22,52^. Some level of arbitrariness was thus unavoidable, and here, we took the option of aligning cells on the basis of deep-layer to superficial-layer corticogenesis windows and RG:IP:neuron proportions in the original datasets. In humans, the first cortical neurons appear in the preplate around PCW7–PCW8, and the cortical plate emerges around PCW8–PCW10, with the subplate clearly distinguishable by PCW13–PCW15. Neurogenesis of deep-layer neurons goes from PCW10 to PCW15, when the generation of superficial-layer neurons becomes dominant^53,54^, and by PCW18–PCW24, gliogenic progenitors expand^55,56^. In mouse, the preplate forms at E10–E12, and subplate neurons are born around E11. From E12 on, deep-layer neurons are produced, followed by superficial ones from E14 on. By E17, neurogenesis is complete, and progenitors switch to glia production^55,57^. In Org_H_, we focused on stages in vitro during which RG, IPs and neurons were found in substantial numbers in the original datasets, following ref. ^29^.

Use of the terms 'human-specific' and 'mouse-specific' does not imply that the gene or property in question is uniquely restricted to humans or mice across all species. Instead, these terms are used comparatively to indicate that the feature is present in one of the two species examined and absent in the other.

### Data integration and cell-type annotation

We integrated datasets belonging to the same condition to alleviate batch effects. For mouse datasets, we randomly selected 6,000 cells per age group, defined as E12, E13, E14, E15 and E16–17. For human datasets, we randomly selected 6,000 cells per age group, defined as PCW12, PCW14–PCW16, PCW17–PCW18, PCW20–PCW21 and PCW23–PCW24, except for PCW12, for which only 4,867 cells were available. We then used the Seurat v3 integration pipeline^58^ for pairwise dataset integration over the 2,000 most variable features across datasets (VST method). We specified 15 and 20 neighbours for anchoring and regressing out the effect of the total number of genes detected per cell (nFeature_RNA). For organoid datasets, due to partially overlapping group definitions in original data annotations and imbalanced data availability, we aimed at obtaining the same number of cells as for other species (~30,000 cells) and defined flexible cell selection windows per stage in vitro, as follow: ≤2 months: 5,706 cells (named 2 months in landscapes), 2–3 months: 6,294 cells, 3 months: 8,644 cells, 3.5–5 months: 599 cells, and 6–7 months: 8,761 cells. Pairwise integration was referenced to the previously integrated human gene expression matrix (see Extended Data Fig. 1) and Org_H_ integration units were defined as the combination of dataset and protocol to avoid capturing experimental biases in organoid generation. To evaluate batch mixing after integration, we computed the integration Local Inverse Simpson’s Index (iLISI) on the integrated UMAP embeddings, following the approach as described^59^. For each cell, a local neighbourhood of *k* = 30 nearest neighbours was defined in the UMAP space. Within each neighbourhood, we computed the proportion of neighbours belonging to each batch label and calculated the inverse Simpson’s index as iLISI_*i*_ = 1/∑_*b*_ *p*_*i*,*b*_^2^, where *p*_*i*,*b*_ denotes the proportion of batch *b* in the neighbourhood of cell *i*. Higher iLISI values indicate greater batch mixing. This metric was implemented in the Lisi R package (v1.0) for nearest-neighbour calculations.

Each integrated object was used to define differentiation groups consistently across datasets. Integrated gene expression matrices were embedded using two-dimensional UMAP and clustered using unsupervised Seurat clustering. Clusters were then assigned to differentiation groups (RG, IP or neuron) on the basis of the expression of canonical markers (*SOX2*, *EOMES* and *NEUROD6*, respectively), thereby harmonizing cell-type annotations across the original datasets (Extended Data Fig. 1). We then used balanced number of RG, IPs and neurons for each age group, resulting in 2,425 cells per cell type in mouse, 2,450 RG and neurons, and 2,280 IPs in human, and 1,800 per cell type in Org_H_ for ordinal modelling and landscape generation (see Supplementary Table 1).

### Dynamic time-warping alignment of human and mouse developmental stages

To quantitatively align developmental stages between human and mouse, we applied multivariate dynamic time warping on the jointly integrated scRNA-seq data from both species^60^. Alignment was performed in principal component (PC) space using the R package dtw (v1.23) with Euclidean distance and the symmetric2 step pattern, and the full warping path (index pairs and corresponding human and mouse age labels) was extracted. Age-specific PC centroids were computed using a trimmed mean and scaled by the number of contributing cells, with light smoothing applied along each trajectory to reduce local variability. A Sakoe–Chiba constraint was used to limit excessive temporal deviation during alignment. From the full warping path, we constructed a contingency table of visit frequencies for each human–mouse age pair. For each human age, the best-matching mouse age was defined as the most frequently visited along the warping path, and conversely for each mouse age.

To visualize alignment uncertainty, we summarized the distribution of matches using centroid ± interquartile range (IQR) plots in both directions. For each human or mouse age, we calculated the frequency-weighted centroid of matched counterpart ages in rank space and the IQR of ranks encountered along the warping path, reporting the number of distinct contributing stages. Ages with IQR = 0 indicate one-to-one matches, whereas non-zero IQR values reflect overlap across adjacent developmental stages.

### Nearest-neighbour probability analysis

To assess gene expression patterns along the differentiation axis in human, we quantified neighbourhood enrichment using a nearest-neighbour (NN) probability metric in integrated principal component analysis space. Cells were embedded in principal component space derived from the integrated assay, and for each cell its nearest neighbour (1 − NN) was identified using Euclidean distance. For a given marker gene, cells were classified as positive or negative on the basis of normalized RNA expression (>0). For each differentiation states, we calculated the probability that a cell’s nearest neighbour was gene-positive, yielding a stage-wise NN probability profile. Confidence intervals (95%) were estimated assuming a binomial distribution. This analysis was performed independently for each gene and visualized as a continuous trajectory across ordered differentiation states, allowing comparison of neighbourhood-level gene enrichment while accounting for local transcriptional similarity (Extended Data Fig. 6d).

### Ordinal modelling for age and differentiation

To convert qualitative age and differentiation groups into gene expression aware quantitative continua, we used a custom adaptation of support vector machine-based ordinal regression (bmrm R package v4.1; https://github.com/pradosj/bmrm) on integrated data for each condition (joint ordinal model across conditions did not allow prediction of age accurately, indicating that age-encoding programmes are highly species- and context-dependent). For ordinal model training we selected a balanced number of cells, *n* = 50 for each ordinal level combining age and differentiation groups (resulting in 3,750 cells in total for human and mouse, 2,400 for Org_H_; see Supplementary Table 1); the biases due to datasets or protocols were balanced by the integration.

We performed a two-step fitting process to produce a sparse linear model with the same number of features contributing positively and negatively to the linear score. The first step allows ranking of top/bottom features amongst all scaled variable genes (*n* = 2,000), while the second step (reduced model) re-adjusts linear weights after selecting top 50 features (25 for each extreme of the ordinal output). Both fitting steps are done sequentially during the training process, without considering the test data. Twenty cross-validations were implemented for reduced model training, each using different cell subsets. This strategy ensures avoidance of overfitting and is expected to yield an unbiased estimate of performance^61^.

To stabilize and standardize the distribution of ordinal scores, a two-step transformation procedure was implemented. First, a custom function for distribution detection and activation was applied to smooth and rescale (from 0 to 1) the ordinal distributions on the basis of their empirical characteristics towards achieving uniformity and unimodality. Specifically, the unimodality and uniformity of ordinal score vectors’ distribution was assessed using dip (dip.test R function, diptest package v0.77-1) and Kolmogorov Smirnoff testing (ks.test R function, stats R package v4.3.2), respectively. Unimodal and uniform scores were left unchanged. Non-uniform scores followed a smoothing transformation applied to each ordinal target level (age or differentiation groups) and consisting of applying the hyperbolic tangent function (tanh, base R package) over the median of the sub-score with a strength adapted to its standard deviation. Next, we normalized the standardized scores across groups (considering categorical limits) and applied outlier correction (substitution of outlier values by closest within the quantile window of retention: 0.01 and 0.99 probabilities, Winsorize R function, DescTools package v0.99.60). Together, this procedure corrected for differences in cell density for each condition without altering the ordering determined by the machine learning regression model and ensured that positional distributions of cells along the age and differentiation ordinal axes are comparable across datasets with different density characteristics. For a detailed examination of the approach, please refer to custom functions ordi_activate() and ordi_normalize(), both included in the library lib_misc.R in the repository https://github.com/gomez-L/humous and https://doi.org/10.17605/OSF.IO/E8GMA.

To evaluate the agreement between predicted and actual ages across cell types and conditions, we calculated non-parametric rank correlations. Spearman’s rank correlation coefficient (*ρ*) and Kendall’s tau (*τ*) were computed for each experimental group using the cor.test() function in R. Spearman’s *ρ* quantifies the strength and direction of a monotonic relationship between predicted and true ages, while Kendall’s *τ* assesses the ordinal association and provides a more conservative measure in the presence of tied ranks. Both correlations were calculated with the asymptotic approximation for *P* values (exact = FALSE) to account for tied observations. For each correlation, we report the coefficient as a measure of effect size. The associated two-sided *P* values were lower than 0.0001 in all conditions and cell types.

### Gene expression landscapes

#### Data filtering

Before landscape generation, we excluded genes expressed in less than 1% of the cells across datasets within a condition. As a result, we selected for calculating gene landscapes *n* = 12,757, *n* = 12,714, and *n* = 13,564 genes for human, mouse and Org_H_ datasets, respectively. Expression for all these genes was normalized and scaled regressing out the total number of genes detected per cell (nFeature_RNA).

#### Landscape generation

For each condition independently, we used scaled scRNA-seq data and applied the following procedure: we first constructed a *k* nearest neighbours array (*k* = 100) using the cells’ age and differentiation ordinal scores as neighbourhood coordinates. Next, for each gene, we averaged its expression across blocks of 100 neighbour cells to construct a smoothed expression array—a gene landscape—of dimension 250 × 250 (averaged expression of cell groups; that is, pixels), as we have done before^18,21,62,63^. To test whether using lower or larger neighbour cells for averaging would impact landscape patterns, we generated all human landscapes for patterned genes by averaging 50 or 200 neighbour cells and compared those to the 100 neighbours’ original landscapes. The gene expression pixel to pixel mean correlations was highly correlated between the three conditions (50 versus 100 neighbours mean correlation = 0.80, 200 versus 100 neighbours mean correlation = 0.86). Each gene landscape was stored for further analysis as an array and as a png image (using a custom R function that combines the v3.5.2 and ggplot2 v3.5.1 R libraries, png dimensions were set at 244 × 244 pixels for optimal functioning of computer vision modelling, see below).

### Definition of gene expression landscape profiles based on entropy and standard deviation

Landscape profiles were defined through deterministic thresholding based on the joint distribution of entropy and standard deviation values. A global entropy measure made over each entire landscape image (not on local spatial variations) was used. Specifically, the Cauchy–Schwarz entropy was calculated with the R package entropy (v1.3.0), on 0–1 ranged and scaled landscape image arrays (greyscale and made of non-negative numbers) for assessing the pixel intensity distribution complexity (overall texture). We used the Cauchy–Schwarz entropy, which integrates all pixel values into a single distribution and quantifies the overall complexity of the entire landscape, as a metric for identifying noise structures in the landscapes. The standard deviation (s.d.) was calculated using the R function sd on the gene landscape vectorized array (as it is a measure of distribution), while the entropy (*E*) was calculated on the image matrix (to measure the level of randomness in pixel patterning). Landscape expression profiles were defined as follows: *E* < 20 and s.d. < 0.1, ‘low’; 20 < *E* < 25 and 0.1 < s.d. < 0.15, ‘non-patterned’; s.d. > 0.15, ‘patterned’; and E > 25 and 0.15 < s.d. < 0.35,“‘ubiquitous’. Genes with low expression profiles were expressed by very few cells sparsely located on the age–differentiation axes, genes with non-patterned profiles by many cells distributed in a random manner on age–differentiation axes, genes with ubiquitous profiles by many cells homogeneously located on age–differentiation axes, while genes with patterned profiles were expressed by few to numerous cells but with a clear pattern on age–differentiation axes (see Extended Data Fig. 7).

### Landscape computer vision feature extraction

For extracting spatially relevant features of gene landscapes, we encoded them using a computer vision model, VGG16, pre-trained on the ImageNet dataset, accessed using the keras API through an R miniconda with Python (v3.7). Specifically, we cut the sequential model layers to retrieve the output from the fifth max pooling layer, providing 25,088 features per landscape. Gene landscape 244 × 244 png files were pre-processed for VGG16 prediction to be converted to arrays of shape [1,7,7,512]. Next, we launched the model prediction on each image and flattened the output to obtain a feature vector (1 × 25,088), all finally bound in a data matrix to be used for clustering. The features obtained here are non-linear combinations that represent patterns, such as edges, textures, and spatial organization (similar to how visual information is hierarchically processed—for example, in refs. ^64,65^), which capture higher-order, biologically meaningful structures in the gene expression landscapes.

### Robustness of gene expression landscapes

To ensure reproducibility of the gene expression landscapes, multiple permutations of the cell subsampling were performed on the curated datasets using the same pipeline as described above. In total, five subsampling permutations were performed in human and Org_H_, and four in mouse. Cells were then further balanced for the training of the ordinal regression models and landscapes were generated on the basis of the different combination of subsampled cells. The generated landscapes were then correlated per gene to the original landscapes to assess reproducibility (Extended Data Fig. 5).

### Gene landscape clustering based on computer vision features

Gene landscapes of the three conditions were compiled into a Seurat object using Seurat R package (v3), with the assay containing their 25,088 VGG16-extracted features, and the metadata all information about each landscape, including their entropy, s.d., and assigned profile. Iterative clustering was next used to cluster the landscapes. For this, we normalized the features and defined the 2,000 most variable ones using the FindVariableFeatures function. Data were scaled and the principal components calculated using these variable features. Clustering iterations were performed using 1:15 principal component. The first clustering iteration (resolution = 0.2) enabled us to identify landscapes with low, non-patterned, expression profiles, validating our classification based on entropy and s.d. values. After excluding these landscapes, the variable features were redefined, as well as the principal components, neighbours and clusters (resolution = 0.5). This second clustering iteration allowed labeling of two more clusters with non-patterned expression profiles. Following the same strategy, we excluded these landscapes and made a third clustering iteration (resolution = 1.3) using only the patterned landscapes (defined on the basis of entropy and standard deviation, see above), which resulted in 22 clusters of gene expression landscapes.

#### Annotation of expression landscapes

Clusters of gene expression landscapes were manually annotated on the basis of their age (early, late or non-specific) and cell type (RG, RG-IP, RG-N, IP, IP-N, N or non-specific) specificities, by averaging 20 random landscapes per cluster using a standard base R mean of the array of landscapes.

### Pseudotime trajectories of gene expression landscapes

To build each trajectory, the numerical representation of patterned landscapes in the UMAP space were considered and used as input for R package Monocle3 workflow (v1.4.20). The previously described Seurat object was converted into a cell_data_set object with the help of SeuratWrappers (v0.3.5) package and processed with the classic Monocle3 Trajectories workflow (https://cole-trapnell-lab.github.io/monocle3/docs/trajectories/)^66^. The cluster_cells function was used with the *k* = 10 and reduction_method = “UMAP” parameters, the learn_graph function with use_partition=F and minimal_branch_len=3 parameters, and the order_cells function with default parameters. The Choose_graph_segment function was used to connect starting and ending nodes, respectively corresponding to the less- and most-advanced branching point of the pseudo-differentiation tree. Only the main trajectory per condition is shown.

### Landscape correlations

Aiming at obtaining a comparable numerical metric of landscape similarity, we calculated correlations between each gene landscape and all others across intra- and interspecies (pairwise and non-pairwise correlations). We defined a custom R function taking the 0–1 normalized vectorial representation of two landscapes and thresholding it at 0.3-pixel intensity value for diminishing the impact of noise on correlation estimates. As correlation is impacted by sample size, a robust implementation was set in which, for each pair of landscapes to compare, we bootstrapped the calculation 10 times for a fixed set of 2,000 pixels (sampling with replacement) equally placed on the two landscapes. Finally, we calculated the median across the ten bootstrapped correlations. Gene landscapes with a bootstrapped correlation coefficient higher than 0.5 across conditions were tagged as highly correlated, while landscapes with correlation lower than −0.5 were tagged as anti-correlated.

### Gene lists

Disease-associated genes are from the OMIM (Online Mendelian Inheritance in Man) database^67^ and reflect proximity to genome-wide association study hits. To look at genes with specific functions, we used published databases of mammalian metabolic enzymes^68^ and mitochondrial proteome^69^ (MitoCarta3.0), SingleCellSignalR (v2) for ligands–receptors^70^, and transcription factors^71^.

Genes with given ontologies were analysed using ontologyIndex R package (v2.7) from the ontologyX suite^72^.

### Humous.org website

An interactive web platform (https://www.humous.org) was made available to serve both as a user-friendly landscape database (‘Browser’ and ‘Gene sets’), and to incorporate additional functionality to guide discovery for further research (‘Correlations’ and ‘Draw your landscape’). A summarized version of computational methods and data sources is also included (Methods). The platform combines a JavaScript front-end and an R back-end, containerized in a docker network and hosted at University of Geneva servers. The browser page allows searching for a gene (or series of genes) of interest and to retrieve the respective landscapes across species. A tabular display enables a friendly layout for landscape comparison. The gene sets page behaves similarly but encodes a priori defined groups of genes on the basis of function or specificity. The correlations page enables users to recursively find genes that are similar or dissimilar within and across species, therefore guiding the discovery of candidate genes for further research (see landscape correlations methods section). The draw your landscape page encodes a landscape correlation-based engine for retrieving the top best landscape matches on the basis of a user input drawing.

### Gene ontology analyses

Gene ontology over-representation analysis was performed for biological processes using the enrichGO function from the clusterProfiler package (v3.2.1)^73^, with the following parameters: pAdjustMethod = “BH”, pvalueCutoff = 0.05, qvalueCutoff = 0.05, minGSSize = 10, maxGSSize = 500. While top ontologies are shown in figures for readability, significant ontologies are in Supplementary Tables 3 and 4.

### Plasmids

The pCAG-GFP-ITR vector was a generous gift from C. Mayer, which was modified to either include H2B sequence upstream to GFP (pCAG-H2B-GFP-ITR) or TurboRFP-NLS instead of the GFP (pCAG-TurboRFP-NLS-ITR). Addgene #29687 pCS2-Flag-JUNB was used for JUNB overexpression experiments. Addgene #175573 pX458-Ef1a-dCas9-KRAB-MECP2-H2B-GFP (CRISPRi) was modified by replacing the Ef1a promoter with CAG using NEB HiFi DNA assembly reaction (NEB: E2621S). pCAG-CRISPRi plasmid generated in this study was then digested with BbsI-HF (NEB: R3539S) and using NEB HiFi DNA assembly, gRNA1: GAGGCCAGCCTCGGAGCCAGC and gRNA2: GCCAGCTCCCTGCTGGCTCCG was cloned into the backbone. pCAG-CRISPRi without a gRNA was used as a control. Addgene #153520 tFucci(SA)5 was used for cell cycle phase analysis experiments. pCAG-IRF1 (human), pCAG-Irf1 (mouse) and pCAG-TagBFP2-NLS were cloned by ordering IDT gBlocks of ensembl human *IRF1* (IRF1-201), mouse *Irf1* (Irf1-201) coding sequences, and TagBFP2 (ref. ^74^) fused with NLS domain^75^. pCAG-Cre (Addgene #13775) was digested with EcoRI and NotI and replaced with either of the *Irf1*/*IRF1*/TagBFP-NLS cDNA gBlocks using NEB HiFi DNA assembly reaction mix. Mouse *Junb* and human *JUNB* promoters were first visualized on the rVista2 browser for conservation and 1 kb sequences upstream to the TSSs were chosen^76^. These sequences were cloned into AiP981-pAAV-mscRE4-minBGpromoter-eGFP-WPRE-hGHpA (Addgene #163484) by first digesting the backbone with MluI and EcoRI to remove the promoter and eGFP fragments. The backbone was then combined with ordered gBlocks of the human or mouse promoter DNA sequence 1 kb upstream from the TSS in the mm10 or hg38 genomes and either eGFP^77^ or mScarlet3 (ref. ^78^) fused with PEST domain^79^. NEB HiFi DNA assembly reaction mix was used to generate pJunb-eGFP-PEST or pJUNB-Scarlet3-PEST plasmids. Mutagenesis of the pJUNB-Scarlet3-PEST was performed by digesting the backbone with KpnI and EcoRI. Two closely located IRF1 DNA-binding motifs^80^ (GAAA) were replaced with ATGC and the fragment was ordered as an IDT gBlock to clone into the digested backbone to generate pJUNB-Irf1mut-Scarlet3-PEST. EGR1 overexpression was performed using TFORF3412 vector (Addgene #144888). CAG-mGreenLantern (mGL)^63^ vector was used as a reporter for IRF1 overexpression experiments.

### iPSC lines and maintenance

Induced pluripotent stem cells (iPSCs) (HPS00076:409B2 and GM05399) were cultured on Matrigel (Corning) coated plates (Thermo Fisher) in mTeSR1 basic medium supplemented with 1× mTeSR1 supplement (STEMCELL Technologies) at 37 °C, 5% CO_2_, and ambient oxygen level. Passaging was done by Gentle Cell Dissociation Reagent (STEMCELL Technologies) treatment.

iPSCs (PC1505, female apparently healthy line bought from Phenocell, now part of Axol Bioscience) were cultured on Matrigel (354277, Corning) coated plates (TPP) in complete mTeSR Plus medium (STEMCELL Technologies) at 37 °C, 5% CO_2_, and ambient oxygen level. Passaging was done using Accutase (STEMCELL Technologies).

iPSCs were authenticated by the original vendors using karyotyping analysis. iPSCs were periodically checked for Mycoplasma contamination and were negative.

### Generation, culture and electroporation of cerebral organoids

#### Organoids used for TBR2 and Ki-67 staining and electroporation

Cerebral organoids were generated from iPSCs at ~80% confluency according to previously published protocols^81,82^. Cells were dissociated with Accutase and resuspended in low bFGF medium with 50 μM ROCK inhibitor. Live cells (2,000 per well) were seeded into low-attachment 96-well plates, forming embryoid bodies after 24 h. Embryoid bodies were cultured for 5–6 days and subsequently transferred to neural induction medium and cultured for 4–5 days. Neuroepithelial tissues formed, showing radially organized pseudo-stratified epithelium. On day 11, aggregates were embedded in Matrigel and cultured further. By day 18, organoids were placed on an orbital shaker at 57 rpm, with medium changes every 3–4 days, supporting cerebral tissue development. For electroporation of cerebral organoid ventricles, organoids with well-formed ventricles were selected at day 43 as previously published^83,84^. Prior to electroporation, plasmid DNA was prepared at a concentration of 1–2 μg μl^−1^ in sterile nuclease-free water, with 0.05% Fast Green added to visualize injection. Microcapillaries were pulled from borosilicate glass using a Narishige PC-10 micropipette puller with one-step pulling at a heater level of 62 °C and 4 weight blocks. The capillaries were loaded with the plasmid solution via capillary action. Each organoid was transferred individually into an electroporation dish containing pre-warmed (23 °C) Opti-MEM. Using a stereomicroscope within a horizontal flow cabinet, microcapillaries were used to inject 5 μl of plasmid solution into the ventricles of each organoid, targeting specific lobes of interest. After injection, electroporation was performed using a BTX Harvard apparatus at 80 V with 5 pulses of 1 ms each and 1-s intervals. Following electroporation, the organoids were transferred to fresh medium and allowed to recover before further processing 7 days after electroporation. Cerebral organoids contain multiple ventricular structures. All morphological and cellular quantifications were therefore performed at the ventricle level. To assess reproducibility across organoids, ventricle-level measurements were additionally summarized per organoid and used for quality control and data visualization (Extended Data Fig. 12).

#### Organoids used for CUT&Tag and CUT&RUN analyses

Cerebral organoids were generated from iPSCs PC1505 line at ~80% confluency according to STEMdiff Cerebral organoid kit from STEMCELL Technologies (a standardized implementation of unguided cerebral organoid differentiation). Cells were dissociated with Accutase and resuspended in Organoid formation medium with 50 μM ROCK inhibitor. Live cells (5,000 per well) were seeded into low-attachment 96-well plates, forming embryoid bodies after 24 h. Embryoid bodies were cultured for 5 days and subsequently transferred to Neural Induction Medium and cultured for 2 days. Neuroepithelial tissues formed, showing radially organized pseudo-stratified epithelium. On day 7, aggregates were embedded in Matrigel in Expansion Medium and cultured further. By day 10, organoids were placed in Maturation Medium, with medium changes every 3–4 days, supporting cerebral tissue development.

### Human tissue collection and processing

Human fetal brain tissue was collected following voluntary pregnancy terminations performed at CHR Hospital–Site Laveu (Liège, Belgium). All procedures were conducted in accordance with the Declaration of Helsinki, Good Clinical Practice and General Data Protection Regulations, with written informed consent obtained from all mother donors. Ethical approval was secured under protocol number 2021/216 by the University Hospital-Faculty Ethics Committee of Liège. Gestational age (PCW10) was determined by ultrasound and verified through fetal tibial length measurement and case was examined with standard feto-pathological procedures^85^ and did not display clinical or neuropathological evidence of brain malformation. Following surgery, forebrain tissue sample was retrieved immediately and transferred to the laboratory within 1 h in oxygenated artificial cerebrospinal fluid (aCSF; 85 mM NaCl, 2.5 mM KCl, 7 mM MgCl_2_, 0.5 mM CaCl_2_, 1 mM NaH_2_PO_4_, 26 mM NaHCO_3_, 10 mM D-glucose, 50 mM sucrose). The sample was embedded as whole in OCT compound (Sakura), rapidly frozen in liquid nitrogen, and stored at −80 °C until further analysis.

### Mouse strains

CD1 pregnant female mice from Charles River Laboratory were used. The experimental procedures described here were conducted in accordance with the Swiss laws and previously approved by the Geneva Cantonal Veterinary Authority. All mice were housed in the institutional animal facility under standard 12 h:12 h light:dark cycles with food and water ad libitum.

### In utero electroporation of mouse embryos

In utero electroporations were performed as previously described^86^. Timed pregnant CD1 mice were anaesthetized with isoflurane (5% induction, 2.5% during the surgery) and treated with the analgesic Temgesic (Reckitt Benckiser). Embryos were injected in the lateral ventricle with ~1 μl of DNA plasmid solution diluted in endotoxin-free TE buffer and 0.002% Fast Green FCF (Sigma). Embryos were then electroporated by holding each head between circular tweezer-electrodes (5 mm diameter, Sonidel) across the uterine wall, while 5 electric pulses (50 V, 50 ms at 1 Hz) were delivered with a square-wave electroporator (Nepa Gene, Sonidel).

Embryos within the same litter were randomly allocated to receive either control or experimental plasmids. Sex was not used as a covariate for allocation, as tissue was collected at developmental stages prior to sexual maturation.

### BrdU and EdU injection

On average, 350 μl of 10 mg ml^−1^ BrdU (B23151; Thermo Fisher Scientific) or 400 μl of 7.5 mg ml^−1^ EdU (A10044; Thermo Fisher Scientific) resuspended in 0.9% NaCl was intraperitoneally injected into pregnant females corresponding to 200 mg kg^−1^ (10 μl per g body weight).

### Tissue processing and immunohistochemistry

#### Organoid and human tissue

For immunohistochemistry, organoid or human tissue 20-µm-thick sections were prepared on slides, which were thawed and rehydrated by rinsing in phosphate-buffered saline (PBS) for 5 min. The sections were blocked and permeabilized using a solution of 0.25% Triton X-100, 150 mM glycine, and 4% normal goat serum (NGS) in PBS for 1 h at room temperature in a humidified chamber. For immunohistochemistry against TBR2, heat-induced epitope retrieval (HIER) was carried out in 0.01 M sodium citrate buffer (pH 6.0) containing 10% glycerol using a microwave method (500 W for 3 min followed by 200 V for 12 min). Slides were allowed to cool for 20 min at room temperature, rinsed in PBS, and then processed further. The following primary antibodies were diluted in blocking solution (4% NGS, 0.1% Triton X-100 in PBS) and applied to the slides overnight at 4 °C: monoclonal mouse anti-SOX2 (1:500; AMAb91307; lot MAB-04006; cloneID CL4716; Atlas), sheep anti-TBR2 (1:150; AF6166; lot CDIP0319091; R&D Systems), chicken anti-GFP (1:1000; GFP-1020; lot GFP917979; Aves Labs), monoclonal rabbit anti-NEUROD2 (1:300; ab109406; lot GR3249611-4; cloneID EPR5135; Abcam), rabbit anti-JUNB (1:100; 10486-1-AP; lot 00039797, Proteintech). After washing 3 times in PBS with 0.1% Tween for 5 min each, corresponding secondary antibodies from ChromoTek (alpaca anti-mouse IgG1 nanobody: A488: sms1AF488-1, AB_2827578; A568: sms1AF568-1, AB_2827578; A647: smsG1CL647-1, AB_2941312) and Thermo Fisher Scientific (goat anti-rabbit IgG (H + L): A546: A-11010, AB_2534077; A647: A-21244, AB_2535812; goat anti-chicken IgY (H + L) A488: A32931TR, AB_2866499) were diluted at 1:500 and applied for 1–2 h at room temperature in the dark. A final wash in PBS without Tween was performed, and the slides were mounted for imaging with AquaPoly Mount.

#### Mice

Pregnant female mice were euthanized by cervical dislocation, and the embryos were removed from the abdominal cavity and placed in 1× HBSS for dissection. The embryos were first decapitated and transferred to a second petri dish in fresh 1× HBSS before the brain was dissected out. The electroporated cortices were screened for GFP or TagBFP2-NLS (depending on the experiment), and brains were placed in freshly prepared 4% paraformaldehyde (PFA) overnight at 4 °C. P1 pups were injected with Esconarkon and perfused with 1× PBS followed by 4% PFA. Brains were then dissected out from the head and kept overnight in 4% PFA at 4 °C.

The next day, the samples were washed with 1× PBS and 20% sucrose in PBS was added to cryoprotect the samples. The samples were then embedded in OCT and frozen on dry ice. Finally, the tissue was cut in 20-μm coronal sections using Leica Cryostat 3050S and mounted on Superfrost plus adhesion slides, which were kept at −20 °C. Control and JUNB electroporated samples were always mounted on the same slides and immunostained together. The slides were then washed with 1× PBS 3 times and placed in blocking buffer containing 3% BSA, 0.05% Triton in 1× PBS for 1 h. The following primary antibodies were added to the slides in fresh blocking buffer and incubated overnight at room temperature: rabbit anti-SOX2 (1:200; ab97959; lot 1033979-3; Abcam), mouse anti-EOMES (1:100; WD1928; lot 3009178; Thermo Fisher Scientific), rabbit anti-Ki-67 (1:100; AB15580; lot GR250214; Abcam), rat anti-EOMES (1:200; Dan11mag; lot 4348755; Invitrogen), rabbit anti-NEUROD2 (1:400; ab104430; lot GR118831-4; Abcam), chicken anti-GFP (1:1000; ab13970; lot 1018752-11; Abcam), rat anti-BrdU (1:500; ab6326; lot GR3365969; cloneID BU1/75 (ICR1); Abcam), rat anti-RFP (1:500; 5F8; lot 90228002AB-06; cloneID 5F8; Proteintech), rabbit anti-JUNB (1:50; 10486-1-AP; lot 00039797, Proteintech), monoclonal mouse anti-PAX6 (1:200, 13B10-1A10; lot ZK401725; ThermoFisher Scientific). After 3 washings with 1× PBS, the corresponding secondary antibodies were added at room temperature for 2 h: goat anti-rabbit A555 (A-11008), goat anti-chicken A488 (A-11039), goat anti-rat A555 (A-21434), goat anti-rat 647 (A-21247) or goat anti-mouse IgG1 647 (A-21240). All secondary antibodies were from ThermoFisher Scientific and diluted at 1:500 in 1× PBS. DAPI was used as a counterstain at the end of the immunostaining protocol and slides were mounted with coverslips using Fluoromount-G mounting medium (00-4958-02; ThermoFisher Scientific).

EdU was revealed using a custom protocol as previously described^63^ at the end of the immunostaining protocol. Control versus Junb: BrdU was revealed using a previously described protocol^87^ in which slides were incubated with primary antibodies, including anti-BrdU, overnight at room temperature in 1× Exonuclease buffer containing 0.1 U μl^−1^ of Exonuclease III (EN0191; ThermoFisher Scientific). Control versus IRF1: BrdU was revealed by incubating with 2 N HCL for 30 min at 38 °C and then neutralizing with Sodium Borate/Borax for 10 min as previously described^63^. Secondary antibodies were added as described above.

### Image acquisition and analyses

#### Organoids and human tissue

Images were acquired using a MICA Leica microscope with either a 20× air objective, a 43× oil immersion objective or a 63× water immersion objective, depending on the experimental requirements. The microscope settings were adjusted accordingly to optimize resolution and image quality for each magnification. For quantification of GFP^+^ CRISPRi cells in electroporated ventricles, all GFP^+^SOX2^+^, GFP^+^NEUROD2^+^ and GFP^+^TBR2^+^ cells were counted, and double positive cells were normalized to the total number of electroporated GFP^+^ cells.

#### Mice

Control, IRF1, and JUNB samples were imaged at the confocal microscope Zeiss LSM800/880 and Nikon Ti2 Eclipse depending on the assay (controls were always imaged with corresponding experimental conditions on the same microscope). For embryonic stages, three sections per embryo were imaged and analysis was focused only on EdU^+^GFP^+^ cells with SOX2 and/or EOMES positive signal after image acquisition. SOX2^+^EOMES^−^ cells in the VZ were considered as RG, EOMES^+^ cells in the VZ/SVZ were considered as IP, and SOX2^−^ cells in the VZ/SVZ and EOMES^+^ cells in the CP were considered as neurons. At P1, 3–4 sections per pup were imaged and analysed and values were averaged per animal: NEUROD2^+^BrdU^+^GFP^+^ neurons in the cortical plate were quantified and compared to all NEUROD2^+^GFP^+^ cells. Due to sparsity of the FUCCI vector electroporation, only one large region with at least 5 *z*-stacks were imaged per biological replicate. All FUCCI positive cells in the VZ/SVZ were counted per image. h2-3^+^AzaleaB5^−^ cells were considered as S/G2/M, h2-3^−^AzaleaB5^+^ as G1-only and h2-3^+^AzaleaB5^+^ as G1/S transitioning cells. Promoter assays: due to the sparseness of the electroporation, only one large electroporated region of each condition was analysed. Sections were imaged under 2 days after immunostaining to avoid loss of unstained BFP^+^ signal. Scarlet3 was stained with anti-RFP antibody and eGFP was stained with anti-GFP antibody before analysing.

In all conditions, cells were hand counted using a Fiji Cell Counter^88^ in a blind manner using Fiji ImageJ (v1.54f), and proportional values were compiled in Microsoft Excel (v16.107) and Graphpad Prism (v10.0.1).

### Statistics

All statistical analyses were performed using either Graphpad Prism 10 or Rstudio. No statistical analysis was performed to pre-determine sample size.

#### Organoid cell-type proportions and VZ thickness

All quantifications were performed at the ventricle level. To evaluate consistency across biological replicates, ventricle-level data were also grouped by organoid and summarized as per organoid averages for visualization and quality control using two-tailed *t*-test. Statistical analyses based on ventricle-level measurements were performed using two-way ANOVA after passing Shapiro–Wilk normality test to compare control and *JUNB* knockdown electroporated cells.

#### Embryonic cell-type proportions

Unpaired two-tailed *t*-test was used to compare the RG:IP-N ratio between control and JUNB electroporated cells, after passing Shapiro–Wilk normality test.

#### Birthdating

Unpaired two-tailed *t*-test was performed to compare the proportions of BrdU positive control and JUNB or IRF1 electroporated neurons in the CP, after passing Shapiro–Wilk normality test.

#### Clone size

*χ*^2^ test was performed to compare control and JUNB clone size distributions.

#### Cell cycle phases

Non-parametric Mann–Whitney test was performed per cell cycle phase measurement—that is, G1, G1/S and S/G2/M.

#### Promoter assays

Unpaired two-tailed *t*-test or one-way ANOVA with Tukey correction (see Fig. 5 legend) was performed on the proportions of cells with positive reporter signal between conditions, after passing Shapiro–Wilk normality test.

### CUT&RUN and CUT&Tag tissue processing and analysis

#### Organoids

Experimental groups were established by selecting healthy, morphologically similar organoids from the same culture batch. Two PC1505-derived cerebral organoids at DIV40 were dissociated using the Miltenyi Neural dissociation kit by heating the samples in enzyme mix 1 for 15 min and enzyme mix 2 for 10 min. After passing through a 70-μm filter, cells were incubated in 0.1% PFA for 10 min on ice then quenched with 0.5 M glycine if processing for CUT&RUN or proceeded as unfixed cells if processing for CUT&Tag. CUT&RUN (86652; Cell Signaling Technologies) and CUT&Tag (77552; Cell Signaling Technologies) assay kits were used with a few modifications to obtain purified DNA for library preparation as outlined by the manufacturer’s guidelines. Antibodies for CUT&RUN: Rabbit anti-H3K4me3 (1:50; 9751; lot 16; CST), Rabbit anti-IRF1 (1:50; 11335-1-AP; lot 00173582; Proteintech), Normal Rabbit IgG (1:50; 2729; lot 11; CST). Antibodies for CUT&Tag: Rabbit anti-JUNB (1:50; 10486-1-AP; lot 00039797; Proteintech), Normal Rabbit IgG (1:50; 2729; lot 11; CST). A secondary antibody, Goat anti-Rabbit IgG (1:100; 35401; CST) was incubated for 30 min after the primary antibody in both CUT&RUN and CUT&Tag assays. CUT&RUN libraries were generated using Illumina TruSeq ChIP library preparation kit. CUT&Tag libraries were amplified with 14 cycles as described^89^.

#### Mouse

E15 electroporated cortices with either mGL (5 cortices) or mGL+hJUNB (4 cortices) were collected 24 h later, electroporation regions dissected and dissociated into single-cell suspensions as described previously with the E16 collected scRNA-seq datasets. Fluorescence-activated cell sorting (FACS) was performed using BD FACSMelody and BD FACSChorus software (v6.1) and 36,000 cells were loaded for each antibody for the CUT&Tag protocol as detailed above. Antibodies used were: Rabbit anti-H3K27ac (1:50; ab177178; lot GR3203987-19, Abcam), Goat anti-Rabbit IgG (1:100; 35401; CST). Library amplification was performed as described above.

Sequencing was performed using the Illumina NovaSeq 6000 platform using paired-end 50 base pair reads (PE-50).

### CUT&RUN and CUT&Tag analysis

#### CUT&RUN

Fastq files were processed as outlined by 4DN CUT&RUN processing pipeline^90^. In brief, Fastq files were trimmed using trimmomatic^91^ (v0.39) (ILLUMINACLIP:2:30:10; LEADING:3; TRAILING:3; SLIDINGWINDOW:4:15; MINLEN:25). Trimmed reads were then aligned with human (GRCh38-noalt) and yeast (R64-1-1) spike-in reference genomes using bowtie2 (v2.5.4)^92^ (v2.5.4) using the –dovetail option. Sambamba^93^ (v1.0.1) was used to generate indices, SAM to BAM conversion and sorting.

#### CUT&Tag

Fastq files were aligned with either human (GRCh38-noalt), mouse (mm10) or *Escherichia coli* genome (str. 536) using bowtie2 (v2.5.4)^92^. Spike-in depth was quantified to normalize in later steps. Duplicates were removed using Picard^94^ (v3.4.0). Reads mapped to ENCODE blacklist regions were excluded using bedtools^95^ (v2.31.1). Samtools^96^ (v1.22.1) was used to generate indices, SAM to BAM conversion and sorting.

Once the CUT&RUN and CUT&Tag bam files were pre-processed, Deeptools^97^ (v3.5.6) bamCoverage function was used to generate spike-in normalized bigwig files for visualization. SEACR^98^ (v1.3) was used to call peaks with a *P *value threshold of 0.01. The.bed peaks were then uploaded to GREAT^99^ (v4.0.4) to generate peak to gene associations. IGV genome browser^100^ (v2.18.2) was used to visualize bigwig and generate coverage plots.

### Collection and snRNA-seq and scRNA-seq of electroporated cells

#### E16.5 dataset

Twenty-four hours following electroporation, a pregnant female mouse was anaesthetized and euthanized. Embryos from the pregnant mouse were collected. Embryos were first separated on the basis of electroporated plasmids, decapitated and the cortex was dissected out in 1× HBSS.

JUNB: The GFP^+^ cortices were separated, and the electroporated region was dissected and frozen straight away in 2 ml Eppendorf tubes. Four cortices were collected per condition. On the day of the single-nucleus RNA sequencing (snRNA-seq), samples were first gently thawed on ice and resuspended in 100 ul of lysis buffer composed of: 10 mM Tris-HCl (pH 7.4), 10 mM NaCl, 3 mM MgCl_2_, 0.01% NP-40, 1% BSA and 1 mM DTT. After 5 min of lysis on ice, 1 ml HBSS was added. The samples were then centrifuged at 500*g* for 5 min. After removing supernatant, the pellet was resuspended in 1 ml washing buffer composed of: 10 mM Tris-HCl (pH 7.4), 10 mM NaCl, 3 mM MgCl_2_, 1% BSA, 1 mM DTT and 0.5 U μl^−1^ RNAase inhibitor. Then, using the P1000 pipette, the samples were pipetted up and down ten times. The samples were then centrifuged again for 5 min at 500*g*. Supernatant was removed, 1 ml of additional washing buffer was added, and the pellet was resuspended. At this point, no clumps were seen, and the samples were passed through a 30-μm filter before FANS. Hoechst was used as a positive nuclei marker and 25,000 events representing Hoechst^+^GFP^+^ nuclei were sorted directly into empty 1.5 ml Eppendorf tubes using Beckman Coulter MoFlo Astrios with SUMMIT (v6.3.1). Exactly 42.8 μl of the sorted nuclei were loaded on the first step of the 10X Genomics Chromium Single Cell v3.1 reagent kit on the Chromium controller.

IRF1/EGR1: The TagBFP2–NLS^+^ cortices were separated, and the electroporated region was dissected and processed for cell dissociation using the Miltenyi Neural dissociation kit as described previously^63^. Single-cell suspensions were then passed through a 70-μm filter before FACS. Only 20,000 BFP^+^ cells were sorted into separate tubes for either Control, IRF1, EGR1, or IRF1 + EGR1 using BD FACSMelody and BD FACSChorus software (v6.1). The FAC-sorted cells were centrifuged for 5 min at 300*g* and all but 42.8 μl was removed from the tube before resuspending once more and loading on the Chip G for 10X Genomics Chromium Single Cell v3.1 reagent kit on the 10x Chromium X controller.

The rest of the protocol was followed according to the manufacturer’s guidelines. The cDNA libraries were quality controlled using Agilent 2100 Bioanalyzer and TapeStation. Sequencing was performed using the NovaSeq 5000 platform with a 28/90 read configuration.

#### P0 dataset

Four days after electroporations and after birth of pups, four littermates per condition were euthanized by decapitation and the cortex was dissected out of the head in cold 1× HBSS in less than 5 min. The electroporated region was microdissected in a fresh petri dish containing 1× HBSS and transferred into an eppendorf tube. Dissociations were performed using the Neural Dissociation kit (130-092-628; Miltenyi) by treating the microdissected tissue with Enzyme mix 1 for 7 min and then Enzyme mix 2 for 3 min. Cells were washed in sorting buffer (1× PBS, 1% BSA and 0.5 U μl^−1^ SUPERase-In RNase inhibitor (AM2694; ThermoFisher Scientific)) twice at 4 °C with 300*g*. Then, 16,000 control and 50,000 JUNB electroporated RFP^+^ cells were sorted into separate tubes containing sorting buffer using BD FACSMelody and BD FACSChorus software (v6.1). Sorted cell suspension was centrifuged again for 5 min at 4 °C with 300*g* and all but around 43.2 μl was removed. The cell suspension was resuspended gently and processed with 10X Genomics Chromium Single Cell 3′ v3.1 reagent kit on the Chromium X controller. cDNA was split to generate a gene expression and a lineage barcode library as previously described^101^. The quality control of the cDNA and libraries was performed using Agilent’s 2100 Bioanalyzer. Sequencing was performed using the NovaSeq 5000 platform with a 28/90 read configuration, aiming for a depth of 20,000 reads per cell for gene expression library and 2,000 reads per cell for barcode library.

### snRNA-seq and scRNA-seq data analyses

#### Data preprocessing and quality controls

Fastq files were mapped on the standard 10X mouse reference genome refdata-gex-mm10-2020-A using cellranger^102^ (v7.2.0) for the E16 collected snRNA-seq dataset. For the E16 collected scRNA-seq, fastq files were mapped with the mouse reference genome, including human *EGR1* (CCDS4206.1, ensembl) and human *IRF1* (CCDS4155.1, ensembl) sequences. For the P0 collected scRNA-seq, fastq files were mapped with the mouse reference genome, including PBase (https://github.com/awaisj14/Humous), TurboRFP-NLS (https://github.com/awaisj14/Humous) and human *JUNB* (CCDS12280.1, ensembl) sequences for quality control purposes. Counts due to ambient RNA and random barcode swapping were removed from the raw matrix using remove-background function of cellbender^103^ (v0.3.0) with epochs = 150, expected-cells = 2000 for the E16 snRNA-seq dataset and epochs = 50, expected-cells = 6000 for the E16 collected scRNA-seq. The newly generated snRNA-seq and scRNA-seq cellbender count matrix was used for subsequent analysis. scRNA-seq P0 collected dataset was not processed through cellbender. In brief, for the JUNB overexpression E16 collected dataset, cells with nCount_RNA < 25,000, nFeature_RNA > 1,500 and percent.mt < 0.25 were included. For the IRF1/EGR1 overexpression E16 collected dataset, cells with nCount_RNA > 500, nCount_RNA < 20,000, nFeature_RNA > 1,000 and percent.mt < 5 were included. For the P0 collected dataset, nCount_RNA < 30,000, nFeature_RNA > 1,000 and percent.mt < 10 were included. scDblFinder package^104^ (v1.16.0) was used to remove doublets by simulating ncol(sce)/1,000 × 0.008 doublet ratio. A small number of microglia and vasculature cells were removed from the analysis.

#### Cluster definition and annotation

We used Seurat pipeline^105^ (v5.0.1) to normalize and scale the data and performed principal component analysis using the 3,000 most variable features. We performed UMAP dimension reduction using 30 principal components and use the FindNeighbors and FindClusters (resolution = 0.8) functions to define clusters, which we annotated on the basis of their expression of marker genes *Sox2* and *Pax6* for RG, *Eomes* and *Neurog2* for IP and *Neurod2* and *Neurod6* for neurons.

#### Cell cycle scoring

We used the CellCycleScoring function^106^ from Seurat package to define the cell cycle phase of our progenitor populations. Expression of RG human or primate-specific genes in control or JUNB RG at different cell cycle phase was assessed using DotPlot.

#### Differential gene expression

We used the FindMarkers function from Seurat package^105^ with min.pct = 0.2 to find genes differentially expressed between control and JUNB conditions.

#### Lineage barcode analysis

Lineage barcode library fastq files were processed as previously described^101^ with nReads = 10, nUMI = 4 and nHamming = 3 for both datasets using bbmap (v35.85), samtools (v1.15) and umi-tools (v1.1.4). Control and JUNB datasets were processed separately and merged after cloneID assignment. Interneuron-containing clones were removed and the two datasets were balanced to have an equal number of cells for analysis. UpSetR package (1.4.0) was used to assess and plot cell composition within clones.

### Preprocessing of human multiome dataset and base GRN construction

To perform in silico perturbation simulations, the published multiome dataset on PCW21 developing cortical samples was pre-processed from GSE162170 (ref. ^28^) before merging with other datasets. Using scanpy (v1.9.3), an anndata object was generated using available Multiome RNA counts, metadata and cluster names in.txt format with numpy (1.26.2) and pandas (2.1.4). As the varnames were in ensembl_id, it was first to gene_symbol format. The anndata object with unique gene symbols was saved and pre-processed by filtering cells on the basis of max_counts=20000 and min_counts=1500. Genes were filtered using sc.pp.filter_genes with min_counts=1 and the dataset was normalized using sc.pp.normalize_per_cell with key_n_counts = “RNA.counts”. Using canonical markers and rank_genes_groups function in scanpy, RG, IPs and neurons in the dataset were annotated and the rest of the cell types were subsetted out. Next, the Humous curated human datasets were merged with the above dataset. Additional multiomics datasets at from PCW8–10 (ref. ^39^) and PCW11–32 (ref. ^38^) were also pre-processed on the basis of original study annotations (except for IP cluster in PCW8–10 data defined on *EOMES* expression). These datasets, as well as the scRNA-seq human data used here^26–28^, were merged in Seurat and exported to h5ad anndata using SeuratDisk (0.0.0.9021). Cell cycle scoring was performed and top 3000 differentially expressed genes in the anndata were kept. UMAP, PAGA, and ForceAtlas2 graph construction allowed visualization of the three cell types (RG, IP and neuron). One random cell in the RG population of the earliest PCW8–10 time point was selected as the root_cell to perform pseudotime calculation using CellOracle^107^ (v0.14.0) and anndata object was saved with the calculated pseudotime. matplotlib (3.7.0) was used for plotting the data.

Consensus assay for transposase-accessible chromatin (ATAC) peaks in.txt format from datasets^28,38^ were processed to generate a combined bed file denoting the coordinate location of each ATAC peak. Peaks at the TSS of genes were annotated and this processed peak file was saved in.csv format. Next, CellOracle wrapper for gimmemotifs (v0.17.0) was used to generate a base GRN containing motifs in peaks close to 2 kb from all gene promoters. Base GRN containing “motifs to peak coordinates” and the final “transcription factor to peak coordinates” dataframes were saved as.csv files. These were used for motif analysis later.

### Motif analysis of *JUNB* promoter in mouse and human proximal and distal regions

#### Mouse

ATAC peak counts from multiome dataset^108^ generated from E14, E15, and E16 developing cortical samples were used to first score co-accessible peaks using cicero (v1.20.0). Then these co-accessible annotations were imported into the CellOracle wrapper for gimmotifs, which was used to generate a base GRN and final.csv files with defined range of 2 kb from TSS, 10 kb upstream or downstream excluding 2 kb from the TSS, or 100 kb upstream or downstream excluding 2 kb from the TSS.

#### Human

ATAC peak counts from multiomic datasets^28,38^ were subset on the basis of the cells obtained from the anndata processing above. Subsetted cells were then passed through the cicero package to calculate peak co-accessibility separately per dataset. Then the co-accessible peaks from both datasets were combined by merging and averaging similarly co-accessible peak combinations. Once done, same as the mouse motif comparison for mouse *Junb*, a defined range of either 2 kb, 10 kb or 100 kb was used to assess motif composition.

### In silico perturbation of human *JUNB* using CellOracle

Once the RNA and ATAC from the published dataset was pre-processed, GRN construction using the CellOracle (v0.14.0) package was performed through known transcription factor to gene interactions from online databases^109^ (TFLink). To predict dynamic states of expression, imputation of top 3,000 expressed genes was performed according to the CellOracle tutorial, and these imputed values were used for in silico perturbations. A.csv file was generated for each cell-type-specific GRN. Finally, in silico knockout (0 value of imputed_counts) and overexpression (maximum value of imputed_counts) were performed. A custom colour scheme was used to guide the reader with the direction of the arrows following the perturbation.

### Reporting summary

Further information on research design is available in the Nature Portfolio Reporting Summary linked to this article.

## Online content

Any methods, additional references, Nature Portfolio reporting summaries, source data, extended data, supplementary information, acknowledgements, peer review information; details of author contributions and competing interests; and statements of data and code availability are available at https://doi.org/10.1038/s41586-026-10491-x.

## Supplementary information

Supplementary Figure 1 Illustration of gating strategies used for fluorescence-activated single nucleus/cell sorting.

Reporting Summary

Supplementary Table 1 Datasets. Datasets and number of cells used for generating the gene expression landscapes.

Supplementary Table 2 Gene weights and ordinal models. Genes used for the ordinal regression models with their corresponding weight.

Supplementary Table 3 VGG16 clusters. Computer vision-based clustering of gene expression landscapes for genes with patterned profiles. Genes in each cluster are shown by condition.

Supplementary Table 4 Delayed and precocious diff genes in H versus M and GO terms. Ontologies of genes with delayed or precocious expression along the differentiation axis between human and mouse. Abbreviations: RG_DelayDiffH, radial glia genes with delay expression along differentiation in human; RG_PrecoDiffH, RG genes with precocious expression along differentiation in human; IPN_DelayDiffH, intermediate progenitor-neuron genes with delay expression along differentiation in human; IPN_PrecoDiffH, IPN genes with precocious expression along differentiation in human.

Supplementary Table 5 Correlated and anti-correlated genes. Genes with correlated or anti-correlated expression landscapes between human, mouse and human-derived organoid, and their ontologies.

Supplementary Table 6 JUNB targets. JUNB targets and their expression pattern.

Supplementary Table 7 Motif comparisons. Motif comparisons between human and mouse for genes with correlated and anti-correlated expression landscapes. corr, correlation value

Supplementary Table 8 JUNB regulators. JUNB regulators and their expression pattern, with CUT&RUN results for IRF1.

Peer Review File

## Source data

Source Data Fig. 4

Source Data Fig. 5

## Data Availability

Previously published datasets used in this study as follows. Mouse: Gene Expression Omnibus (GEO) accession codes GSE118953 (ref. ^21^), GSE107122 (ref. ^23^), GSE153164 (ref. ^24^) and GSE241429 (ref. ^108^); and Sequence Read Archive accession PRJNA637987 (ref. ^25^). Human: dbGaP phs000989.v3 (ref. ^26^), NeMo nemo:dat-9jd8xw6 (ref. ^27^); GEO accession code GSE162170 (ref. ^28^), NeMo nemo:dat-oiif74w (ref. ^38^) and European Genome–Phenome Archive (EGA) accession code S00001006136 (ref. ^39^). All raw and processed single-cell transcriptomics data generated in this study were submitted under GEO accession code GSE324846. Source data are provided with this paper.

## Code availability

All landscapes of gene expression and correlation values are available on the interactive viewer at https://www.humous.org/. All codes used for data integration, ordinal regression for age and differentiation axis reconstruction, landscapes generation and correlation, computer vision embeddings and landscape characterization were deposited at https://github.com/gomez-L/humous. Pre-processed datasets and intermediate data objects generated through this pipeline can be accessed at https://doi.org/10.17605/OSF.IO/E8GMA. Code used for the subsampling permutations, CellOracle, GRN, CUT&Tag, CUT&RUN and lineage barcode analyses were deposited on https://github.com/awaisj14/humous/.
