# Supplementary material for: Developmental gene expression patterns driving species-specific cortical features
Source: Nature. 2026 May 13;655(8123):694–702. doi: 10.1038/s41586-026-10491-x (PMC13661261; doi:10.1038/s41586-026-10491-x)
Supplement: Supplementary file 1 [file 41586_2026_10491_MOESM11_ESM.pdf]

nature portfolio

Peer Review File

Developmental gene expression patterns driving species-specific cortical features

Corresponding Author: Professor Denis Jabaudon

Any redactions in this file are there to maintain patient confidentiality, the confidentiality of unpublished data, or to remove third-party material.

This file contains all reviewer reports in order by version, followed by all author rebuttals in order by version.

Attachments originally included by the reviewers as part of their assessment can be found at the end of this file.

Version 1:

Reviewer comments:

Referee #1

(Remarks to the Author)
Javed et al. examined the expression patterns of genes across cell types and ages during cortical excitatory neurogenesis. They identified genes that showed differential patterns (or landscapes) across species and culture conditions. They focused on JUNB, which is expressed in radial glia (RG) in humans but in neurons in mouse. Further experiments demonstrated that higher levels of JUNB lead to RG expansion and delayed neuronal differentiation.

The manuscript is clearly written and easy to read. The concept of analyzing gene expression pattern differences across cell types during neural differentiation is very interesting. I also applaud the authors for including both time and differentiation axes to convert gene expression into a two-dimensional landscape, thereby enabling 2D pattern recognition. That said, there are serious issues or limitations in the implementation of this idea. The definition of the age score seems flawed. Moreover, the "time" or "age" factor is not discussed in most of the data interpretations. I believe similar conclusions could be obtained by analyzing only the differentiation axis or using a standard 1D pseudo-time trajectory analysis. To be fair, most of the downstream findings, including JUNB, are based on the differentiation axis, not timepoints, and therefore may still be valid.

1. In the introduction, the authors introduce different RG subtypes, including basal RG and truncated RG (which, by the way, are missing a citation to the original study—PMID: 27657449). However, the subsequent analyses completely ignore RG subtypes. Basal RG and truncated RG are very different in terms of gene expression and behavior. How do the authors address this confounding factor when making comparisons to mice, which lack both subtypes?

2. Related to Extended Data Fig. 2 and all relevant 2D landscape analyses: age scores were calculated using ordinal regression and scaled between 0 and 1. This approach is problematic. It is unlikely that mouse E12 development corresponds precisely to human PCW12, or that E17 maps to PCW24. Organoid timing can also differ significantly from in vivo development. Thus, while comparing gene patterns within a single species or along the differentiation score may be valid, cross-condition or cross-species comparisons assume perfect alignment of developmental stages - an assumption that introduces significant error. This issue is especially concerning when deep learning analyses are applied, as they can detect and amplify subtle artifacts.

3. Related to Fig. 2a and Extended Data Fig. 6: how is entropy calculated? If entropy is measured over the entire image, it may lack interpretability and miss important spatial patterns. Was local or windowed entropy considered? The methods section should include these details. Additionally, the code for this and many other data processing steps is not available.

4. On line 176, the authors write: "Together, these findings reveal two distinct aspects of species-specific cortical development: different temporal organization of developmental programs and divergent evolution of progenitor-specific gene expression." However, there is no real analysis of temporal organization in the paper. I think the authors are referring to differentiation hierarchy. The age axis is largely neglected throughout data interpretation.

5. Similarly, related to Extended Data Fig. 10 and lines 178–188: it is misleading to refer to the cross-cell-type analysis as "temporal gene expression dynamics." The analysis resembles pseudo-time analysis focused on differentiation trajectories rather than real developmental time.

6. On line 214, the correct figure reference should be Fig. 4a, not Fig. 3a.

(Remarks on code availability)

Referee #2

(Remarks to the Author)
I co-reviewed this manuscript with one of the reviewers who provided the listed reports.

(Remarks on code availability)

Referee #3

(Remarks to the Author)
In this work Javed et al. investigated variations in gene expression patterns across species (mouse and human) and contexts (in vivo and in vitro) and how divergent transcriptional regulation of shared genes account for species- and environment-specific differences in cortical development. The authors employed computational and machine vision approaches to identify and compare cyto-temporal gene expression patterns in single-cell RNA sequencing datasets from mouse and human neocortex and human cortical organoids. Among the genes with strong specie-specific expression patterns, they focused on JUNB, and showed its restricted expression in neurons and radial glia (RG) progenitors, in mouse and humans, respectively. By using gain- and loss-of-function experiments in mouse and human organoids they found that JUNB controls RG proliferation through regulation of cell cycle progression, regulating neuronal production timing and neuronal output. By performing GRN reconstruction and in silico transcription factor perturbation analysis, they identified potential upstream regulators and downstream targets of JUNB, which might be responsible for key human cortical development features.

The study uses a broad range of experimental approaches including computer vision models as well as in vivo and in vitro systems to validate the findings. The interactive platform available at www.humous.org is a valuable resource to investigate cyto-temporal patterns of expression of genes of interest in both developmental and disease settings. The premise of projecting cyto-temporal variation in gene expression to a common space is intriguing and has clear potential for comparative analyses. The manuscript is well written and the interpretation of results and discussion is articulated. However, the validity of findings and general conclusions are undermined by several factors, a major one being the developmental timeline differences across conditions.

Major comments

1. Independent prediction models are used for each condition (Hu, Mo, Org). Within each, a cell's position along the 'age' axis is proportional to the age of the tissue sample, relative to other cells in that condition, from 'early' to 'late' corticogenesis. As such, the 'anchor' points that define early and late corticogenesis, represented by 0 and 1 after within-condition normalisation, are critical for the comparison between conditions to be meaningful. In the mouse, the data cover E12-E17, capturing the majority of neurogenesis. However, the human data cover PCW12-24, when neurogenesis begins earlier, at around PCW6. While difficult to estimate, in terms of cortical neurogenesis, E12-17 would more closely correspond to an earlier window in humans of PWC8–17 (Workman et al, J Neuro, 2013; translatingtime.org). This difference is not mitigated by the projection to a [0,1] axis, as any comparison depends upon position along the x axis being comparable across conditions (as is the case with the y-axis, where comparable cell populations were selected from each condition). Application of the same modelling procedure - independent models for each dataset, followed by transformation to occupy the full interval - to two unrelated datasets would allow differences between 'early' and 'late' timing in gene expression to be visualised and compared statistically but the position of cells in each dataset along the x axis would not correspond to each other in any meaningful way. Several analyses depend upon the assumption that position along the x-axis is comparable across conditions (e.g.: Fig 3, Fig S3, Fig S8, Fig S10). In particular, the analysis of precocious and delayed processes will be strongly dependent on the degree of warping required to distribute different time periods in each condition across the same x axis.
The authors may consider some of the following strategies to more thoroughly test this assumption:
- employ a joint ordinal model combining all conditions, taking advantage of known conserved mechanisms to define a truly shared 'age' axis.
- restrict landscapes to periods that overlap between conditions, e.g.: E14-17 and PCW12-17.
- add additional scRNA datasets to provide more coverage across either early neurogenesis in humans, or later neuro/gliogenesis in mouse.

2. This work aims at understanding how cyto-temporal gene expression regulation shapes cortical development across species and environments, however it focuses mostly on neurogenesis (and specifically the excitatory neuron lineage).

Differently from the mouse datasets, the human datasets encompass initial processes of gliogenesis (e.g., OPC generation). How have the authors accounted for that? How this might impact findings regarding the cyto-temporal expression of disease-associated genes and stratification of neurodevelopmental disorders?

3. This study suggests that RG have highly conserved expression patterns across species. This is surprising considering that apical and basal RG were combined together and bRG is prerogative of humans. It would be valuable to discuss the proportions of bRG in the RG partition and include some separate analysis.

4. When integrating scRNAseq datasets, subsampling the original datasets is useful for balancing the number of cells per cell type and time point. However, when it results in a significant reduction in cell numbers, performing multiple permutations of the subsampling to ensure reproducibility is recommended.

5. In figure 4C, JUNB KO organoids seems to have a bigger ventricular zone which is consistent with the prolonged proliferative state of RG. Did the authors detect any difference in organoid size? Images of the entire organoids should be included in Extended Data Figures, in line with the recent guidelines for immunocytochemical characterization of organoids described by Pasca et al. in "A framework for neural organoids, assembloids and transplantation studies, Nature 2015". Information of the iPSC line used and replicates should also be reported.

6. The authors say that comparison between human tissue and organoids revealed differences in cell-cycle related genes. How this might impact the results of cell cycle phase analysis in LOF organoids?

Minor comments

1. The use of “RG” in the text is confusing. In the introduction (L44), “RG” is used to refer to apical radial glia, but in L67 and figure 1a it is implicit that it refers to both apical and basal RG. Only later (L92) the authors specify that Hu bRG were considered as RG for the analysis.

2. Figure 1a, left: the X axis label in the schematic of cortical growth is not consistent with what reported in the text and Extended table 1.

3. Each ordinal model is fitted twice, once with all genes, then again using only genes with the highest (absolute) weights. Only the second is cross-validated. It is not clear why two steps were used – an improvement in model fit? More interpretable weights? In prediction models, this procedure (feature selection outside of a cross validation loop) is likely to result in overfitting, particularly when there are a (likely) substantial number of colinear variables and may explain the lack of correlation between age model weights across conditions (Fig S3). The authors should consider: completing the feature selection within the cross-validation loop, reporting model fits/prediction accuracy averaged over folds, or using a simpler 1-step, cross validated model.

4. Were landscape comparisons corrected for differences in cell density across conditions? In all conditions there are sparsely populated areas in each landscape (Fig S3). In these regions, the k-NN process – averaging over the nearest 100 cells – may average expression over larger (and therefore more dissimilar) areas, spreading signal along each axis.

5. VGG16 was used to generate image features – 25,0888 per landscape from 59,536 pixels (244x244). The top 2000 were then used for clustering. How does this compare to simply clustering the landscapes based on the top 2000 pixels (i.e.: without VGG embedding)?

6. In several of the low expression landscapes, circular artefacts are clearly present – potentially from the k-NN averaging? Have the authors considered applying smoothing, or outlier detection to mitigate these artefacts?

7. It would be valuable to perform the in-silico knockout with CellOracle using additional datasets. Workarounds allow its use without ATAC-seq data.

8. The integration of different datasets should be presented more clearly, by showing separate UMAPs colored by batch and relevant integration metrics.

9. The code provided on GitHub demonstrates only the application of CellOracle but does not include the full implementation of the workflow, which is important for reproducibility and reuse.

10. Minor adjustments
L467: ensure numbers in each differentiation group match that shown in Table S1
Figure 1a: Add a schematic for organoids too
Line 89: reference 52 (OrgH dataset – ID Ck19) is missing and its title in Suppl Table 1 is uncomplete
L230: The suggestion that the JUNB promoter is poised for activation in mouse RG is a bit too speculative. Other explanations are possible.
L 83: Cortical “neurons” should be replaced with “cells”?
L87: specify number of cells after balancing
L103: should be low SD – high entropy?
L474: 750 total? (5 per age group, 3 diff groups)

L476: specify total number of genes
L572: ‘lower than -0.5’ rather than ‘smaller’ for clarity
Extended Figure 2a – legend, remove reference to Right: plot
Extended Figure 9a– legend, ‘fonction’

(Remarks on code availability)
The code provided on GitHub demonstrates only the application of CellOracle but does not include the full implementation of the workflow.

Referee #4

(Remarks to the Author)
In the study titled “Developmental gene expression patterns driving species-specific cortical features”, Javed et al. analyze developmental single-cell transcriptomes from mouse, human, and human organoids to identify conserved and divergent gene expression landscapes, highlighting the transcription factor JUNB. They combine CNN-based pattern recognition, correlation analysis, and functional experiments to propose that JUNB regulates progenitor proliferation and contributes to human-specific cortical features. The study introduces a novel trajectory-analysis method to extract new insights from existing data and validates JUNB as a candidate gene influencing brain expansion. However, the claim that JUNB is human-specific is misleading since it is potentially conserved in macaque, suggesting a primate- or potentially mouse-specialization. Similarly, disease enrichment claims are speculative and potentially misleading. In sum, while the resource and functional data are valuable, the manuscript suffers from overinterpretation and lacks rigor in its evolutionary and disease relevance claims.

Major Comments:

- The authors report greater expression patterning in mouse than human (Fig. 2a), but do not sufficiently control for quality differences in the source datasets. Human samples may suffer from greater tissue degradation or donor variability, confounding conclusions about species-specific gene patterning. A more rigorous cross-species normalization or benchmarking is needed to support these claims.

- The observed divergence in IP gene expression (Fig. 2d–e) could be driven by sampling depth or expression noise. The authors should assess whether low gene detection in human IPs or doublets contribute to these apparent differences. Prior literature should be cited to clarify if extended expression of cell cycle genes in human IPs is expected or novel.

- The enrichment of disease-associated genes in IPs (Fig. 2f) is largely based on proximity to GWAS hits. As the authors note, many risk variants are regulatory and may not act during development. Without supporting chromatin accessibility (e.g., scATAC-seq) or enhancer maps, the assignment of developmental cell-type relevance should be de-emphasized.

- JUNB is highlighted as a species-specific regulator, yet its expression in macaque organoids (ED Fig. 12b) contradicts the claim that it is human-specific. The analysis of enhancer accessibility focuses on the JUNB promoter (ED Fig. 12e), but regulation in RG is more likely mediated by distal enhancers. Why were putative enhancers not analyzed for TF enrichment with published human development ATAC-seq datasets, especially since CellOracle predicts regulators like FOS and EGR1?

- JUNB gain-of-function in mouse RG prolongs progenitor proliferation, consistent with previous reports that RG self-renewal regulates cortical expansion. However, this is not sufficient to establish JUNB as a central driver of superficial layer neuron expansion since many other genes could be involved.

- The in silico perturbation using Cell Oracle (Fig. 4i) is suggestive but lacks validation. scATAC-seq or ChIP-seq data from human RG would be more appropriate to define JUNB targets and upstream regulators.

- The analysis of human vs. organoid expression patterns (Fig. 3a, ED Fig. 7f) is relatively underdeveloped. Many genes conserved in human and mouse are poorly modeled in organoids. This is a key point for improving organoid utility as an evolutionary model and deserves deeper exploration.

Minor Comments:

Fig. 1b – Neuronal organization by age is less clear in organoids. Authors should quantify this effect using trajectory distances or pseudotime dispersion.

Fig. 2a – The GO analysis relies on a thresholded set of "patterned" genes. Could human genes involved in synapse organization have narrowly missed this cutoff?

Fig. 2e – Clarify what is compared in the correlation analyses. Do all IP clusters (c11, c12, c13) have similarly low cross-species correlations?

ED Fig. 10 – The timing differences observed are intriguing. However, the authors should discuss whether these are

consistent with prior interspecies pseudotime studies.

(Remarks on code availability)

Referee #5

(Remarks to the Author)
I co-reviewed this manuscript with one of the reviewers who provided the listed reports.

(Remarks on code availability)

Referee #6

(Remarks to the Author)
I co-reviewed this manuscript with one of the reviewers who provided the listed reports.

(Remarks on code availability)

Version 2:

Reviewer comments:

Referee #1

(Remarks to the Author)
The authors have addressed concerns regarding alignment of developmental age across species, as well as many other points raised in the first round of review. However, in Extended Data Fig. 5d, the dynamic time warping (DTW) analysis appears to have been performed using the original dataset starting from PCW12 onward, rather than the full extended dataset. It would be helpful to include data from PCW8–12 in humans and E10–11 in mice, and to assess whether DTW still aligns E12 in mouse with PCW12 in human when these earlier developmental stages are incorporated.

A related concern pertains to the alignment of the differentiation axis, which relies on accurate annotation of radial glia (RG), intermediate progenitors (IP), and neurons. While marker-based annotation is discussed, the differentiation axis itself critically depends on the fidelity of these cell-type assignments. In Extended Data Fig. 1a and 1b, although nearly all mouse IPs are EOMES-positive, a substantial fraction of human cells annotated as IP appear to be EOMES-negative, as also reflected in the violin plots in Extended Data Fig. 7a,c,d (specifically the IP1 cluster). This raises the possibility that a subset of human RGs may be misannotated as IPs.

Such potential misassignment could influence downstream analyses, including the reported differences in IPC gene expression patterns between human and mouse. To address this concern, it may be helpful for the authors to consider label transfer or data integration–based approaches to define RG, IP, and neuron populations across species or platforms. Ideally, these approaches would yield results consistent with the current annotations; if not, some conclusions may need to be revisited.

(Remarks on code availability)

Referee #2

(Remarks to the Author)
I co-reviewed this manuscript with one of the reviewers who provided the listed reports.

(Remarks on code availability)

Referee #3

(Remarks to the Author)
I appreciate the authors’ efforts in considering my comments.

A major concern, also raised by other reviewers, was related to the age alignment across species (Comment #1). The additional information provided by the authors improves this aspect, both in the discussion of how they chose the temporal landmarks and the additional validation and extra data. However, this is a critical aspect upon which major analyses are based and should be addressed in detail and supported by analysis in the main text (now it is discussed in Extended Data Figures and Methods). While the distinction between L6 and L2/3 neurogenesis mitigate some of the concerns, the results

do not really provide a “comprehensive insight into cortical cell-type specific and temporal patterns of gene expression across species and context”, as claimed by the authors in the discussion.

The distinction between basal radial glia (bRG) and truncated radial glia (tRG)(Comment #3) deserves more consideration. These RG subtypes are clearly transcriptionally distinct as shown in Ext Fig 7a-c. A more thorough analysis would strengthen the manuscript.

The organoid work is not fully convincing. The two images in Extended Data Figure 14 show considerable variability in organoid morphology, suggesting that a larger sample size is needed to confirm the findings. Furthermore, several inconsistencies in the organoid work are evident. In the Methods section, the authors state that “In organoids, we focused on stages in vitro where RG, IP, and N were found in substantial numbers in the original datasets, following Uzquiano et al Cell 2022 (ref 82)”. However, this reference dataset was generated using a guided organoid model, which differs from the unguided model used by the authors (Giandomenico 2021, Lancaster 2014). Therefore, it cannot serve as a valid reference. This discrepancy reinforces concerns about inappropriate correspondence between timepoints across datasets (Comment#1), extending it to the in vivo and in vitro analysis. Additionally, organoids used for the Cut&Tag and Cut&Run experiments were produced using the Stem Cell Technology kit, which, although based on the Giandomenico and Lancaster protocols, represents a distinct method.

(Remarks on code availability)

Referee #4

(Remarks to the Author)
The authors have addressed my comments, particularly making it clear that the observed features are "human-like" not necessarily "human-specific". I support publication.

(Remarks on code availability)

Referee #5

(Remarks to the Author)
I co-reviewed this manuscript with one of the reviewers who provided the listed reports.

(Remarks on code availability)
They appear to have archived the code and data objects, the analysis scripts look fine. It would take a lot of work to apply their method to a new dataset, but they're not really trying to publish a tool.

Version 3:

Reviewer comments:

Referee #1

(Remarks to the Author)
I am satisfied that my concerns have been addressed.

(Remarks on code availability)

Referee #2

(Remarks to the Author)
I co-reviewed this manuscript with one of the reviewers who provided the listed reports.

(Remarks on code availability)

Referee #3

(Remarks to the Author)
The authors have addressed my previous comment in their revised version of the manuscript. I do not have any further requests.

(Remarks on code availability)
My collaborator and co-reviewer reviewed the code and confirmed the scripts look fine.

Referee #6

(Remarks to the Author)
I co-reviewed this manuscript with one of the reviewers who provided the listed reports.

(Remarks on code availability)

Open Access This Peer Review File is licensed under a Creative Commons Attribution 4.0 International License, which permits use, sharing, adaptation, distribution and reproduction in any medium or format, as long as you give appropriate credit to the original author(s) and the source, provide a link to the Creative Commons license, and indicate if changes were made.
In cases where reviewers are anonymous, credit should be given to 'Anonymous Referee' and the source.
The images or other third party material in this Peer Review File are included in the article's Creative Commons license, unless indicated otherwise in a credit line to the material. If material is not included in the article's Creative Commons license and your intended use is not permitted by statutory regulation or exceeds the permitted use, you will need to obtain permission directly from the copyright holder.
To view a copy of this license, visit https://creativecommons.org/licenses/by/4.0/

Referee #1 (Remarks to the Author):

Javed et al. examined the expression patterns of genes across cell types and ages during cortical excitatory neurogenesis. They identified genes that showed differential patterns (or landscapes) across species and culture conditions. They focused on JUNB, which is expressed in radial glia (RG) in humans but in neurons in mouse. Further experiments demonstrated that higher levels of JUNB lead to RG expansion and delayed neuronal differentiation. The manuscript is clearly written and easy to read. The concept of analyzing gene expression pattern differences across cell types during neural differentiation is very interesting. I also applaud the authors for including both time and differentiation axes to convert gene expression into a two-dimensional landscape, thereby enabling 2D pattern recognition.

That said, there are serious issues or limitations in the implementation of this idea.

The definition of the age score seems flawed. Moreover, the "time" or "age" factor is not discussed in most of the data interpretations. I believe similar conclusions could be obtained by analyzing only the differentiation axis or using a standard 1D pseudo-time trajectory analysis. To be fair, most of the downstream findings, including JUNB, are based on the differentiation axis, not timepoints, and therefore may still be valid.

We thank the reviewer for their assessment of our manuscript. In the answers below, we address the points raised through updates to the text and additional analyses and validations.

1. In the introduction, the authors introduce different RG subtypes, including basal RG and truncated RG (which, by the way, are missing a citation to the original study—PMID: 27657449). However, the subsequent analyses completely ignore RG subtypes. Basal RG and truncated RG are very different in terms of gene expression and behavior. How do the authors address this confounding factor when making comparisons to mice, which lack both subtypes?

To make comparisons between Hu and Mo RG, we defined RG as $SOX2^{+}$ $TBR2^{-}$ cells, which encompasses all Hu RG subtypes. **This is now made more explicit in the methods** (p.19, l.547-548).

Following the reviewer's suggestion to seek for RG subtypes in humans, we performed high-resolution clustering of Hu progenitors and annotated them using established markers for basal RG (*HOPX*, *FAM107A*, *TNC*), and truncated RG (*CRYAB*), along with annotations from Nowakowski et al., 2017. This approach revealed that basal RG (absent in Mo) increase with time in Hu, while truncated RG could not be identified on transcriptional bases alone. **These new data are now presented in the new Extended Data Fig. 7 and discussed in the text** (p.5, l.172-178).

The reference to the original study on RG diversity in humans has now been added (p.5, l.173, Nowakowski et al., 2016), as well as recently published relevant datasets and commentaries (Keefe et al., 2025; Nowakowski et al., 2025; Wang et al., 2025). The temporal unfolding of basal RG emergence and less sharply defined molecular identity of truncated RG is consistent with previous studies, which have also been added (Fietz et al., 2010; Pollen et al., 2015; Wang et al., 2025).

2. Related to Extended Data Fig. 2 and all relevant 2D landscape analyses: age scores were calculated using ordinal regression and scaled between 0 and 1. This approach is problematic. It is unlikely that mouse E12 development corresponds precisely to human PCW12, or that E17 maps to PCW24. Organoid timing can also differ significantly from in vivo development. Thus, while comparing gene patterns within a single species or along the differentiation score may

be valid, cross-condition or cross-species comparisons assume perfect alignment of developmental stages - an assumption that introduces significant error. This issue is especially concerning when deep learning analyses are applied, as they can detect and amplify subtle artifacts.

We thank the reviewer for raising these points. Comparing developmental time across species requires assumptions about which developmental landmarks correspond, and about the rates of maturation between these landmarks. To address the points raised, **we have clarified our alignment strategy and added several analyses to cross-validate it.**

We aligned developmental stages using two well-established cortical landmarks: L6 generation (PCW10-13 in human; E11-12 in mouse) and L2/3 generation (PCW20-24 in human; E16 in mouse). This strategy is now explicitly described in the **methods** (p.18, l.508-518) and better illustrated in **Fig. 1a**. We agree, however, that these developmental landmarks span several days and that misalignment could distort cross-species comparisons. To exclude this possibility, we performed **three independent validations:**

(1) If temporal anchors were misaligned, genes expressed early in one species should consistently appear shifted later in the other. However, as shown in **the new Extended Data Fig. 10d,e**, early human genes tend to map to early or non-specific mouse genes (and vice-versa for late genes), with no directional bias.

(2) **We expanded the dataset to include earlier and later developmental stages** (human PCW8-12 and 25-32; mouse E10-11, E18, E20) (van Bruggen et al., 2022; Wang et al., 2025). If our anchor points were critically sensitive to precise timing, some of these flanking stages should match better across species. This was not the case, as the prediction curve remains essentially flat across the additional earlier time points, indicating that even a potential misalignment would have only minimal effect on the model. **These new results are shown in the new Extended Data Fig. 5c**.

(3) **To quantify how well each human stage aligns to each mouse stage, we performed dynamic time warping** (DTW) analysis using dtw package (Giorgino, 2009), which uses Euclidean distances from the top 30 principal components to compare stages. This approach allowed us to examine the full warping path and, revealed clear and biologically coherent correspondences (*e.g.,* Hu 12 PCW → Mo E12–E13; Hu 15–16 and 17–18 PCW → Mo E14; Hu 20–21 PCW → Mo E14–E15; Hu 23–24 PCW → Mo E15–E16). Quantification using centroid ± interquartile range plots show precise early and late matches and only slight mid-gestation broadening, supporting the cross-species developmental relationships used here. **These new results are shown in the new Extended Data Fig. 5d.**

For organoids, aligning developmental time across datasets generated with different protocols is indeed more challenging. To minimize this uncertainty, we restricted analyses to stages at which RG–IP–N populations co-exist in proportions consistent with *in vivo* tissue, following Uzquiano et al., 2022. **This is now clarified in the methods** (p.18, l.518-520).

In addition to the points highlighted above, a short note acknowledging the limitations of age alignment across species / contexts has been added to the **methods** (p.18, l.508-510).

Finally, regarding the concern that deep learning models may amplify subtle temporal artifacts, our approach includes two safeguards. We pre-filter genes using entropy and variance thresholds (**methods**, p.21), ensuring that low-information or noisy genes do not enter the model. Moreover, the features extracted by the computer-vision model are predominantly driven by differentiation state rather than absolute age, as indicated by the integrated UMAP in Fig. 2, where cells organize by type and state rather than fine temporal differences. Because within-cell-type age shifts are far smaller than differences across cell types, the model is

intrinsically robust to minor temporal misalignments. **These clarifications are now included in the methods** (p.22, l.658-661).

Together, these analyses support that the alignment captures genuine biological correspondence rather than artifactually imposed similarity, while acknowledging the intrinsic limitations of cross-species temporal alignments.

3. Related to Fig. 2a and Extended Data Fig. 6: how is entropy calculated? If entropy is measured over the entire image, it may lack interpretability and miss important spatial patterns. Was local or windowed entropy considered? The methods section should include these details. Additionally, the code for this and many other data processing steps is not available.

We apologize for the lack of clarity. In our analysis, entropy was not used to detect spatial patterns *per se* – they are instead captured by the computer-vision model – but to classify genes as lowly, ubiquitously, or non-specifically expressed. For this purpose, we computed Cauchy–Schwarz (CS) entropy using the R entropy package on the full 0–1 scaled landscape arrays. Because the goal was to quantify overall specificity rather than local spatial structure, windowed or local entropy measures were not required.

This rationale, together with the calculation procedure and its use alongside landscape standard deviation (Extended Data Fig. 8), is **now clarified in the methods** (p.21, l.633-640). In addition, we now provide links to all code used for this and other processing steps in the **Code and Data Availability sections** (p.34).

4. On line 176, the authors write: "Together, these findings reveal two distinct aspects of species-specific cortical development: different temporal organization of developmental programs and divergent evolution of progenitor-specific gene expression." However, there is no real analysis of temporal organization in the paper. I think the authors are referring to differentiation hierarchy. The age axis is largely neglected throughout data interpretation.

Agreed, we have modified this paragraph and made clear difference between **temporal *vs.* differentiation hierarchies** (p.4-5, l.146-149; l.152-157; l.161-178).

Regarding the age *vs.* differentiation axis focus, we concentrate on the latter because differentiation is the principal driver of gene expression variability. Indeed, even within species, cell type identity is better predicted than collection age in the ordinal models (**new Extended Data Figs. 4,5**). **This is now clarified this in the results** (p.4-5, l.146-149).

Although rarer, genes with temporal patterns within cell types do exist and correspond to meaningful developmental processes, for example highlighting early proliferative intermediate progenitors in human, late emergence of basal radial glia (see point 1) and timing of the gliogenic switch, which **we are now illustrated in more detail** (see p.5, l.161-178 & p.7, l.231-234 and **Extended Data Fig. 7**).

5. Similarly, related to Extended Data Fig. 10 and lines 178–188: it is misleading to refer to the cross-cell-type analysis as "temporal gene expression dynamics." The analysis resembles pseudo-time analysis focused on differentiation trajectories rather than real developmental time.

Agreed, this is now corrected; we now refer to **differentiation** trajectories (p.6, l.204-206).

6. On line 214, the correct figure reference should be Fig. 4a, not Fig. 3a.

Here we refer to the correlation values of corresponding *JUNB* landscapes between Hu and Mo, which are shown correctly shown in **Fig. 3a**, not **Fig. 4a**. We have updated the text for clarity.

Referee #2 (Remarks to the Author):

I co-reviewed this manuscript with one of the reviewers who provided the listed reports.

Referee #3 (Remarks to the Author):

In this work Javed et al. investigated variations in gene expression patterns across species (mouse and human) and contexts (in vivo and in vitro) and how divergent transcriptional regulation of shared genes account for species- and environment-specific differences in cortical development. The authors employed computational and machine vision approaches to identify and compare cyto-temporal gene expression patterns in single-cell RNA sequencing datasets from mouse and human neocortex and human cortical organoids. Among the genes with strong specie-specific expression patterns, they focused on JUNB, and showed its restricted expression in neurons and radial glia (RG) progenitors, in mouse and humans, respectively. By using gain- and loss-of-function experiments in mouse and human organoids they found that JUNB controls RG proliferation through regulation of cell cycle progression, regulating neuronal production timing and neuronal output. By performing GRN reconstruction and in silico transcription factor perturbation analysis, they identified potential upstream regulators and downstream targets of JUNB, which might be responsible for key human cortical development features.

The study uses a broad range of experimental approaches including computer vision models as well as in vivo and in vitro systems to validate the findings. The interactive platform available at www.humous.org is a valuable resource to investigate cyto-temporal patterns of expression of genes of interest in both developmental and disease settings. The premise of projecting cyto-temporal variation in gene expression to a common space is intriguing and has clear potential for comparative analyses. The manuscript is well written and the interpretation of results and discussion is articulated.

However, the validity of findings and general conclusions are undermined by several factors, a major one being the developmental timeline differences across conditions.

We thank the reviewer for their constructive comments and provide new analyses to better justify the aspects pertaining to the timeline.

Major comments:

1. Independent prediction models are used for each condition (Hu, Mo, Org). Within each, a cell's position along the 'age' axis is proportional to the age of the tissue sample, relative to other cells in that condition, from 'early' to 'late' corticogenesis. As such, the 'anchor' points that define early and late corticogenesis, represented by 0 and 1 after within-condition normalisation, are critical for the comparison between conditions to be meaningful. In the mouse, the data cover E12-E17, capturing the majority of neurogenesis.

However, the human data cover PCW12-24, when neurogenesis begins earlier, at around PCW6. While difficult to estimate, in terms of cortical neurogenesis, E12-17 would more closely correspond to an earlier window in humans of PWC8–17 (Workman et al, J Neuro, 2013; translatingtime.org). This difference is not mitigated by the projection to a [0,1] axis, as any comparison depends upon position along the x axis being comparable across conditions (as is the case with the y-axis, where comparable cell populations were selected from each condition).

Application of the same modelling procedure - independent models for each dataset, followed by transformation to occupy the full interval - to two unrelated datasets would allow differences between 'early' and 'late' timing in gene expression to be visualised and compared statistically but the position of cells in each dataset along the x axis would not correspond to each other in any meaningful way.

Several analyses depend upon the assumption that position along the x-axis is comparable across conditions (e.g.: Fig 3, Fig S3, Fig S8, Fig S10). In particular, the analysis of precocious and delayed processes will be strongly dependent on the degree of warping required to distribute different time periods in each condition across the same x axis.

The authors may consider some of the following strategies to more thoroughly test this assumption:
- employ a joint ordinal model combining all conditions, taking advantage of known conserved mechanisms to define a truly shared 'age' axis.

- restrict landscapes to periods that overlap between conditions, e.g.: E14-17 and PCW12-17.

- add additional scRNA datasets to provide more coverage across either early neurogenesis in humans, or later neuro/gliogenesis in mouse.

The reviewer's remarks refer to (1) the comparability of positions along the age axis across datasets, (2) possible strategies to establish a shared developmental timescale. We address each of these two points below.

1. Anchoring of starting and ending points:

We aligned developmental stages using two well-established cortical landmarks: L6 generation (PCW10-13 in human; E11-12 in mouse) and L2/3 generation (PCW20-24 in human; E16 in mouse). This strategy is now explicitly described in the **methods** (p.18, l.508-518) and better illustrated in **Fig. 1a**. We agree, however, that these developmental landmarks span several days and that misalignment could distort cross-species comparisons. To exclude this possibility, we performed three independent validations:

(1) If temporal anchors were misaligned, genes expressed early in one species should consistently appear shifted later in the other. However, as shown in **the new Extended Data Fig. 10d,e**, early human genes tend to map to early or non-specific mouse genes (and vice-versa for late genes), with no directional bias.

(2) **We expanded the dataset to include earlier and later developmental stages** (human PCW8-12 and 25-32; mouse E10-11, E18, E20) (van Bruggen et al., 2022; Wang et al., 2025). If our anchor points were critically sensitive to precise timing, some of these flanking stages should match better across species. This was not the case, as the prediction curve remains essentially flat across the additional earlier time points, indicating that even a potential misalignment would have only minimal effect on the model. **These new results are shown in the new Extended Data Fig. 5c**.

(3) **To quantify how well each human stage aligns to each mouse stage, we performed dynamic time warping** (DTW) analysis using dtw package (Giorgino, 2009), which uses Euclidean distances from the top 30 principal components to compare stages. This approach allowed us to examine the full warping path and, revealed clear and biologically coherent correspondences (*e.g.,* Hu 12 PCW → Mo E12–E13; Hu 15–16 and 17–18 PCW → Mo E14; Hu 20–21 PCW → Mo E14–E15; Hu 23–24 PCW → Mo E15–E16). Quantification using centroid ± interquartile range plots show precise early and late matches and only slight mid-gestation broadening, supporting the cross-species developmental relationships used here. **These new results are shown in the new Extended Data Fig. 5d.**

2. Application of proposed strategies:

<u>*Employ a joint ordinal model combining conditions.*</u> We implemented the suggested joint ordinal model, which performed markedly worse than our condition-specific models (joint model: mean rho = 0.34 *vs*. condition-specific: mean rho = 0.65) (**Figure for reviewer 2**). **This likely reflects the fact that cell types and developmental programs mature at different rates** across species and conditions, such that pooling them obscures these cell-type–specific trajectories. Consistent with this, the weights of genes used to predict ages in the different conditions are poorly correlated (**Extended Data Fig. 3a**), indicating that age-encoding programs are highly species- and context-dependent. Together, these observations support our choice of condition-specific models. **A note to this effect has been added in the methods** (p.19-20, l.571-573).

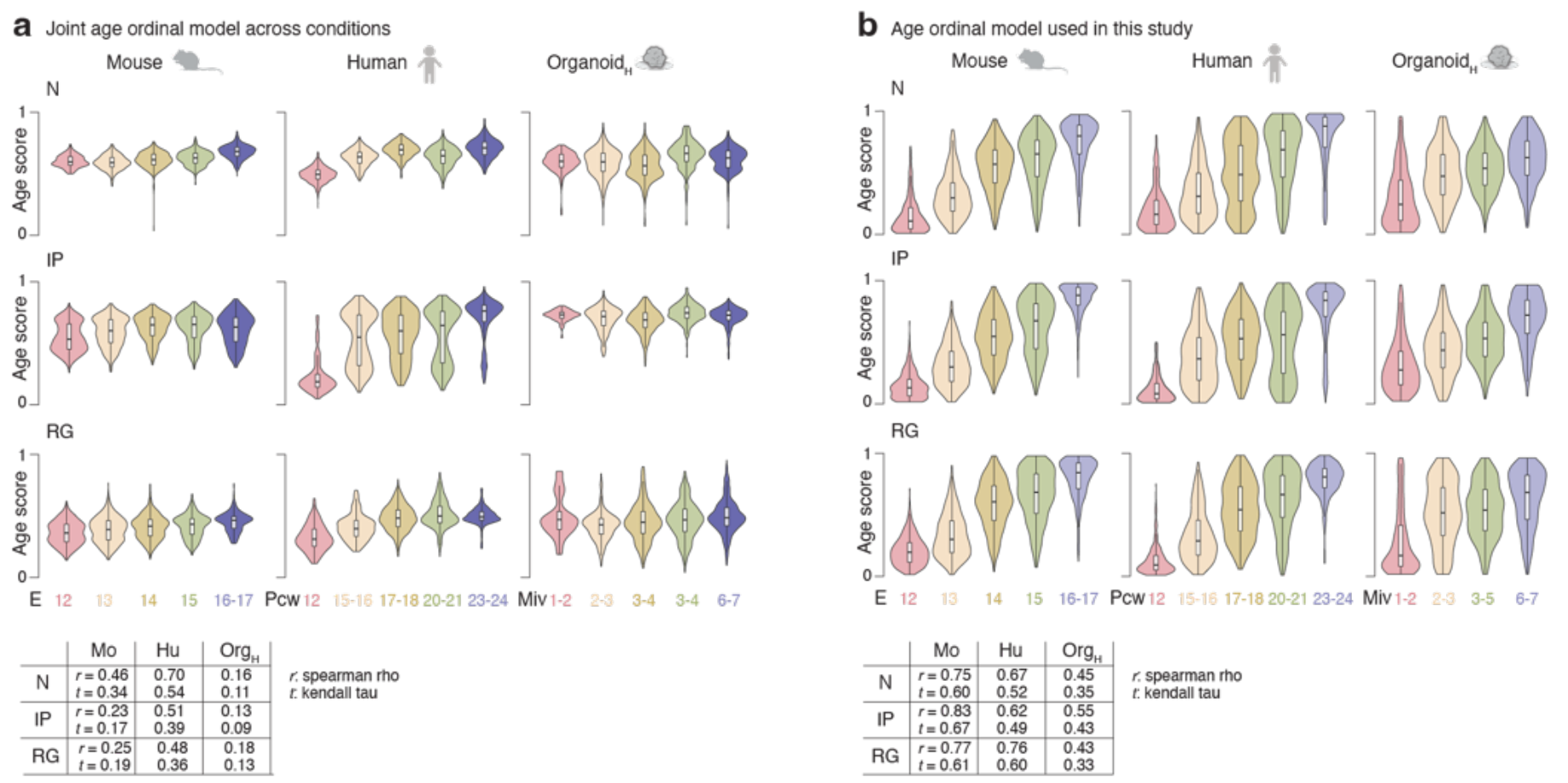

Figure for reviewer 2 | Joint ordinal model across conditions does not predict actual age accurately. Comparison of age prediction scores and actual ages at collection using a joint ordinal model (**a**) or species-specific ordinal model (used in the study) (**b**).

<u>*Restrict landscapes to period that overlap between conditions.*</u> We now clarify that the start and end points are L6 generation to L2/3 generation, respectively, as detailed in **point 1** above.

<u>*Add additional datasets.*</u> We integrated other datasets encompassing earlier and later times of collection (Hu: PCW8-12 and 25-32; van Bruggen et al., 2022; Wang et al., 2025; Mo: E10-11, E18, P1; Di Bella et al., 2021), as detailed in **point 1 above** (**Extended Data Fig. 1d**), which showed no better match than the datasets used in this study.

Together, these findings confirm that condition-specific models and the chosen temporal windows offer the most robust and biologically meaningful comparison across species and conditions.

2. This work aims at understanding how cyto-temporal gene expression regulation shapes cortical development across species and environments, however it focuses mostly on neurogenesis (and specifically the excitatory neuron lineage). Differently from the mouse

datasets, the human datasets encompass initial processes of gliogenesis (e.g., OPC generation). How have the authors accounted for that? How this might impact findings regarding the cyto-temporal expression of disease-associated genes and stratification of neurodevelopmental disorders?

Following the reviewer's suggestion, we sought for markers of gliogenesis (including OPC) in the Hu and Mo datasets. This showed that the E16-E17 mouse datasets also contain gliogenic radial glia, which are reflected by the expression of *Clu*, *Sparcl1* and *Bcan* (**new Extended Data Fig. 7**), consistent with known timing of gliogenesis in mouse (Zhou et al., 2025). Therefore, **RG at late timepoints have a gliogenic potential in both Hu and Mo**. This is now mentioned (p.3, l.102-104). The paragraphs on disease stratification have been significantly reduced (p.3, l.79-80; p.10, l.342-345).

3. This study suggests that RG have highly conserved expression patterns across species. This is surprising considering that apical and basal RG were combined together and bRG is prerogative of humans. It would be valuable to discuss the proportions of bRG in the RG partition and include some separate analysis.

We apologize for the confusing phrasing as we did not mean to imply that RG are highly conserved across species, but that they were more conserved than IP are, *i.e.,* IP diverge more in their transcriptional programs. **We rephrased to avoid the misleading impression that RG are similar between species** (p.5, l.161-178).

Building on the reviewer's suggestion, however, we sought for RG subtypes in humans by performing high-resolution clustering of Hu progenitors and annotated them using established markers for basal RG (*HOPX*, *FAM107A*, *TNC*), and truncated RG (*CRYAB*), along with annotations from Nowakowski et al., 2017. This approach revealed that basal RG (absent in Mo) increase with time in Hu, while truncated RG could not be identified on transcriptional bases alone. **These new data are now presented in the new Extended Data Fig. 7 and discussed in the text** (p.5, l.172-178).

4. When integrating scRNAseq datasets, subsampling the original datasets is useful for balancing the number of cells per cell type and time point. However, when it results in a significant reduction in cell numbers, performing multiple permutations of the subsampling to ensure reproducibility is recommended.

Following the reviewer's recommendation, we performed multiple subsampling of the original dataset, and generated landscapes, which were then correlated gene per gene to the original landscapes. **This analysis showed that top gene weights were correlated in the models, and that landscape patterns were correlated,** with 80% (Mo), 75% (Hu) and 53% (Org_H) of genes being highly correlated ($r > 0.5$). The lower proportion of highly correlated genes in Org_H likely reflects greater variability between organoid protocols and the intrinsic heterogeneity of organoid systems, which impose additional noise on gene-level correlations. This additional quality control is now presented in the **methods** (p.22, *Robustness of gene expression landscapes*) and in the **new Extended Data Fig. 6**.

5. In figure 4C, JUNB KO organoids seems to have a bigger ventricular zone which is consistent with the prolonged proliferative state of RG. Did the authors detect any difference in organoid size? Images of the entire organoids should be included in Extended Data Figures, in line with the recent guidelines for immunocytochemical characterization of organoids described by Pasca et al. in "A framework for neural organoids, assembloids and

transplantation studies, Nature 2015". Information of the iPSC line used and replicates should also be reported.

We quantified VZ thickness in JUNB KO organoids and did not find a significant difference compared to control (**new Extended Data Fig. 14c)**.

We have added representative images of the entire organoids (**new Extended Data Fig. 14b**).

Information about the iPSC lines is provided in the **methods** (p.25, *iPSCs lines and maintenance*) and details about replicates are now described in corresponding figure legends.

6. The authors say that comparison between human tissue and organoids revealed differences in cell-cycle related genes. How this might impact the results of cell cycle phase analysis in LOF organoids?

The differences in cell-cycle–related genes that we report between human tissue and organoids occur in intermediate progenitors (IP) (**Fig. 3**), whereas our JUNB loss-of-function analysis focuses on RG. Because the observed transcriptional divergence is specific to IP, it does not directly affect our interpretation of RG cell-cycle behavior in the LOF organoids. To address this potential concern more broadly, we validated JUNB function not only in human organoids but also in mouse tissue, JUNB loss-of-function in organoids accelerates RG differentiation toward a neuronal fate (Fig. 4c), which is consistent with the phenotype observed in mouse following gain-of-function. **The text has been updated to clarify this** (p.8, l.261).

Minor comments

1. The use of "RG" in the text is confusing. In the introduction (L44), "RG" is used to refer to apical radial glia, but in L67 and figure 1a it is implicit that it refers to both apical and basal RG. Only later (L92) the authors specify that Hu bRG were considered as RG for the analysis.

Agreed. We now refer to RG for radial glia (comprising both aRG and bRG) (p.2, l.50).

2. Figure 1a, left: the X axis label in the schematic of cortical growth is not consistent with what reported in the text and Extended table 1.

We apologize for the confusion. We modified the schematic and corresponding text for clarity (p.3, l.95).

3. Each ordinal model is fitted twice, once with all genes, then again using only genes with the highest (absolute) weights. Only the second is cross-validated. It is not clear why two steps were used – an improvement in model fit? More interpretable weights? In prediction models, this procedure (feature selection outside of a cross validation loop) is likely to result in overfitting, particularly when there are a (likely) substantial number of colinear variables and may explain the lack of correlation between age model weights across conditions (Fig S3).

The authors should consider: completing the feature selection within the cross-validation loop, reporting model fits/prediction accuracy averaged over folds, or using a simpler 1-step, cross validated model.

We apologize for the unclear phrasing; feature selection was indeed completed within the cross-validation loop, as requested.

Both fitting steps are done sequentially during the training process, without considering the test data. We used a **two-step modeling** (instead of 1) so that:

- The **first step is used as a feature selection step** to produce a sparse model with a single, stable, and ranked set of genes associated with the ordinal axis. This separation is essential because feature selection in high-dimensional settings is known to be unstable (Kalousis et al., 2007) and performing it independently within each resampling fold would yield different gene sets across folds.
- The **second fitting step is then performed in this reduced, biologically meaningful feature space**. Although dimensionality is substantially reduced, the model retains sufficient flexibility to **re-estimate the relative importance of the 50 axis-representative genes** for each subset of cells. The prediction scores for each cell are calculated once with cross-validation during this second fitting.

We mask predictions according to the cross-validation fold identifiers, ensuring that **every ordinal-score prediction is generated by a model trained on an independent subset of cells, preventing overfitting**. This design yields true out-of-sample cell-level predictions, without calculating repeated predictions for the same cell.

Regarding the suggestion of using classical nested cross-validation or one-step CV procedures: these approaches are not optimal for our study as they would produce fold-specific gene sets, precluding the extraction of a single, biologically interpretable signature. Our two-step procedure avoids high-dimensional instability, ensures biological comparability across species and conditions, and provides sufficient modeling freedom to reconstruct the ordinal axis without overfitting. **We have used and validated this approach in several studies** (Abe et al., 2024; Baumann et al., 2025; Klingler et al., 2021b; Telley et al., 2019).

We have now updated the methods for clarity on these points (p.20).

4. Were landscape comparisons corrected for differences in cell density across conditions? In all conditions there are sparsely populated areas in each landscape (Fig S3). In these regions, the k-NN process – averaging over the nearest 100 cells – may average expression over larger (and therefore more dissimilar) areas, spreading signal along each axis.

Yes, landscapes were corrected for differences in cell density for each condition so that positional distributions of cells along the age and differentiation ordinal axes are comparable across datasets with different density characteristics. To do this, the score distribution was adapted using a hyperbolic tangent transformation and adjusted scores were rescaled to the [0,1] range, standardizing scale and reducing outlier effects. We added detailed information about the approach in the **methods** (p.20, l.584-601) and provided access to the repositories: (https://github.com/gomez-L/humous) and (https://doi.org/10.17605/OSF.IO/E8GMA).

To test whether using lower or larger neighbor cells for averaging would impact landscape patterns, we performed a **new validation experiment** by generating all human landscapes for patterned genes, averaging 50 or 200 neighbor cells, and compared those to the 100 neighbors' original landscapes. The gene expression pixel to pixel mean correlations were highly correlated (50 *vs.* 100 neighbors mean correlation = 0.80, 200 *vs.* 100 neighbors mean correlation = 0.86). These results indicate that **changing the number of neighbors used for normalization has no substantial impact on the gene-expression patterns** obtained with a 100-neighbor setting. We added this analysis in the **new Extended Data Fig. 6** and **methods** (p.21, l.623-627).

5. VGG16 was used to generate image features – 25,0888 per landscape from 59,536 pixels (244x244). The top 2000 were then used for clustering. How does this compare to simply clustering the landscapes based on the top 2000 pixels (i.e.: without VGG embedding)?

Clustering based on the top 2000 raw pixels would rely only on absolute intensity at individual points, whereas VGG16 embeddings capture contextual structure (*e.g.* edges, textures, and spatial organization) by transforming each 244×244 landscape into 25,088 learned features. This allows the model to detect recurring structural motifs despite differences in brightness, scale, or noise, which pixel-based clustering cannot robustly resolve.

Consistent with this, prior work in biological imaging (cell morphology, histopathology, spatial transcriptomics) shows that pretrained convolutional network embeddings better preserve biologically meaningful groupings and spatial topology than raw-intensity clustering (*e.g.,* (Biancalani et al., 2021; Coudray et al., 2018). These points are now included in the **methods** (p.22, l.659-661).

6. In several of the low expression landscapes, circular artefacts are clearly present – potentially from the k-NN averaging? Have the authors considered applying smoothing, or outlier detection to mitigate these artefacts?

The circular artifacts indeed occur when very few cells express a given gene. To minimize such effects, we optimized the k-NN averaging parameters to balance smoothing while avoiding overestimation of expression from outlier cells, taking local cell density into account. In addition, we applied an outlier cutoff that excludes genes expressed in fewer than 1% of all cells. We also scaled gene expression values using the number of detected genes per cell as a regression factor, further reducing the influence of sparsely expressed genes. Robustness checks on the effects of k-NN averaging are now included in **Extended Data Fig. 6**, and these details have been clarified in the **methods** (p.20, l. 584-601 and p.21, l.623-627).

7. It would be valuable to perform the in-silico knockout with CellOracle using additional datasets. Workarounds allow its use without ATAC-seq data.

We thank the reviewer for this suggestion. We have now performed new CellOracle analysis including additional datasets (Bhaduri et al., 2021; Nowakowski et al., 2017; Trevino et al., 2021; van Bruggen et al., 2022; Wang et al., 2025), which now includes >100k cells across multiple ages (compared to 8k PCW21 cells in the previous analysis. This confirmed the initial results and identified additional upstream regulators and downstream pathways, which improved the *in silico* perturbation and GRN inferences analyses. These are now presented in **Figs. 4h & 5c**, and **Supplementary Tables 5 & 6**. This new analysis strengthened evidence for the shift towards differentiation upon JUNB KO (**Extended Data Fig. 15l**).

8. The integration of different datasets should be presented more clearly, by showing separate UMAPs colored by batch and relevant integration metrics.

The separate UMAPs colored by batch are presented in **Extended Data Fig. 1**.

To address integration metrics, we calculated the iLISI (integration Local Inverse Simpson's Index) score, which measures diversity of source datasets (batches) among the nearest neighbors in the integrated object (Korsunsky et al., 2019). When batches are well mixed (good integration), each neighborhood contains cells from many batches, which is what we observed across conditions. **This new data is presented in Extended Data Fig. 1a-c).**

9. The code provided on GitHub demonstrates only the application of CellOracle but does not include the full implementation of the workflow, which is important for reproducibility and reuse.

Access to codes has been provided (p.34).

All codes used for data integration, ordinal regression for age and differentiation axis reconstruction, landscapes generation and correlation, computer vision embeddings, and

landscape characterization were deposited on https://github.com/gomez-L/humous. Preprocessed datasets and intermediate data objects generated through this pipeline can be accessed at https://doi.org/10.17605/OSF.IO/E8GMA.

Codes used for the subsampling permutations, CellOracle, GRN, CUT&Tag / CUT&RUN, and lineage barcode analyses were deposited on https://github.com/awaisj14/Humous/.

10. Minor adjustments

L467: ensure numbers in each differentiation group match that shown in Table S1. Done (updated **Table S1**).

Figure 1a: Add a schematic for organoids too. We chose not to include a separate organoid schematic because our focus is on species comparison. Since organoid developmental timing varies by protocol and cell line the transcriptomic landscape comparison adequately contextualizes the data

Line 89: reference 52 (OrgH dataset – ID Ck19) is missing and its title in Suppl Table 1 is uncomplete. Thank you, this has been corrected.

L230: The suggestion that the JUNB promoter is poised for activation in mouse RG is a bit too speculative. Other explanations are possible.

Agreed. To document the poised state of the Junb promoter, we tested its activity in mouse RG by co-electroporating mouse and human *JUNB* promoters into E15.5 mouse cortex. Both were active in neurons but silent in RG (**new Fig. 5b**), despite being chromatin-accessible in human and mouse RG (**new Extended Data Fig. 17c**). This indicates that the promoter is poised but inactive in mouse RG, suggesting missing upstream regulators. To test this possibility, we co-electroporated **IRF1**, a human RG-specific *JUNB* regulator identified with CellOracle analysis. IRF1 activated both promoters in mouse RG, whereas mutating its binding sites reduced activation (**new Fig. 5e and Extended Data Fig. 18b**). Single-cell RNA-seq confirmed IRF1 induction of endogenous *Junb,* demonstrating functional relevance (**new Fig. 5f**).

Thus, the mouse *Junb* promoter contains accessible sites but lacks required human-specific factors — exemplified by IRF1 — explaining its poised state. **These new data appear in new Fig. 5 and Extended Data Figs. 17, 18** (p.9, l.294-298 and l.299-317).

L 83: Cortical "neurons" should be replaced with "cells"? Done.

L87: specify number of cells after balancing Done (p.3, l.105)

L103: should be low SD – high entropy? Yes, thank you, corrected.

L474: 750 total? (5 per age group, 3 diff groups) Yes, corrected.

L476: specify total number of genes Done (n = 2000 total genes, p.18).

L572: 'lower than -0.5' rather than 'smaller' for clarity Replaced by < (p.21).

Extended Figure 2a – legend, remove reference to Right: plot Done.

Extended Figure 9a– legend, 'fonction' Corrected.

Referee #3 (Remarks on code availability):

The code provided on GitHub demonstrates only the application of CellOracle but does not include the full implementation of the workflow. We now provide access to all the codes in *Code and data availability* (p.34).

Referee #4 (Remarks to the Author):

In the study titled "Developmental gene expression patterns driving species-specific cortical features", Javed et al. analyze developmental single-cell transcriptomes from mouse, human, and human organoids to identify conserved and divergent gene expression landscapes, highlighting the transcription factor JUNB. They combine CNN-based pattern recognition, correlation analysis, and functional experiments to propose that JUNB regulates progenitor proliferation and contributes to human-specific cortical features. The study introduces a novel trajectory-analysis method to extract new insights from existing data and validates JUNB as a candidate gene influencing brain expansion.

However, the claim that JUNB is human-specific is misleading since it is potentially conserved in macaque, suggesting a primate- or potentially mouse-specialization.

Similarly, disease enrichment claims are speculative and potentially misleading.

In sum, while the resource and functional data are valuable, the manuscript suffers from overinterpretation and lacks rigor in its evolutionary and disease relevance claims.

We thank the reviewer for their comments on our manuscript. We have now gone through the text to ensure that our terminology avoids any misinterpretation, and address the points raised through additional validation experiments and new data.

Major Comments:

1. The authors report greater expression patterning in mouse than human (Fig. 2a), but do not sufficiently control for quality differences in the source datasets. Human samples may suffer from greater tissue degradation or donor variability, confounding conclusions about species-specific gene patterning. A more rigorous cross-species normalization or benchmarking is needed to support these claims.

To address potential differences in dataset qualities, **we verified that our landscapes were not biased by number of counts or genes detected** (**Extended Data Fig. 2c**). This showed that our normalization approach allowed to balance for differences in absolute level of gene expression due to sequencing depth, for example.

For all conditions, published quality-controlled datasets were balanced during the cell selection, so that donor variability does not affect the landscape profiles. To address integration efficiency (*i.e.,* to assess whether one dataset might skew the overall data), **we calculated the iLISI score** (integration Local Inverse Simpson's Index score), which measures diversity of source datasets among nearest neighbors in the integrated UMAPs (Korsunsky et al., 2019). **This new analysis revealed good integration of the datasets in all conditions** (**Extended Data Fig. 1a-c**).

The point on donor variability is well taken: donor variability and tissue collection conditions are more difficult to control in human samples compared to mouse, which may introduce differences in gene expression. Since the datasets integrated here do not cluster by dataset - which each contain different donors - it is unlikely that bias linked to donor variability affects our results. We acknowledge, however, that sample quality can vary across human donors, and have noted this as a general consideration in interpreting differences in the number of patterned *vs*. non-patterned genes (p.4, l.129-130).

2. The observed divergence in IP gene expression (Fig. 2d–e) could be driven by sampling depth or expression noise. The authors should assess whether low gene detection in human IPs

or doublets contribute to these apparent differences. Prior literature should be cited to clarify if extended expression of cell cycle genes in human IPs is expected or novel.

The datasets used for the landscapes were all **published quality-controlled datasets**, where doublets from single cell RNA-seq data were filtered out (we verified this; Bhaduri et al., 2021; Di Bella et al., 2021; Kanton et al., 2019; Telley et al., 2019; Trevino et al., 2021; Velasco et al., 2019; Yuzwa et al., 2017) and our gene expression normalization approach further allows to balance for differences in absolute level of gene expression (see Point 1 above). The **new quality controls of the cells used for the landscapes** (presented in **Extended Data Fig. 2c**, see above) showed that IP do not present difference in counts or genes detected per cell, supporting interpretability.

We thank the reviewer for prompting us to dig deeper on the question of the biological relevance of extended expression of cell cycle genes in human IP. As it turns out, unlike in rodents, early born IP in human indeed retain RG-like proliferative and morphological features (potentially contributing to the emergence of OSVZ; Fietz et al., 2010). To better document this, **we added a comparison of gene expression specificity for all landscape clusters** (**Extended Data Fig. 10c**), showing that early Hu IP are the most divergent compared to Mo and Org_H, and now describe this finding in the results (p.5, l.161-166 and p.7, l.231-228).

3. The enrichment of disease-associated genes in IPs (Fig. 2f) is largely based on proximity to GWAS hits. As the authors note, many risk variants are regulatory and may not act during development. Without supporting chromatin accessibility (e.g., scATAC-seq) or enhancer maps, the assignment of developmental cell-type relevance should be de-emphasized.

Agreed. We toned down the assignment of developmental cell type relevance for disease (p.1, l.36 & p.10, l.342-345) and clarified how genes associated with diseases are defined in the **methods** (p.23, l.715).

4. JUNB is highlighted as a species-specific regulator, yet its expression in macaque organoids (ED Fig. 12b) contradicts the claim that it is human-specific. The analysis of enhancer accessibility focuses on the JUNB promoter (ED Fig. 12e), but regulation in RG is more likely mediated by distal enhancers. Why were putative enhancers not analyzed for TF enrichment with published human development ATAC-seq datasets, especially since CellOracle predicts regulators like FOS and EGR1?

Agreed. Regarding specificity, we have carefully checked the wording not to refer to human-specific but instead to **human-like**.

Following the reviewer's suggestion, we analyzed published developmental scATAC-seq datasets for TF binding motif enrichment to identify putative JUNB proximal (10kb) and distal (100kb) enhancers (datasets from Liu et al., 2025; Trevino et al., 2021; Wang et al., 2025). **In proximal enhancers, the majority of enriched motifs were species-specific while this was not the case in distal enhancers.** These results suggest that JUNB regulation through proximal enhancers is driven mainly by species-specific motifs, whereas distal control likely reflects species-specific availability of upstream regulators. **These new data are shown in Extended Data Fig. 17b and presented in the text** (p.9, l.293-298).

5. JUNB gain-of-function in mouse RG prolongs progenitor proliferation, consistent with previous reports that RG self-renewal regulates cortical expansion. However, this is not

sufficient to establish JUNB as a central driver of superficial layer neuron expansion since many other genes could be involved.

We apologize if our wording has given the impression that JUNB was considered as a central driver of superficial layer neuron expansion; we have gone through the text in detail to make sure that all parts were unambiguous with regard to this point (p.1, l.35-36 & p.10, l.336-337).

To better assess JUNB's position within a gene regulatory network ultimately allowing increased neuronal production, we took the following *in silico* and experimental approaches, which highlight a **broader active transcriptional program rather than singular expression of JUNB that confers Hu RG-like behavior**:

- ***In silico***: Expanded CellOracle GRN analysis taking advantage of the human datasets presented above (scRNA-seq data from Bhaduri et al., 2021; Nowakowski et al., 2017; Trevino et al., 2021; van Bruggen et al., 2022; Wang et al., 2025; Multiome data from Trevino et al., 2021; Wang et al., 2025) to identify putative upstream regulators and downstream effectors of JUNB function. Amongst upstream regulators, we identified a set of human RG-enriched transcription factors, including IRF1, which is not expressed in mouse.

- **Experimental**: We validated IRF1 as a direct regulator through CUT&RUN and functional promoter assays as described in response below.

6. The *in silico* perturbation using Cell Oracle (Fig. 4i) is suggestive but lacks validation. scATAC-seq or ChIP-seq data from human RG would be more appropriate to define JUNB targets and upstream regulators.

We thank the reviewer to prompt us in such a biological validation. To validate the *in silico* findings, focusing on the relationship between IRF1 and JUNB, **we performed the following experiments**:

- *Upstream regulators:* We performed CUT&RUN in DIV40 human-derived organoids to assess the binding profile of IRF1, a predicted upstream regulator of *JUNB*. We found IRF1 directly binds and activates *JUNB* promoter using 'GAAA' motifs, that are accessible in both Mo and Hu RG *JUNB* promoters. IRF1 overexpression increases *Junb* RG expression and induces an extension of the neurogenic windows in Mo, similar to JUNB. **These new results demonstrate that key human regulatory programs can be experimentally engaged in mouse tissue** (**Fig. 5**, **Extended Data Fig. 18** and **Supplementary Table 8**; p.8-9, l.282-317).

- *Downstream targets:* We performed CUT&Tag and found that JUNB directly binds to about half (48%) of the targets predicted by CellOracle GRN analysis (new **Fig. 4i**; **Extended Data Fig. 16**; **Supplementary Table 6**).We also overexpressed JUNB in mouse RG to assess changes in histone mark H3K27ac using CUT&Tag and found that JUNB not only binds to DNA but is able to remodel RG chromatin landscape through modulating histone acetylation in Mo (**Extended Data Fig. 16**; **Supplementary Table 6**; p.8, l.272-281).

Altogether, **these new data biologically validate predicted targets and upstream regulators of JUNB** (p.8-9).

7. The analysis of human vs. organoid expression patterns (Fig. 3a, ED Fig. 7f) is relatively underdeveloped. Many genes conserved in human and mouse are poorly modeled in organoids.

This is a key point for improving organoid utility as an evolutionary model and deserves deeper exploration.

Agreed. **We expanded the Org$_H$ analysis, and included a comparison with fetal human tissue**. The genes most strongly anti-correlated between human and Org$_H$ are cell-cycle–related (**Fig. 3e**), expressed in early IP in human but in RG in organoids (**Extended Data Fig. 7e,f**). In human cortex, early SVZ IP retain RG-like proliferative features that support OSVZ expansion (Fietz et al., 2010), which organoids do not recapitulate.

To probe this further, we examined TBR2$^+$ IP proliferation (KI67) in PCW10 human fetal tissue and 2-month organoids, in collaboration with the Nguyen laboratory. Most TBR2$^+$ cells were proliferative in human but not in organoids (**Extended Data Fig. 14a,b**), indicating that early IP are poorly modelled. Additional GRN analyses may help identify missing environmental cues, although this lies beyond the scope of the present study.

These new data are presented Extended Data Figures 7 and 14 and discussed in the text (p.7, l.233-238).

Minor Comments:

Fig. 1b – Neuronal organization by age is less clear in organoids. Authors should quantify this effect using trajectory distances or pseudotime dispersion.

To assess neuronal age alignment along trajectories in Org$_H$ and Hu, we computed rank-based correlations between predicted and real ages using Spearman's ρ and Kendall's τ. Both measures were significantly correlated with real ages ($p < 0.001$ in all conditions) but were lower in Org$_H$ ($\rho = 0.45$; $\tau = 0.35$) compared to Hu ($\rho = 0.67$; $\tau = 0.52$) and Mo ($\rho = 0.75$; $\tau = 0.60$), indicating less consistent age-related organization in organoids. **These new results are shown in Extended Data Figs. 4 and 5** for each cell type and age, respectively.

Fig. 2a – The GO analysis relies on a thresholded set of "patterned" genes. Could human genes involved in synapse organization have narrowly missed this cutoff?

To address this possibility, we looked at the fraction of synapse organization genes that were patterned in Mo and un-patterned in Hu. Fourteen genes (out of 53) patterned in mouse were non-patterned in human, showing broader or weaker specificity (**Figure for reviewer 4**). These differences may reflect species-specific regulation, such as increased pleiotropy in human (Klingler et al., 2021a; Nowakowski et al., 2025), or variability in donor tissue and collection quality (now mentioned in p.4, l.127–130).

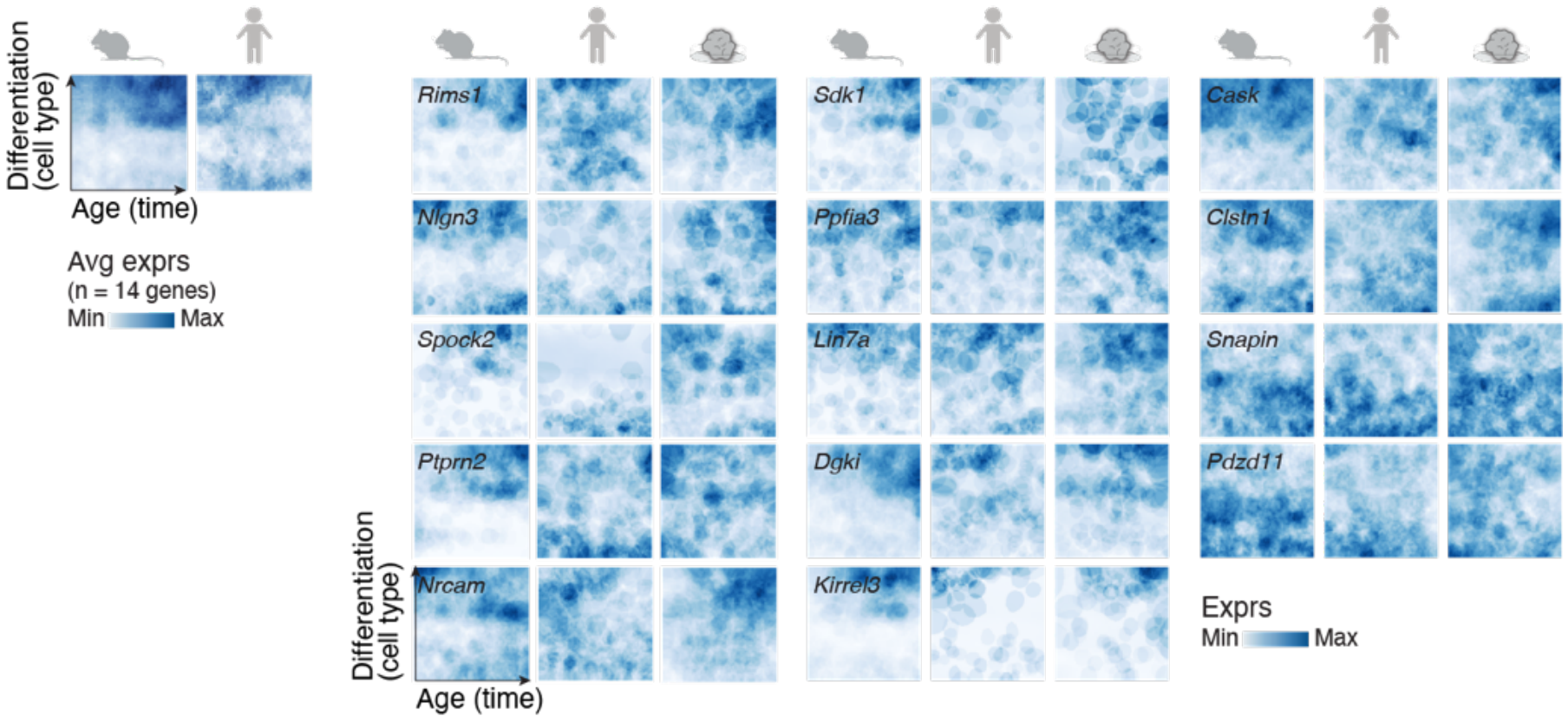

Figure for reviewer 4 | Expression of synapse genes patterned in mouse and un-patterned in human. Averaged expression (left) and gene expression landscapes (right) of synapse genes patterned in mouse and un-patterned in human.

Fig. 2e – Clarify what is compared in the correlation analyses. Do all IP clusters (c11, c12, c13) have similarly low cross-species correlations?

In **Fig. 2e**, we compared genes with similar or divergent expression across the five main categories (RG, RG-IP, IP, IP-N, N). **The legend was clarified, and an additional analysis of Vgg16 clusters (Extended Data Fig. 10c)** show that only the IP cluster 12 (early IP) displays low cross-species correlation. This likely reflects human-specific features of early SVZ IP, which retain RG-like proliferative traits enabling OSVZ expansion (Fietz et al., 2010). **This is now presented p.5, l.161-166.**

ED Fig. 10 – The timing differences observed are intriguing. However, the authors should discuss whether these are consistent with prior interspecies pseudotime studies.

Prior interspecies pseudotime alignment studies have consistently shown that human cortical neurogenesis is globally protracted, with delayed progression of both progenitor and neuronal maturation programs (Eze et al., 2021; Kanton et al., 2019; Nowakowski et al., 2017; Pollen et al., 2019), supporting the timing differences we observe here. **This is now highlighted in p.6, l.214**.

Referee #5 (Remarks to the Author): I co-reviewed this manuscript with one of the reviewers who provided the listed reports.

Referee #6 (Remarks to the Author): I co-reviewed this manuscript with one of the reviewers who provided the listed reports.

References

Abe, P., Lavalley, A., Morassut, I., Santinha, A.J., Roig-Puiggros, S., Javed, A., Klingler, E., Baumann, N., Prados, J., Platt, R.J., Jabaudon, D., 2024. Molecular programs guiding arealization of descending cortical pathways. Nature 634, 644–651. https://doi.org/10.1038/s41586-024-07895-y

Baumann, N., Wagener, R.J., Javed, A., Conti, E., Abe, P., Lopes, A., Sansevrino, R., Lavalley, A., Magrinelli, E., Szalai, T., Fuciec, D., Ferreira, C., Fièvre, S., Fouassier, A., D'Amico, D., Harschnitz, O., Jabaudon, D., 2025. Regional differences in progenitor metabolism shape brain growth during development. Cell 188, 3567-3582.e20. https://doi.org/10.1016/j.cell.2025.04.003

Bhaduri, A., Sandoval-Espinosa, C., Otero-Garcia, M., Oh, I., Yin, R., Eze, U.C., Nowakowski, T.J., Kriegstein, A.R., 2021. An atlas of cortical arealization identifies dynamic molecular signatures. Nature 598, 200–204. https://doi.org/10.1038/s41586-021-03910-8

Biancalani, T., Scalia, G., Buffoni, L., Avasthi, R., Lu, Z., Sanger, A., Tokcan, N., Vanderburg, C.R., Segerstolpe, Å., Zhang, M., Avraham-Davidi, I., Vickovic, S., Nitzan, M., Ma, S., Subramanian, A., Lipinski, M., Buenrostro, J., Brown, N.B., Fanelli, D., Zhuang, X., Macosko, E.Z., Regev, A., 2021. Deep learning and alignment of spatially resolved single-cell transcriptomes with Tangram. Nat. Methods 18, 1352–1362. https://doi.org/10.1038/s41592-021-01264-7

Coudray, N., Ocampo, P.S., Sakellaropoulos, T., Narula, N., Snuderl, M., Fenyö, D., Moreira, A.L., Razavian, N., Tsirigos, A., 2018. Classification and mutation prediction from non-small cell lung cancer histopathology images using deep learning. Nat. Med. 24, 1559–1567. https://doi.org/10.1038/s41591-018-0177-5

Di Bella, D.J., Habibi, E., Stickels, R.R., Scalia, G., Brown, J., Yadollahpour, P., Yang, S.M., Abbate, C., Biancalani, T., Macosko, E.Z., Chen, F., Regev, A., Arlotta, P., 2021. Molecular logic of cellular diversification in the mouse cerebral cortex. Nature 595, 554–559. https://doi.org/10.1038/s41586-021-03670-5

Eze, U.C., Bhaduri, A., Haeussler, M., Nowakowski, T.J., Kriegstein, A.R., 2021. Single-cell atlas of early human brain development highlights heterogeneity of human neuroepithelial cells and early radial glia. Nat. Neurosci. 24, 584–594. https://doi.org/10.1038/s41593-020-00794-1

Fietz, S.A., Kelava, I., Vogt, J., Wilsch-Bräuninger, M., Stenzel, D., Fish, J.L., Corbeil, D., Riehn, A., Distler, W., Nitsch, R., Huttner, W.B., 2010. OSVZ progenitors of human and ferret neocortex are epithelial-like and expand by integrin signaling. Nat. Neurosci. 13, 690–699. https://doi.org/10.1038/nn.2553

Giorgino, T., 2009. Computing and Visualizing Dynamic Time Warping Alignments in *R* : The **dtw** Package. J. Stat. Softw. 31. https://doi.org/10.18637/jss.v031.i07

Kalousis, A., Prados, J. & Hilario, M. Stability of feature selection algorithms: a study on high-dimensional spaces. *Knowl. Inf. Syst.* **12**, 95–116 (2007).

Kanton, S., Boyle, M.J., He, Z., Santel, M., Weigert, A., Sanchís-Calleja, F., Guijarro, P., Sidow, L., Fleck, J.S., Han, D., Qian, Z., Heide, M., Huttner, W.B., Khaitovich, P., Pääbo, S., Treutlein, B., Camp, J.G., 2019. Organoid single-cell genomic atlas uncovers human-specific features of brain development. Nature 574, 418–422. https://doi.org/10.1038/s41586-019-1654-9

Keefe, M.G., Steyert, M.R., Nowakowski, T.J., 2025. Lineage-resolved atlas of the developing human cortex. Nature 647, 194–202. https://doi.org/10.1038/s41586-025-09033-8

Klingler, E., Francis, F., Jabaudon, D., Cappello, S., 2021a. Mapping the molecular and cellular complexity of cortical malformations. Science 371, eaba4517. https://doi.org/10.1126/science.aba4517

Klingler, E., Tomasello, U., Prados, J., Kebschull, J.M., Contestabile, A., Galiñanes, G.L., Fièvre, S., Santinha, A., Platt, R., Huber, D., Dayer, A., Bellone, C., Jabaudon, D., 2021b. Temporal controls over inter-areal cortical projection neuron fate diversity. Nature 599, 453–457. https://doi.org/10.1038/s41586-021-04048-3

Korsunsky, I., Millard, N., Fan, J., Slowikowski, K., Zhang, F., Wei, K., Baglaenko, Y., Brenner, M., Loh, P.-R., Raychaudhuri, S., 2019. Fast, sensitive and accurate integration of single-cell data with Harmony. Nat. Methods 16, 1289–1296. https://doi.org/10.1038/s41592-019-0619-0

Liu, Y., Luo, X., Sun, Y., Chen, K., Hu, T., You, B., Xu, J., Zhang, F., Cheng, Q., Meng, X., Yan, T., Li, X., Qi, X., He, X., Guo, X., Li, C., Su, B., 2025. Comparative single-cell multiome identifies evolutionary changes in neural progenitor cells during primate brain development. Dev. Cell 60, 414-428.e8. https://doi.org/10.1016/j.devcel.2024.10.005

Nowakowski, T.J., Bhaduri, A., Pollen, A.A., Alvarado, B., Mostajo-Radji, M.A., Di Lullo, E., Haeussler, M., Sandoval-Espinosa, C., Liu, S.J., Velmeshev, D., Ounadjela, J.R., Shuga, J., Wang, X., Lim, D.A., West, J.A., Leyrat, A.A., Kent, W.J., Kriegstein, A.R., 2017. Spatiotemporal gene expression trajectories reveal developmental hierarchies of the human cortex. Science 358, 1318–1323. https://doi.org/10.1126/science.aap8809

Nowakowski, T.J., Nano, P.R., Matho, K.S., Chen, X., Corrigan, E.K., Ding, W., Gao, Y., Heffel, M., Jayakumar, J., Kaplan, H.S., Kronman, F.N., Kovner, R., Mannens, C.C.A., Song, M., Steyert, M.R., Venkatesan, S., Wallace, J.L., Wang, L., Werner, J.M., Zhang, D., Yuan, G., Zuo, G., Ament, S.A., Colantuoni, C., Dulac, C., Fan, R., Gillis, J., Kriegstein, A.R., Krienen, F.M., Kim, Y., Linnarsson, S., Mitra, P.P., Pollen, A.A., Sestan, N., Tward, D.J., van Velthoven, C.T.J., Yao, Z., Bhaduri, A., Zeng, H., 2025. The new frontier in understanding human and mammalian brain development. Nature 647, 51–59. https://doi.org/10.1038/s41586-025-09652-1

Nowakowski, T.J., Pollen, A.A., Sandoval-Espinosa, C., Kriegstein, A.R., 2016. Transformation of the radial glia scaffold demarcates two stages of human cerebral cortex development. Neuron 91, 1219–1227. https://doi.org/10.1016/j.neuron.2016.09.005

Pollen, A.A., Bhaduri, A., Andrews, M.G., Nowakowski, T.J., Meyerson, O.S., Mostajo-Radji, M.A., Di Lullo, E., Alvarado, B., Bedolli, M., Dougherty, M.L., Fiddes, I.T., Kronenberg, Z.N., Shuga, J., Leyrat, A.A., West, J.A., Bershteyn, M., Lowe, C.B., Pavlovic, B.J., Salama, S.R., Haussler, D., Eichler, E.E., Kriegstein, A.R., 2019. Establishing cerebral organoids as models of human-specific brain evolution. Cell 176, 743-756.e17. https://doi.org/10.1016/j.cell.2019.01.017

Pollen, A.A., Nowakowski, T.J., Chen, J., Retallack, H., Sandoval-Espinosa, C., Nicholas, C.R., Shuga, J., Liu, S.J., Oldham, M.C., Diaz, A., Lim, D.A., Leyrat, A.A., West, J.A., Kriegstein, A.R., 2015. Molecular identity of human outer radial glia during cortical development. Cell 163, 55–67. https://doi.org/10.1016/j.cell.2015.09.004

Telley, L., Agirman, G., Prados, J., Amberg, N., Fièvre, S., Oberst, P., Bartolini, G., Vitali, I., Cadilhac, C., Hippenmeyer, S., Nguyen, L., Dayer, A., Jabaudon, D., 2019. Temporal patterning of apical progenitors and their daughter neurons in the developing neocortex. Science 364, eaav2522. https://doi.org/10.1126/science.aav2522

Trevino, A.E., Müller, F., Andersen, J., Sundaram, L., Kathiria, A., Shcherbina, A., Farh, K., Chang, H.Y., Pașca, A.M., Kundaje, A., Pașca, S.P., Greenleaf, W.J., 2021. Chromatin and gene-regulatory dynamics of the developing human cerebral cortex at single-cell resolution. Cell 184, 5053-5069.e23. https://doi.org/10.1016/j.cell.2021.07.039

Uzquiano, A., Kedaigle, A.J., Pigoni, M., Paulsen, B., Adiconis, X., Kim, K., Faits, T., Nagaraja, S., Antón-Bolaños, N., Gerhardinger, C., Tucewicz, A., Murray, E., Jin, X., Buenrostro, J., Chen, F., Velasco, S., Regev, A., Levin, J.Z., Arlotta, P., 2022. Proper acquisition of cell class identity in organoids allows definition of fate specification programs of the human cerebral cortex. Cell 185, 3770-3788.e27. https://doi.org/10.1016/j.cell.2022.09.010

van Bruggen, D., Pohl, F., Langseth, C.M., Kukanja, P., Lee, H., Albiach, A.M., Kabbe, M., Meijer, M., Linnarsson, S., Hilscher, M.M., Nilsson, M., Sundström, E., Castelo-Branco, G., 2022. Developmental landscape of human forebrain at a single-cell level identifies early waves of oligodendrogenesis. Dev. Cell 57, 1421-1436.e5. https://doi.org/10.1016/j.devcel.2022.04.016

Velasco, S., Kedaigle, A.J., Simmons, S.K., Nash, A., Rocha, M., Quadrato, G., Paulsen, B., Nguyen, L., Adiconis, X., Regev, A., Levin, J.Z., Arlotta, P., 2019. Individual brain organoids reproducibly form cell diversity of the human cerebral cortex. Nature 570, 523–527. https://doi.org/10.1038/s41586-019-1289-x

Wang, L., Wang, C., Moriano, J.A., Chen, S., Zuo, G., Cebrián-Silla, A., Zhang, S., Mukhtar, T., Wang, S., Song, M., de Oliveira, L.G., Bi, Q., Augustin, J.J., Ge, X., Paredes, M.F., Huang, E.J., Alvarez-Buylla, A., Duan, X., Li, J., Kriegstein, A.R., 2025. Molecular and cellular dynamics of the developing human neocortex. Nature. https://doi.org/10.1038/s41586-024-08351-7

Yuzwa, S.A., Borrett, M.J., Innes, B.T., Voronova, A., Ketela, T., Kaplan, D.R., Bader, G.D., Miller, F.D., 2017. Developmental emergence of adult neural stem cells as revealed by single-cell transcriptional profiling. Cell Rep. 21, 3970–3986. https://doi.org/10.1016/j.celrep.2017.12.017

Zhou, J., Vitali, I., Roig-Puiggros, S., Javed, A., Cantando, I., Puglisi, M., Bezzi, P., Jabaudon, D., Mayer, C., Bocchi, R., 2025. Dual lineage origins contribute to neocortical astrocyte diversity. Nat. Commun. 16, 6992. https://doi.org/10.1038/s41467-025-61829-4

Referees' comments:

Referee #1 (Remarks to the Author):

The authors have addressed concerns regarding alignment of developmental age across species, as well as many other points raised in the first round of review. However, in Extended Data Fig. 5d, the dynamic time warping (DTW) analysis appears to have been performed using the original dataset starting from PCW12 onward, rather than the full extended dataset. It would be helpful to include data from PCW8–12 in humans and E10–11 in mice, and to assess whether DTW still aligns E12 in mouse with PCW12 in human when these earlier developmental stages are incorporated.

We thank the reviewer for pointing this out. **We have now performed the DTW analysis including the additional time points (new Extended Data Fig. 5d)**. This analysis confirms the global pattern of human-mouse time-point matching.

A related concern pertains to the alignment of the differentiation axis, which relies on accurate annotation of radial glia (RG), intermediate progenitors (IP), and neurons. While marker-based annotation is discussed, the differentiation axis itself critically depends on the fidelity of these cell-type assignments. In Extended Data Fig. 1a and 1b, although nearly all mouse IPs are EOMES-positive, a substantial fraction of human cells annotated as IP appear to be EOMES-negative, as also reflected in the violin plots in Extended Data Fig. 7a,c,d (specifically the IP1 cluster). This raises the possibility that a subset of human RGs may be misannotated as IPs.
Such potential misassignment could influence downstream analyses, including the reported differences in IPC gene expression patterns between human and mouse. To address this concern, it may be helpful for the authors to consider label transfer or data integration–based approaches to define RG, IP, and neuron populations across species or platforms. Ideally, these approaches would yield results consistent with the current annotations; if not, some conclusions may need to be revisited.

Regarding the potential misannotation of low-EOMES IPs (IP1 cluster, **Extended Data Fig. 7a-d**), several observations support a genuine IP identity. First, RG-like IPs with low *EOMES* have been described in the human SVZ; they co-express *PPP1R17* and *INSM1* together with *VIM* and *SOX2*, and display a characteristic IP morphology, (Pebworth et al., 2021). **This molecular profile corresponds to that of the cells found in the IP1 cluster (new Extended Data Fig. 7a)**. Second, in a new analysis using nearest-neighbor analysis along the RG→IP→N trajectory (Methods, p.21, l.583-593), we find that IP1 cells preferentially share neighbors with *EOMES*- or *PPP1R17*-expressing cells (putative IP) rather than with aRG or oRG populations (**new Extended Data Fig. 7d**). Hence, IP1 cells are most likely transitioning IPs with residual RG-like features, rather than misannotated RGs. **These new data have been included in the revised manuscript** (p.7, l.241-242).

Regarding the point on data integration, we apologize for the lack of clarity. Data integration was in fact the approach we used to merge platforms. We chose this strategy over label transfer because it does not assume that any single dataset provides a ground-truth reference annotation. **The integration workflow and associated quality control steps are now clarified and described in greater detail in the Methods** (p.20, l.557-561).

Referee #3 (Remarks to the Author):

I appreciate the authors' efforts in considering my comments.

A major concern, also raised by other reviewers, was related to the age alignment across species (Comment #1). The additional information provided by the authors improves this aspect, both in the discussion of how they chose the temporal landmarks and the additional validation and extra data. However, this is a critical aspect upon which major analyses are based and should be addressed in detail and supported by analysis in the main text (now it is discussed in Extended Data Figures and Methods).

While the distinction between L6 and L2/3 neurogenesis mitigate some of the concerns, the results do not really provide a "comprehensive insight into cortical cell-type specific and temporal patterns of gene expression across species and context", as claimed by the authors in the discussion.

We have now inserted the description of temporal landmark selection and the validation of cross-species matches into the **main text** as requested (p.3, l.94-98; p.4, l.117). In addition, we have modified the corresponding statement in the **Discussion** (p.10, l.329-330).

The distinction between basal radial glia (bRG) and truncated radial glia (tRG)(Comment #3) deserves more consideration. These RG subtypes are clearly transcriptionally distinct as shown in Ext Fig 7a-c. A more thorough analysis would strengthen the manuscript.

We thank the reviewer for prompting a deeper analysis of this point. tRG express a limited set of markers (e.g., *CRYAB*, *SPARCL1*, *ANXA1*) but remain transcriptionally close to apical RG, whereas oRG/bRG are characterized by canonical markers such as *HOPX*, *PTPRZ1*, and *TNC* (Pollen et al., 2015). Taking advantage of these markers, we thus expanded Extended Data Fig. 7a–c to include a broader panel of established RG-subtype markers and delineate these cell populations. **This new analysis shows that tRG are enriched in the RG1 cluster (new Extended Data Fig. 7a–d). Consistent with their reported emergence during mid-corticogenesis, both tRG and oRG increase with age (new Extended Data Fig. 7d)**. The manuscript text has been updated accordingly (p.3, l.106-110).

The organoid work is not fully convincing. The two images in Extended Data Figure 14 show considerable variability in organoid morphology, suggesting that a larger sample size is needed to confirm the findings.

Organoid macromorphology is inherently variable, primarily reflecting differences in ventricular number and spatial organization. Comparable variability is observed in control organoids, indicating that these large-scale features cannot be attributed to electroporation. Moreover, such structural characteristics are unlikely to be altered by electroporation within the short 7-day time window examined. To further document this variability beyond standard ventricle-based quantifications (e.g. Kielkowski et al., Nat Commun 2020; Denoth-Lippuner et al., Cell Stem Cell 2021; Lancaster et al., Nature 2013; Bian et al., Nat Methods 2018), we additionally annotated ventricle-level measurements of N/RG ratio and VZ thickness with organoid identity and calculated organoid-level averages (**new Fig. 4c; Extended Data Fig. 14b,c**; Methods pp.27 and 31, l.828-831 and 957-962). **These organoid-level analyses confirm the increased N/RG ratio upon JUNB KO.**

Furthermore, several inconsistencies in the organoid work are evident. In the Methods section, the authors state that "In organoids, we focused on stages in vitro where RG, IP, and N were found in substantial numbers in the original datasets, following Uzquiano et al Cell 2022 (ref 82)". However, this reference dataset was generated using a guided organoid model, which differs from the unguided model used by the authors (Giandomenico 2021, Lancaster 2014). Therefore, it cannot serve as a valid reference. This discrepancy reinforces concerns about inappropriate correspondence between timepoints across datasets (Comment#1), extending it to the in vivo and in vitro analysis.

Additionally, organoids used for the Cut&Tag and Cut&Run experiments were produced using the Stem Cell Technology kit, which, although based on the Giandomenico and Lancaster protocols, represents a distinct method.

The reviewer refers to an inconsistency in the reference cited in the Methods section. To address this, we replaced the Uzquiano et al. reference with a more comprehensive and recent reference, which integrates 36 single-cell transcriptomic datasets spanning 26 guided and unguided organoid protocols and maps organoid cell states to the developing human brain (He et al., Nature 2024).Using this resource, we additionally examined RG, IP, and neuron distributions across in vitro ages and compared it with our data. This confirms that neuronal lineages are rare before ~1 month in vitro across protocols (<20%), with progenitors dominating earlier stages. **This has now been added to the text** (p.3, l.102).

We apologize for the lack of clarity regarding the Cut&Tag and Cut&Run organoid protocols. These experiments were not designed to establish correspondence with the time-point datasets. Rather, their purpose was to validate JUNB regulatory interactions in human neural progenitors. Using a standardized kit for unguided cerebral organoid differentiation therefore strengthens the generalizability of our findings, rather than limiting them to a single organoid protocol. This has now been clarified in the Methods (p.27, l.834-835).

Referee #4 (Remarks to the Author):

The authors have addressed my comments, particularly making it clear that the observed features are "human-like" not necessarily "human-specific". I support publication.

Referee #5 (Remarks to the Author):

I co-reviewed this manuscript with one of the reviewers who provided the listed reports.

Referee #5 (Remarks on code availability):

They appear to have archived the code and data objects, the analysis scripts look fine. It would take a lot of work to apply their method to a new dataset, but they're not really trying to publish a tool.
