# Supplementary material for: Developmental gene expression patterns driving species-specific cortical features
Source: Nature. 2026 May 13;655(8123):694–702. doi: 10.1038/s41586-026-10491-x (PMC13661261; doi:10.1038/s41586-026-10491-x)
Supplement: Supplementary file 2 [file 41586_2026_10491_MOESM1_ESM.pdf]

nature portfolio

https://doi.org/10.1038/s41586-026-10491-x

Supplementary information

Developmental gene expression patterns driving species-specific cortical features

In the format provided by the authors and unedited

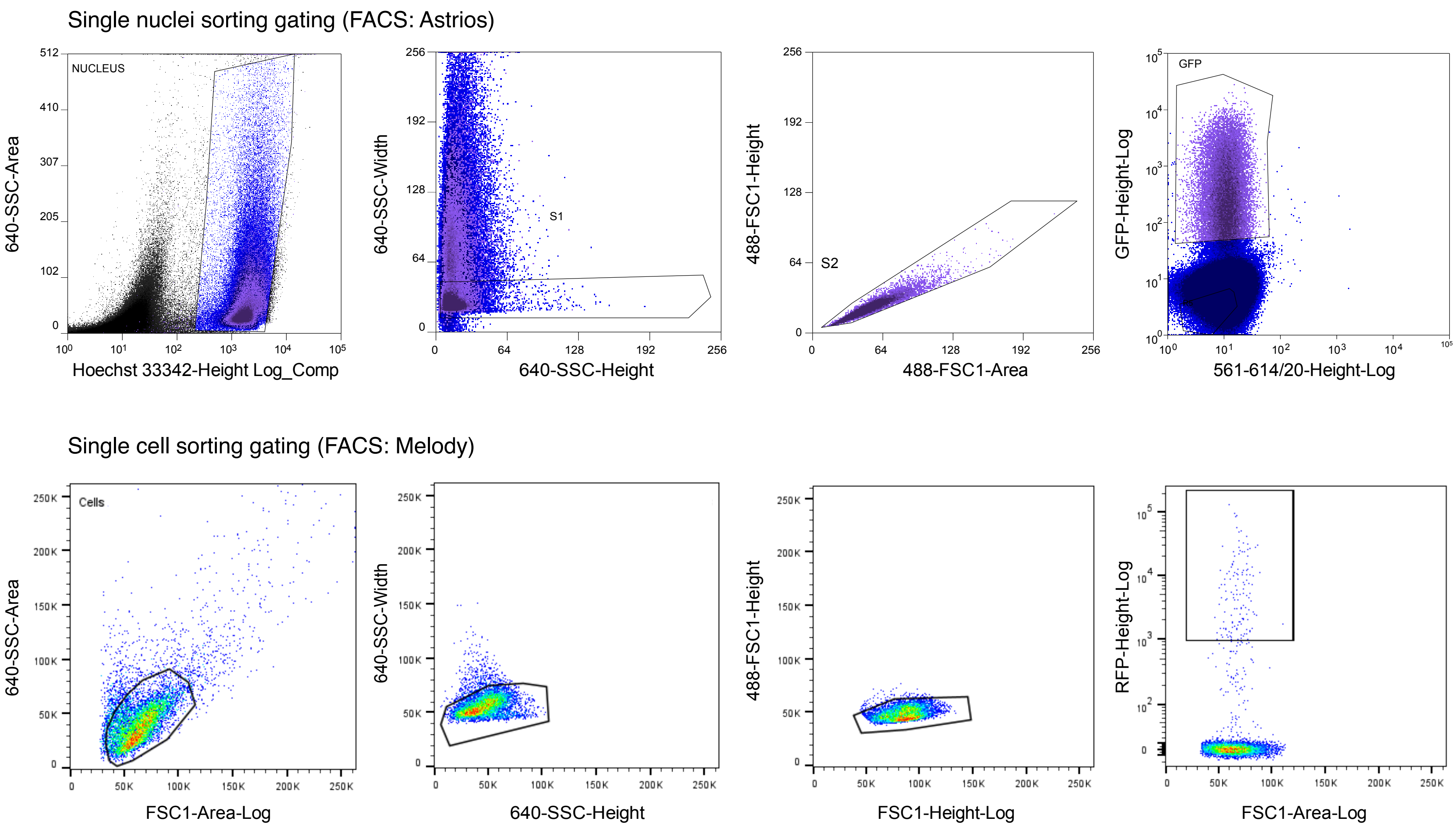

Supplementary Information Fig. 1 | Illustration of gating strategies used for fluorescence-activated single nucleus/cell sorting.
