# Supplementary material for: Developmental gene expression patterns driving species-specific cortical features
Source: Nature. 2026 May 13;655(8123):694–702. doi: 10.1038/s41586-026-10491-x (PMC13661261; doi:10.1038/s41586-026-10491-x)
Supplement: Supplementary file 3 [file 41586_2026_10491_MOESM2_ESM.pdf]

nature portfolio

Corresponding author(s): Esther Klingler, Denis Jabaudon

Last updated by author(s): 11-03-26

Reporting Summary

Nature Portfolio wishes to improve the reproducibility of the work that we publish. This form provides structure for consistency and transparency in reporting. For further information on Nature Portfolio policies, see our Editorial Policies and the Editorial Policy Checklist.

Statistics

For all statistical analyses, confirm that the following items are present in the figure legend, table legend, main text, or Methods section.

n/a	Confirmed	
☐	☒	The exact sample size (*n*) for each experimental group/condition, given as a discrete number and unit of measurement
☐	☒	A statement on whether measurements were taken from distinct samples or whether the same sample was measured repeatedly
☐	☒	The statistical test(s) used AND whether they are one- or two-sided<br>*Only common tests should be described solely by name; describe more complex techniques in the Methods section.*
☐	☒	A description of all covariates tested
☐	☒	A description of any assumptions or corrections, such as tests of normality and adjustment for multiple comparisons
☐	☒	A full description of the statistical parameters including central tendency (e.g. means) or other basic estimates (e.g. regression coefficient) AND variation (e.g. standard deviation) or associated estimates of uncertainty (e.g. confidence intervals)
☐	☒	For null hypothesis testing, the test statistic (e.g. *F*, *t*, *r*) with confidence intervals, effect sizes, degrees of freedom and *P* value noted<br>*Give P values as exact values whenever suitable.*
☒	☐	For Bayesian analysis, information on the choice of priors and Markov chain Monte Carlo settings
☒	☐	For hierarchical and complex designs, identification of the appropriate level for tests and full reporting of outcomes
☐	☒	Estimates of effect sizes (e.g. Cohen's *d*, Pearson's *r*), indicating how they were calculated

Our web collection on statistics for biologists contains articles on many of the points above.

Software and code

Policy information about availability of computer code

Data collection

Images were captured using Zeiss LSM800, LSM880, Nikon Ti-2 Eclipse and Leica MICA confocal microscopes. Cells were sorted using either Beckman Coulter MoFlo Astrios with SUMMIT (6.3.1) or BD FACSMelody with BD FACSChorus (6.1). Single cell/nuclei RNA sequencing data was generated using 10x Genomics 3' Gene Expression v3.1 kit and NovaSeq 5000/6000. Please refer to the methods section of the manuscript for precise description of the data collection equipment.

Data analysis

Illumina's bcl2fastq (version 2.20.0.422) was used for primary processing of raw sn/scRNAseq sequencing data to Fastq files.
UNIX packages: Cellranger (7.2.0), bbmap (35.85), samtools (1.15), umi-tools (1.1.4), cellbender (0.3.0).
R (4.3.2) packages: Clusterprofiler (3.2.1), Monocle3 (1.4.20), SeuratWrappers (0.3.5), bmrm (4.1), Seurat for Humous dataset curation (V3), Seurat for single cell analysis (5.1.0), ggplot2 (3.5.1), UpSetR (1.4.0), dtw (1.23), cicero (1.20.0), DescTools (0.99.60), diptest (0.77-1), stats (4.3.2), miniconda Python for VGG16 (3.7), entropy (1.3.0), LISI (1.0), tanh base R (4.3.2), raster (3.5.2), ontologyIndex (2.7), SingleCellSignalR (v2).

Python for Celloracle (3.11.0) packages: scanpy (1.9.3), numpy (1.26.2), pandas (2.1.4), CellOracle (0.14.0), matplotlib (3.7.0), gimmemotifs (0.17.0), trimmomatic (0.39), bowtie2 (2.5.4), Sambamba (1.0.1), Picard (3.4.0), bedtools (2.31.1), Samtools (1.22.1), Deeptools (3.5.6), SEACR (1.3), IGV browser (2.18.2), GREAT (4.0.4).

Softwares: GraphPad Prism (10.0.1), Microsoft Excel (16.107), Fiji ImageJ (1.54f).

All landscapes of gene expression and correlation values are available on the interactive viewer https://www.humous.org/.
All codes used for data integration, ordinal regression for age and differentiation axis reconstruction, landscapes generation and correlation, computer vision embeddings, and landscape characterization were deposited on https://github.com/gomez-L/humous. Preprocessed datasets

and intermediate data objects generated through this pipeline can be accessed at https://doi.org/10.17605/OSF.IO/E8GMA.
Codes used for the subsampling permutations, CellOracle, GRN, CUT&Tag / CUT&RUN, and lineage barcode analyses were deposited on https://github.com/awaisj14/Humous/.

For manuscripts utilizing custom algorithms or software that are central to the research but not yet described in published literature, software must be made available to editors and reviewers. We strongly encourage code deposition in a community repository (e.g. GitHub). See the Nature Portfolio guidelines for submitting code & software for further information.

Data

Policy information about availability of data

All manuscripts must include a data availability statement. This statement should provide the following information, where applicable:

- Accession codes, unique identifiers, or web links for publicly available datasets
- A description of any restrictions on data availability
- For clinical datasets or third party data, please ensure that the statement adheres to our policy

Previously published datasets used in this study: Mouse GEO GSE118953, GSE107122, GSE153164, GSE241429, SRA PRJNA637987; Human dbGaP: phs000989.v3, NeMo nemo:dat-9jd8xw6, GEO GSE162170, NeMo nemo:dat-oiif74w, EGA S00001006136. All raw and processed single cell transcriptomics data generated in this study were submitted to GEO under GSE324846.
Genomes: sn/scRNA analysis: refdata-gex-mm10-2020-A and custom cellranger reference built with refdata-gex-mm10-2020-A with hEGR1 (CCDS4206.1, ensembl), hIRF1 (CCDS4155.1, ensembl) and hJUNB (CCDS12280.1, ensembl), TurboRFP-NLS (https://github.com/awaisj14/Humous), PBase (https://github.com/awaisj14/Humous) sequences.
CUT&Tag/RUN analysis: human (GRCh38-noalt), yeast (R64-1-1), mouse (mm10) or E.coli genome (str. 536).

Research involving human participants, their data, or biological material

Policy information about studies with human participants or human data. See also policy information about sex, gender (identity/presentation), and sexual orientation and race, ethnicity and racism.

Reporting on sex and gender	Sex was not included as a biological variable because the fetal samples were obtained at early developmental ages when sex-related transcriptional and physiological differences are minimal. No data was collected about the identity or demographics of any patients that tissue is obtained from.
Reporting on race, ethnicity, or other socially relevant groupings	No data was collected about the identity or demographics of any patients that tissue is obtained from.
Population characteristics	Please see above.
Recruitment	Human fetal brain tissue was collected following voluntary pregnancy terminations performed at CHR Hospital–Site Laveu (Liège, Belgium). All procedures were conducted in accordance with the Declaration of Helsinki, Good Clinical Practice, and GDPR regulations, with written informed consent obtained from all mother donors. No data was collected about the identity or demographics of any patients that tissue is obtained from thereby limiting self-selection bias.
Ethics oversight	Ethical approval was secured under protocol number 2021/216 by the University Hospital-Faculty Ethics Committee of Liège.

Note that full information on the approval of the study protocol must also be provided in the manuscript.

Field-specific reporting

Please select the one below that is the best fit for your research. If you are not sure, read the appropriate sections before making your selection.

☒ Life sciences ☐ Behavioural & social sciences ☐ Ecological, evolutionary & environmental sciences

For a reference copy of the document with all sections, see nature.com/documents/nr-reporting-summary-flat.pdf

Life sciences study design

All studies must disclose on these points even when the disclosure is negative.

Sample size	No statistical analysis was performed to pre-determine sample size, a sample size of a minimum 3 biological replicates was considered sufficient based on what is being used in the field (Tsui et al. 2013, Chui et al. 2020, Stepien et al. 2020, Mangoni et al. 2023), except for CUT&Tag/CUT&RUN data in which included 2 pooled biological replicates from the same batch (Zenk et al. 2024).
Data exclusions	For single nucleus/cell experiments, the quality control metrics for nuclei and cells are listed in the methods section. Briefly, scDblFinder and Cellbender identified doublets/poor quality cells with ambient RNA contamination were removed. Poorly electroporated cortices (with ~<1mm area) were excluded from collections due to poor reporter positive enrichment with FACS/FANS and poor transgene expression signal.
Replication	Data with electroporations presented in Fig4d/5h were performed on one litter with multiple biological replicates per experimental condition (3-4 individual embryos within a litter) whereas all other data with electroporations were repeated at least once on a different litter and were

always successful. Multiple electroporated cortices from different animals were pooled for the single cell/nuclei experiments, detailed in the methods.

Randomization	Embryonic Experiments: For all in vivo experiments, embryos within the same litter were randomly allocated to receive either control or experimental plasmids. Sex was not used as a covariate for allocation as tissue was collected at developmental stages prior to sexual differentiation.<br><br>Organoid Experiments: For CUT&Tag/RUN analysis, experimental groups were established by selecting healthy, morphologically similar organoids from the same culture batch. This approach was used to minimize technical variability and ensure baseline biological consistency between the control and experimental samples.
Blinding	Data Collection: Investigators were blinded to group allocation during all experiments.<br>Data Analysis: Analysis was performed blindly for all electroporation experiments, except for promoter assays where a binary (on/off) signal made blinding unfeasible. Blinding was not performed for single-cell/nuclei RNA-sequencing because knowledge of sample identity is required for computational quality control, batch correction, and cell-type cluster annotation.

Reporting for specific materials, systems and methods

We require information from authors about some types of materials, experimental systems and methods used in many studies. Here, indicate whether each material, system or method listed is relevant to your study. If you are not sure if a list item applies to your research, read the appropriate section before selecting a response.

Materials & experimental systems

n/a	Involved in the study
☐	☒ Antibodies
☐	☒ Eukaryotic cell lines
☒	☐ Palaeontology and archaeology
☐	☒ Animals and other organisms
☒	☐ Clinical data
☒	☐ Dual use research of concern
☒	☐ Plants

Methods

n/a	Involved in the study
☐	☒ ChIP-seq
☐	☒ Flow cytometry
☒	☐ MRI-based neuroimaging

Antibodies

Antibodies used	Monoclonal mouse anti-SOX2 (1:500; AMAb91307; lot #MAB-04006; cloneID CL4716; Atlas)<br>Chicken anti-GFP (1:1000; GFP-1020; lot #GFP917979; Aves Labs)<br>Monoclonal rabbit anti-NEUROD2 (1:300; ab109406; lot #GR3249611-4; cloneID EPR5135; Abcam)<br>Rabbit anti-JUNB (1:100; 10486-1-AP; lot #00039797, Proteintech)<br>Rabbit anti-SOX2 (1:200; ab97959; lot #1033979-3; Abcam)<br>mouse anti-EOMES (1:100; WD1928; lot #3009178; ThermoFisher Scientific) <------<br>Rat anti-EOMES (1:200; Dan11mag; lot #4348755; Invitrogen)<br>Rabbit anti-NEUROD2 (1:400; ab104430; lot #GR118831-4; Abcam)<br>Chicken anti-GFP (1:1000; ab13970; lot #1018752-11; Abcam)<br>Monoclonal rat anti-BrdU (1:500; ab6326; lot #GR3365969; cloneID BU1/75 (ICR1); Abcam)<br>Monoclonal mouse anti-PAX6 (1:200, 13B10-1A10; lot #ZK401725; ThermoFisher Scientific)<br>Rat anti-RFP (1:500; 5F8; lot #90228002AB-06; cloneID 5F8; Proteintech)<br>Sheep anti-TBR2 (1:150; AF6166; lot #CDIP0319091; R&D Systems)<br>Rabbit anti-KI67 (1:100; AB15580; lot #GR250214; Abcam)<br>Rabbit anti-H3K27ac (1:50; ab177178; lot #GR3203987-19; Abcam)<br>Rabbit anti-IRF1 (1:50; 11335-1-AP; lot #00173582; Proteintech)<br>Rabbit anti-H3K4me3 (1:50; #9751, lot #16; CST)<br>Normal Rabbit IgG (1:50; #2729; lot #11; CST)<br><br>ChromoTek (alpaca anti-Mouse IgG1 nanobody: A488: sms1AF488-1, AB_2827578; A568: sms1AF568-1, AB_2827578; A647: smsG1CL647-1, AB_2941312) and ThermoFisher Scientific (goat anti-rabbit IgG (H+L): A546: A-11010, AB_2534077; A647: A-21244, AB_2535812; goat anti-chicken IgY (H+L) A488: A32931TR, AB_2866499),goat anti-rabbit A555 (A-11008), goat anti-chicken A488 (A-11039), goat anti-rat A555 (A-21434), goat anti-rat 647 (A-21247), goat anti-mouse IgGgamma1 647 (A-21240), Goat anti-Rabbit IgG (1:100; #35401; CST).
Validation	rabbit anti-JUNB (1:100; 10486-1-AP; lot #00039797, Proteintech) was validated in this study (Extended Fig.13b) in addition to previous validations. Please refer to antibodies data-sheet from provider companies for validation for each species. Listed below are the RRIDs for each antibody:<br>Rabbit anti-JUNB (Proteintech, 10486-1-AP, 1:100) – RRID:AB_2129996<br>Mouse anti-SOX2 (Atlas, AMAb91307, clone CL4716, 1:500) – RRID: AB_2665892<br>Rabbit anti-SOX2 (Abcam, ab97959, 1:200) – RRID:AB_2341193<br>Chicken anti-GFP (Aves Labs, GFP-1020, 1:1000) – RRID:AB_10000240<br>Chicken anti-GFP (Abcam, ab13970, 1:1000) – RRID:AB_300798<br>Rabbit anti-NEUROD2 (Abcam, ab109406, clone EPR5135, 1:300) – RRID: AB_10866309<br>Rabbit anti-NEUROD2 (Abcam, ab104430, 1:400) – RRID:AB_10711796

Mouse anti-EOMES (ThermoFisher, WD1928, 1:100) – RRID:AB_2572882
Rat anti-EOMES (Invitrogen, Dan11mag, 1:200) – RRID:AB_11042577
Rat anti-BrdU (Abcam, ab6326, clone BU1/75, 1:500) – RRID: AB_305426
Mouse anti-PAX6 (ThermoFisher, 13B10-1A10, 1:200) – RRID: AB_2536820
Rat anti-RFP (Proteintech, 5F8, clone 5F8, 1:500) – RRID:AB_2336064
Sheep anti-TBR2 (R&D Systems, AF6166, 1:150) – RRID:AB_10569705
Rabbit anti-KI67 (Abcam, ab15580, 1:100) – RRID:AB_443209
Rabbit anti-H3K27ac (Abcam, ab177178, 1:50) – RRID: AB_2828007
Rabbit anti-H3K4me3 (Cell Signaling Technology, #9751, 1:50) – RRID:AB_2616028
Rabbit anti-IRF1 (Proteintech, 11335-1-AP, 1:50) – RRID:AB_2877759
Normal Rabbit IgG (Cell Signaling Technology, #2729, 1:50) – RRID:AB_1031062

Eukaryotic cell lines

Policy information about cell lines and Sex and Gender in Research

Cell line source(s)	iPSCs (HPS00076:409B2 and GM05399 RIKEN; PC1505 - female apparently healthy line bought from Phenocell, now part of Axol Bioscience)
Authentication	iPSCs were authenticated by the original vendors using karyotyping analysis.
Mycoplasma contamination	iPSCs were periodically checked for Mycoplasma contamination and were negative.
Commonly misidentified lines (See ICLAC register)	No misidentified lines were used in this study.

Animals and other research organisms

Policy information about studies involving animals; ARRIVE guidelines recommended for reporting animal research, and Sex and Gender in Research

Laboratory animals	CD1 pregnant adult female mice from Charles River Laboratory were used. Mice were maintained in house under standard housing conditions 12 h:12 h light:dark cycles with food and water ad libitum at temperature at 21±1°C and relative humidity at 55±5%.
Wild animals	No wild animals were used in this study.
Reporting on sex	Due to randomization of the electroporations and early collection ages, sex was balanced by pooling multiple biological replicates together for single cell/nuclei experiments.
Field-collected samples	No field-collected samples were used in this study.
Ethics oversight	The experimental procedures described here were conducted in accordance with the Swiss laws and previously approved by the Geneva Cantonal Veterinary Authority.

Note that full information on the approval of the study protocol must also be provided in the manuscript.

Plants

Seed stocks	N/A
Novel plant genotypes	N/A
Authentication	N/A

ChIP-seq

Data deposition

☒ Confirm that both raw and final processed data have been deposited in a public database such as GEO.

☒ Confirm that you have deposited or provided access to graph files (e.g. BED files) for the called peaks.

Data access links *May remain private before publication.*	CUT&Tag/RUN data is deposited on GSE324846.

Files in database submission	N/A
Genome browser session (e.g. UCSC)	N/A

Methodology

Replicates	2 biological replicates were pooled for CUT&Tag/RUN on organoids. Five or four electroporated cortices were pooled for Control or JUNB overexpression, respectively.
Sequencing depth	CUT&Tag<br>Control H3K27ac 6'958'587<br>JUNBOE H3K27ac 4'427'166<br>Organoids Junb 20'303'279<br>Organoids RabbitIgG 967'738<br><br>CUT&RUN<br>Organoids H3K4me3 36'423'410<br>Organoids Irf1 47'133'776<br>Organoids RabbitIgG 35'856'583
Antibodies	CUT&RUN: Rabbit anti-H3K4me3 (1:50; #9751; lot #; CST), Rabbit anti-IRF1 (1:50; 11335-1-AP; lot #; Proteintech), Normal Rabbit IgG (1:50; #2729; lot #11; CST).<br>CUT&Tag: rabbit anti-JUNB (1:100; AB_2129996; #10486-1-AP; Proteintech), Normal Rabbit IgG (1:50; #2729; #11; CST).
Peak calling parameters	Using SEACR, 0.01 threshold of pvalue was used as a cutoff.
Data quality	4DN CUT&RUN processing pipeline (Reiff, S. B. et al. 2022) was used to include the top peaks in the analysis, detailed information is outlined in the methods.
Software	All code used for the analysis in deposited on https://github.com/awaisj14/Humous/.

Flow Cytometry

Plots

Confirm that:

☒ The axis labels state the marker and fluorochrome used (e.g. CD4-FITC).

☒ The axis scales are clearly visible. Include numbers along axes only for bottom left plot of group (a 'group' is an analysis of identical markers).

☒ All plots are contour plots with outliers or pseudocolor plots.

☒ A numerical value for number of cells or percentage (with statistics) is provided.

Methodology

Sample preparation	For single cell experiments, electroporated cortices were dissected, dissociated and sorted based on the electroporated reporter plasmid TurboRFP and forward/side scatter. For single nuclei experiments, electroporated cortices were dissected, dissociated and sorted based on Hoechst+ signal and electroporated reporter H2B-GFP.
Instrument	Beckman Coulter Moflo Astrios FAC-sorter (single nuclei); BD FACSMelody Cell Sorter (single cell).
Software	SUMMIT 6.3.1; BD FACSChorus
Cell population abundance	Electroporated cells/nuclei were rare (0.5-5%) of the total sample. 50K cells for Junb and 18K cells for control were sorted for single cell experiments and 25K nuclei were sorted for both conditions in the single nuclei experiments.
Gating strategy	Forward and side scatter was used to gate both nuclei and cells. Singlets were then gated with either RFP+ (single cell) or Hoechst+ GFP+ (single nuclei).

☒ Tick this box to confirm that a figure exemplifying the gating strategy is provided in the Supplementary Information.
